# Coupling biocatalysis with high-energy flow reactions for the synthesis of carbamates and β-amino acid derivatives

**DOI:** 10.3762/bjoc.17.33

**Published:** 2021-02-04

**Authors:** Alexander Leslie, Thomas S Moody, Megan Smyth, Scott Wharry, Marcus Baumann

**Affiliations:** 1 School of Chemistry, University College Dublin, D04 N2E2, Ireland; 2 Almac Group Ltd., Craigavon BT63 5QD, United Kingdom; 3 Arran Chemical Company, Athlone, Co. Roscommon N37 DN24, Ireland

**Keywords:** biocatalysis, CALB, Curtius rearrangement, flow synthesis, reaction telescoping

## Abstract

A continuous flow process is presented that couples a Curtius rearrangement step with a biocatalytic impurity tagging strategy to produce a series of valuable Cbz-carbamate products. Immobilized CALB was exploited as a robust hydrolase to transform residual benzyl alcohol into easily separable benzyl butyrate. The resulting telescoped flow process was effectively applied across a series of acid substrates rendering the desired carbamate structures in high yield and purity. The derivatization of these products via complementary flow-based Michael addition reactions furthermore demonstrated the creation of β-amino acid species. This strategy thus highlights the applicability of this work towards the creation of important chemical building blocks for the pharmaceutical and speciality chemical industries.

## Introduction

Continuous flow chemistry is by now a mature field with chemists in both academia and industry regularly reporting on the multitude of benefits arising from exploiting reactor miniaturization [1–5]. The steady increase of applications highlighting improved syntheses is thereby paralleled by a growing appreciation of pitfalls and challenges [6–7] as well as solutions for their rectification that result from increasing knowledge and experience [8]. Pleasingly, recent years have also witnessed the expansion of flow chemistry in university curricula [9] although considerable efforts are still needed to provide future chemists with hands-on experience in modern synthesis technologies.

Apart from increased safety and scalability, reaction telescoping is one of the most attractive features of flow synthesis allowing for streamlined routes to minimize the need for the isolation and handling of potentially unstable or harmful intermediates [10–13]. Importantly, recent applications have extended from telescoping chemical transformations to also integrate bioassays [14], inline purification [15] and formulation stages [16]. This development is accompanied by exploiting biocatalyzed transformations within continuous flow processes [17–20] to provide a greener and more chemoselective means for the synthesis of drug-like targets. Recently, we reported on an innovative telescoped process using immobilized CALB (*Candida antarctica* lipase B) to enable the conversion of residual benzyl alcohol into benzyl butyrate in view of facilitating the downstream purification of continuous flow Curtius rearrangement reactions [21]. In this paper, we will give a full account on this valuable approach and showcase the utility of the carbamate products towards generating sets of β-amino acid species.

## Results and Discussion

**Scheme 1:**
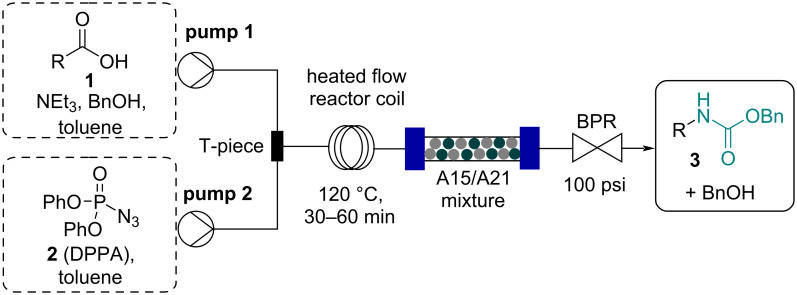
The continuous flow set-up used.

Flow investigations commenced with the development of a continuous Curtius rearrangement process based on previous reports [22–24]. Specifically, we targeted utilization of a range of commercially available carboxylic acids **1** (1.0 equiv) in combination with DPPA (**2**, 0.9 equiv) as the azide donor to facilitate the generation and immediate use of the intermediate acyl azide that would rearrange to an isocyanate upon heating. Toluene was chosen as a suitable solvent providing good solubility of the acid substrates (1 M) in the presence of triethylamine (1.0 equiv). Furthermore, a backpressure regulator (Upchurch, 100 psi) allowed for superheating of the reaction mixture to 120 °C whilst offering control of released nitrogen gas. As the specific target was the generation of a set of Cbz-carbamate products, benzyl alcohol was used as the nucleophile and initial studies indicated that a slight excess of 1.8 equiv was needed to ensure the full and rapid conversion of the isocyanate intermediate into the desired Cbz-carbamate product **3**. A Vapourtec E-series flow platform was used to operate this continuous process as depicted in Scheme 1.

**Figure 1:**
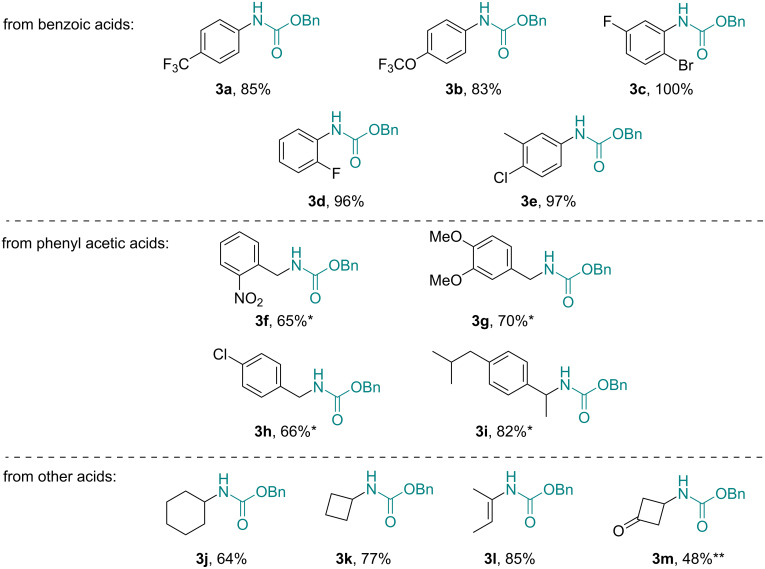
Scope of Cbz-carbamate products obtained via flow process (*t_Res_ = 60 min, **T = 80 °C; isolated yields after removal of BnOH, vide infra).

In order to rapidly create a small library of these versatile carbamate building blocks (**3**, ca. 1 mmol scale), we decided to apply a scavenger column to perform in-line purification. This was achieved as previously reported [22] based on a mixture of Amberlyst A21 (a tertiary amine, ca. 2 equiv) and Amberlyst A15 (a sulfonic acid, ca. 2 equiv) resins packed into an Omnifit glass column (10 cm length, 10 mm i.d., rt). This approach facilitated the removal of triethylamine, residual acid substrate, and diphenylphosphonic acid (generated from DPPA) and rendered a solution of the carbamate product containing only unreacted benzyl alcohol. A variety of different acid substrates including benzoic acids, phenylacetic acids, and various non-aromatic acids successfully underwent the above continuous Curtius rearrangement process giving the desired Cbz-carbamates in good yields (Figure 1).

**Scheme 2:**
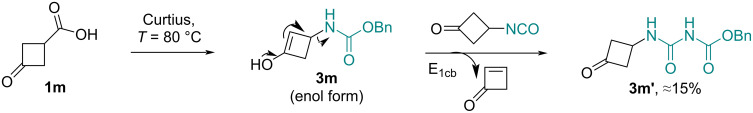
Side reaction during formation of product **3m**.

As expected, benzoic acids cleanly and rapidly rearranged within a residence time of 30 minutes rendering the corresponding carbamates **3a**–**e** in high chemical yields. Phenylacetic acid species were found to be less reactive substrates that rearranged more slowly (*t*_Res_ = 60 min) and thus giving lower yields (**3f**–**h**). Moreover, the formation of urea side products (10–20%) was observed in several cases indicating the competitive attack of the isocyanate by adventitious water followed by the reaction of the resulting amine with a second isocyanate molecule. Consistent with the expectations, substituted phenylacetic acids such as ibuprofen showed an increased migratory aptitude which in turn translated into a higher chemical yield towards the corresponding carbamate structure **3i**. Lastly, a set of non-aromatic acids was subjected to the same reaction conditions providing access to carbamates bearing cyclohexyl, cyclobutyl, and vinylic appendages (**3j**–**m**). An interesting case concerned the use of 3-oxocyclobutanecarboxylic acid (**1m**), which yielded the desired carbamate product **3m** along with the carbamoyl-urea species **3m’**. This observation parallels recent reports [25] proposing a fragmentation of the desired carbamate followed by a combination of the resulting benzyl carbamate with a second molecule of isocyanate (Scheme 2).

Importantly, as the scavenger-based in-line purification approach was successful in removing all acidic or basic species, we decided to not optimize individual Curtius rearrangement reactions, but instead focus on developing an efficient biocatalyzed process for the tagging of residual benzyl alcohol.

**Scheme 3:**
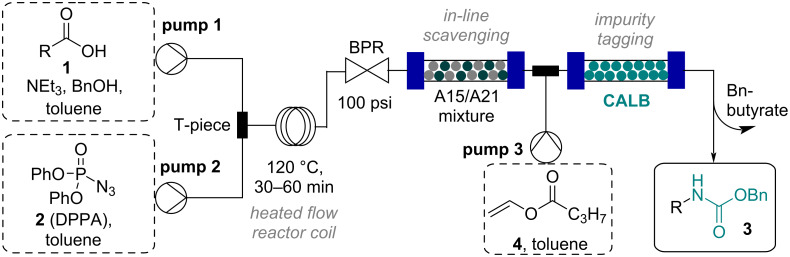
Flow set-up for the CALB-mediated impurity tagging approach.

Initially the removal of benzyl alcohol proved challenging due to its high boiling point (205 °C) and its co-polarity with many of the carbamate products. As this rendered evaporation and column chromatography ineffective, an alternative approach was sought. The possibility to exploit the use of an enzyme to derivatize benzyl alcohol into an easily separable species was an intriguing opportunity. To integrate such a biocatalyzed transformation into the continuous Curtius rearrangement process, immobilized CALB, a robust hydrolase enzyme was utilized as it is frequently used for esterification reactions [26–28]. CALB could convert benzyl alcohol in the presence of vinyl butyrate into benzyl butyrate and acetaldehyde. To demonstrate the feasibility, the flow process was adapted by mixing a stream of vinyl butyrate (**4**, 3 equiv in toluene) via a T-piece with the flow stream exiting the scavenger column (A15/A21). The combined stream was then directed into an Omnifit column (10 cm length, 6.6 mm i.d.) containing immobilized CALB (*t*_Res_ ca. 2–5 min; rt). The flow set-up for the CALB-mediated impurity tagging approach is depicted in Scheme 3.

The telescoped approach provided the quantitative conversion of benzyl alcohol into benzyl butyrate which could be removed by crystallization (from heptanes) after evaporation of toluene and residual vinyl butyrate. The resulting telescoped flow approach thus enabled the facile generation and purification of a small library of valuable Cbz-carbamates (see Figure 1). Furthermore, the CALB column was effectively used over several days after purging with toluene to prevent cross contamination. The scale-up of this flow process was demonstrated on a 100 mmol scale showcasing the robust nature of this innovative approach (85% yield, for details see [21]).

**Scheme 4:**
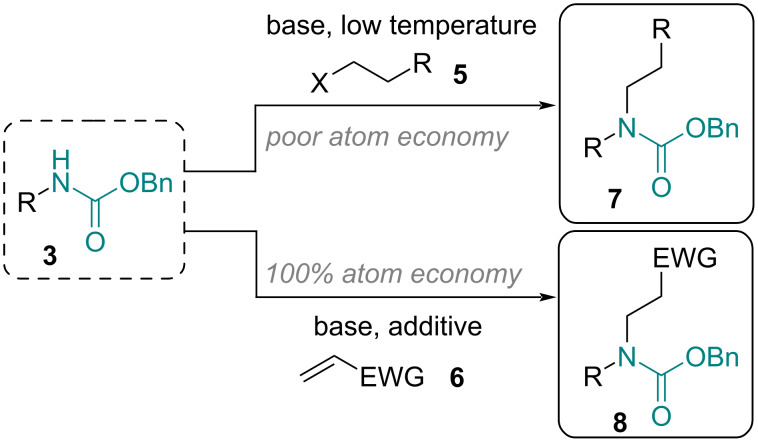
Strategies towards accessing β-amino acid derivatives **8**.

To further exploit the value of this continuous flow approach the possibility of converting selected carbamate products into derivatives of β-amino acids (e.g., **8**) was evaluated. Although this may be achieved by a conventional alkylation of the carbamate nitrogen with a suitable electrophile, the use of a strong base (e.g., NaH) in combination with the chemical waste being generated (from the leaving group), and potentially unfavorable polar aprotic solvents (DMF, NMP) prompted consideration of a more atom-economical approach. The follow-on work focused on utilizing electron-poor alkenes as the reaction partners that would undergo aza-Michael addition reactions on the Cbz-carbamates (Scheme 4).

**Scheme 5:**
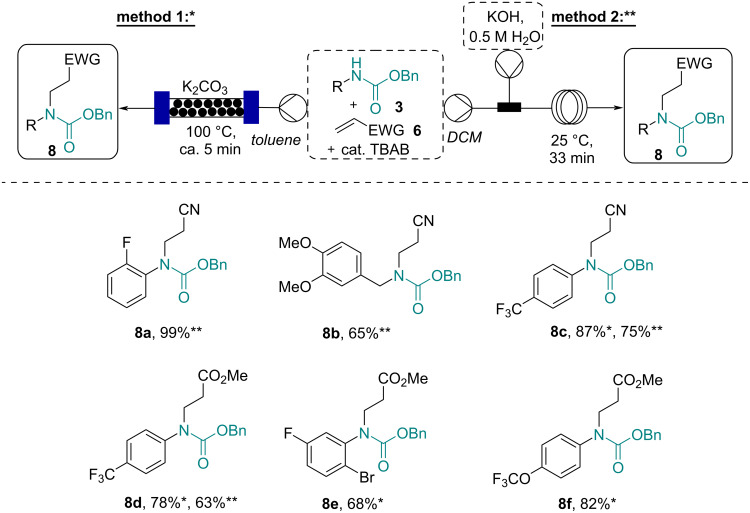
Complementary flow approaches towards the β-amino acid derivatives **8**.

Driven by the desire to develop readily scalable routes towards the target products **8**, continuous flow processing was again exploited. In a first approach the use of solid K_2_CO_3_ packed into an Omnifit glass column (10 cm length, 10 mm i.d., filled with powdered K_2_CO_3_) was trialed as a heterogeneous reagent. A simple flow process was quickly realized in which a solution of the carbamate substrate (0.5 M toluene, 1.0 equiv) containing either acrylonitrile or methyl acrylate (1.2 equiv) was pumped at a flow rate of 0.5 mL/min through the K_2_CO_3_ column heated to 100 °C. The resulting short residence time (≈5 min) within the column was sufficient to give a high conversion of the substrates tested (Scheme 5, method 1).

**Scheme 6:**
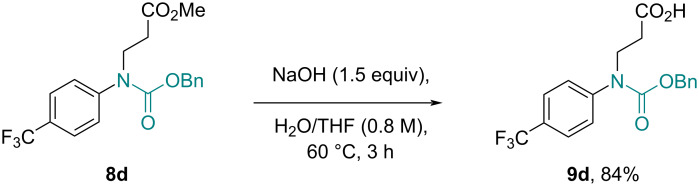
Batch hydrolysis of the ester group in the presence of the carbamate.

In a complementary approach a biphasic liquid–liquid system was tested (Scheme 5, method 2). A stream (0.15 mL/min) containing the carbamate product (0.5 M DCM, 1.0 equiv), the Michael acceptor (1.2 equiv), and tetrabutylammonium bromide (TBAB, 0.1 equiv) [29] was thereby mixed in a T-piece with a stream of aqueous KOH (50 wt %, 0.15 mL/min) creating a biphasic plug flow pattern as the material progressed through a tubular flow coil reactor (rt, 33 min residence time). Upon separation of the phases and evaporation of the volatiles, the desired products were obtained in generally high yields. It was found that both approaches worked well for the tested substrates including carbamates derived from benzoic acids and phenylacetic acids. Initial test reactions with non-aromatic substrates (e.g., **3j** with acrylonitrile using method 2) were however unsuccessful indicating a diminished reactivity of such substrates under these conditions. Whilst the approach using a packed column with K_2_CO_3_ is based on a simple set-up and issues due to channeling were not observed, the use of forcing conditions may not tolerate more sensible functionalities. As such, the phase-transfer-catalyzed process may be preferable in these cases due to its mild conditions which furthermore allowed the ester/nitrile moieties to be unaffected. Indeed, it was established subsequently that alkaline hydrolysis of the ester group requires a prolonged reaction time at an elevated temperature (Scheme 6). Importantly this can be achieved selectively without a concomitant cleavage of the carbamate group allowing for further use of the resulting *N*-protected amino acid species (e.g., **9d**) in synthetic elaborations.

## Conclusion

In conclusion, a new strategy in continuous flow processing that combines challenging high-energy transformations with downstream impurity tagging facilitated by immobilized CALB enzyme is reported. This approach enabled the chemoselective derivatization of benzyl alcohol into the readily removable benzyl butyrate thus simplifying the final purification stages. The resulting Cbz-carbamates were furthermore elaborated into derivatives of β-amino acids via biphasic flow-based aza-Michael addition reactions. This novel approach allows for the creation of important chemical building blocks whilst demonstrating a new use of biocatalysts in continuous flow processes.

## Supporting Information

File 1 Experimental details and spectroscopic data.
