# Supplementary material for: Coupling biocatalysis with high-energy flow reactions for the synthesis of carbamates and β-amino acid derivatives
Source: Beilstein J Org Chem. 2021 Feb 4;17:379–84. doi: 10.3762/bjoc.17.33 (PMC7871027; doi:10.3762/bjoc.17.33)
Supplement: File 1 — Experimental details and spectroscopic data. [file Beilstein_J_Org_Chem-17-379-s001.pdf]

BEILSTEIN JOURNAL OF ORGANIC CHEMISTRY

Supporting Information

for

Coupling biocatalysis with high-energy flow reactions for the synthesis of carbamates and β-amino acid derivatives

Alexander Leslie, Thomas S. Moody, Megan Smyth, Scott Wharry and Marcus Baumann

Beilstein J. Org. Chem. **2021,** *17,* 379–384. doi:10.3762/bjoc.17.33

Experimental details and spectroscopic data

License and Terms: This is a supporting information file under the terms of the Creative Commons Attribution License (https://creativecommons.org/licenses/by/4.0). Please note that the reuse, redistribution and reproduction in particular requires that the author(s) and source are credited and that individual graphics may be subject to special legal provisions.
The license is subject to the *Beilstein Journal of Organic Chemistry* terms and conditions: (https://www.beilstein-journals.org/bjoc/terms)

Table of contents

1. Materials and methods......S2

2. Experimental details and spectroscopic data S3

3. Copies of NMR spectra S11

1. Materials and methods

Unless otherwise stated, all solvents were purchased from Fisher Scientific and were used without further purification. Substrates and reagents were purchased from Fluorochem or Sigma Aldrich and used as received. Immobilised CALB enzyme was supplied by Almac and can be purchased from Strem Chemicals (CAS 9001-62-1).

^{1}H NMR spectra were recorded on 400 MHz, 500 MHz, and 600 MHz instruments and are reported relative to residual solvent: $CDCl_3$ (δ 7.26 ppm) or d_6-DMSO (δ 2.50 ppm). ^{13}C NMR spectra were recorded on the same instruments (100, 125, and 150 MHz) and are reported relative to $CHCl_3$ (δ 77.16 ppm) or d_6-DMSO (δ 39.52 ppm). ^{19}F NMR spectra were recorded at 376 MHz and 470 MHz. The data for ^{1}H NMR are reported as follows: chemical shift (δ/ ppm) (integration, multiplicity, coupling constant (Hz)). Multiplicities are reported as follows: s = singlet, d = doublet, t = triplet, q = quartet, p = pentet, m = multiplet, br. s = broad singlet, app = apparent. Data for ^{13}C{^{1}H} NMR are reported in terms of chemical shift (δ/ ppm) and multiplicity (C, CH, CH_2 or CH_3). DEPT-135, COSY, HSQC, HMBC, and NOESY experiments were used in the structural assignment.

IR spectra were obtained by use of a Bruker Platinum spectrometer (neat, ATR sampling) with the intensities of the characteristic signals being reported as weak (w, <20% of tallest signal), medium (m, 21–70% of the tallest signal) or strong (s, > 71% of the tallest signal).

High-resolution mass spectrometry was performed using the indicated techniques on a micromass LCT orthogonal time-of-flight mass spectrometer with leucine-enkephalin (Tyr-Gly-Phe-Leu) as an internal lock mass.

Continuous flow experiments were performed on a Vapourtec E-series system using PFA reactor coils (10 mL volume, i.d. 1/16 inch) in combination with Omnifit glass columns.

2. Experimental details and spectroscopic data

General procedure for Curtius rearrangements in flow:

In a similar manner as described before [*Org. Process Res. Dev.* **2020**, in press; doi.org/10.1021/acs.oprd.0c00420] the carboxylic acid substrate (1.0 equiv), triethylamine (1.0 equiv.) and benzyl alcohol (1.8 equiv) are dissolved in toluene giving after agitation a clear solution (1 M). DPPA (0.9 equiv) is dissolved in toluene giving a second solution of equal volume (0.9 M). Both solutions are pumped at individual flow rates of 0.17 mL/min and mixed in a T-piece (1/8'' PEEK) before entering a reactor coil mounted on a Vapourtec flow reactor (10 mL, PFA, 120 °C). Upon exiting the reactor coil the crude Curtius reaction mixture passed a BPR (100 psi) before entering an Omnifit column filled with a mixture of scavenger resin (A15/A21, ca. 50:50).

General procedure of telescoped use of immobilised CALB:

In a similar manner as described before [*Org. Process Res. Dev.* **2020**, in press; doi.org/10.1021/acs.oprd.0c00420] the resulting solution is mixed via a further T-piece with a stream of vinyl butyrate (3 equiv in toluene, 0.34 mL/min). The combined mixture was then directed into an Omnifit column (e.g., 100 mm length, 6.6 mm i.d.) containing immobilized CALB (ambient temperature, ca. 1.0 g, estimated residence time 2–5 minutes). Upon collection, the combined reaction mixture was evaporated and then redissolved in EtOAc before extraction with water. The crude product obtained after evaporation was crystallised from cold heptanes to yield the crystalline target products. For non-solid products, further purification can be achieved by silica column chromatography (10–20% EtOAc/cyclohexane).

Spectroscopic data:

Benzyl (4-(trifluoromethyl)phenyl)carbamate (3a):

Please see prior report for spectroscopic data and copies of NMR spectra [*Org. Process Res. Dev.* **2020**, in press; doi.org/10.1021/acs.oprd.0c00420].

Benzyl (4-(trifluoromethoxy)phenyl)carbamate (3b):

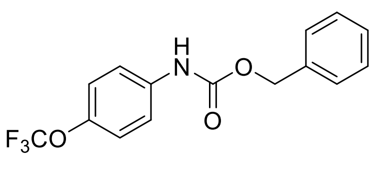

Chemical Formula: $C_{15}H_{12}F_3NO_3$
Exact Mass: 311.0769

Yield: 83% (258 mg, 0.83 mmol)
Appearance: white solid

^{1}H-NMR (400 MHz, CDCl$_3$) δ/ppm 7.46–7.32 (m, 5H), 7.13 (d, *J* = 8.0 Hz, 2H), 6.89 (br s, 1H), 5.19 (s, 2H). **^{13}C-NMR (100 MHz, CDCl$_3$)** δ/ppm 153.4 (C), 144.8 (C), 136.5 (C), 135.8 (C), 128.6 (2CH), 128.5 (CH), 128.3 (2CH), 121.8 (2CH), 120.5 (q, *J* = 258 Hz, OCF$_3$), 119.8 (br s, 2CH), 67.2 (CH$_2$). **^{19}F-NMR (376 MHz, CDCl$_3$)** δ/ppm -58.2 (s). **IR (neat)** ν/cm^{-1}: 3333 (m), 1703 (m), 1528 (s), 1414 (w), 1216 (s), 1148 (s), 1064 (m), 734 (m), 606 (m), 510 (m). **HR-MS** (TOF ES+) calcd for $C_{15}H_{13}F_3NO_3$ 312.0842, found 312.0844 (M+H$^+$).

Benzyl (2-bromo-5-fluorophenyl)carbamate (3c):

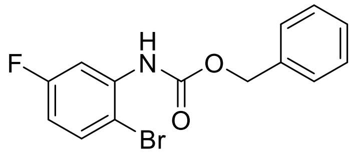

Chemical Formula: $C_{14}H_{11}BrFNO_2$
Exact Mass: 322.9957

Yield: 100% (320 mg, 1.0 mmol).
Appearance: cream solid.

^{1}H-NMR (400 MHz, CDCl$_3$) δ/ppm 8.07 – 8.02 (dd, *J* = 11.2 Hz, 2.9 Hz, 1H), 7.46 – 7.33 (m, 6H), 7.27-7.20 (m, 1H), 6.69-6.63 (ddd, *J* = 8.8 Hz, 7.5 Hz, 3.0 Hz, 1H), 5.23 (s, 2H). **^{13}C-NMR (100 MHz, CDCl$_3$)** δ/ppm 162.4 (d, *J* = 244 Hz, C), 152.7 (C), 137.0 (d, *J* = 12 Hz, C), 135.5 (C), 132.9 (d, *J* = 9 Hz , CH), 128.7 (2CH), 128.6 (CH), 128.5 (2CH), 111.2 (d, *J* = 23, CH), 107.5 (d, *J* = 30 Hz, CH), 106.1 (d, *J* = 3 Hz, C), 67.6 (CH_2). **^{19}F-NMR (376 MHz, CDCl$_3$)** δ/ppm -111.2 (m). **IR (neat)** ν/cm^{-1}: 3264 (m), 1695 (s), 1525 (s), 1243 (s), 1230 (s), 1167 (s), 1030 (m), 953 (m), 746 (s), 588 (s). **HR-MS** (TOF ES+) calcd for $C_{14}H_{12}BrFNO_2$ 324.0030, found 324.0030 (M+H$^+$).

Data is consistent with previous reports: DOI: 10.1002/anie.201402661

Benzyl (2-fluorophenyl)carbamate (3d):

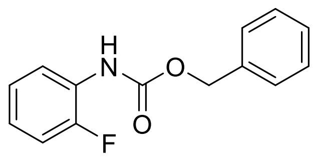

Chemical Formula: $C_{14}H_{12}FNO_2$
Exact Mass: 245.0852

Yield: 96% (235 mg, 0.96 mmol).
Appearance: white solid

^{1}H-NMR (400 MHz, CDCl$_3$) δ/ppm 8.12 (br tr, *J* = 7.1 Hz, 1H), 7.43-7.32 (m, 5H), 7.12 (td, *J* = 8.0 Hz, 0.78 Hz, 1H), 7.09 – 6.96 (m, 2H), 6.94 (br s, 1H), 5.22 (s, 2H). **^{13}C-NMR (100 MHz, CDCl$_3$)** δ/ppm 153.0 (C), 152.1 (d, *J* = 241 Hz, C), 135.8 (C), 128.6 (2CH), 128.5 (CH), 128.4 (2CH), 126.3 (d, *J* = 10 Hz, C), 124.6 (d, *J* = 4 Hz, CH), 123.4 (d, *J* = 7 Hz, CH), 120.2 (CH), 114.8 (d, *J* = 19 Hz, CH), 67.3 (CH_2). **^{19}F-NMR (282 MHz, CDCl$_3$)** δ/ppm -132.4 (br s). **IR (neat)** ν/cm^{-1}: 3281 (m), 1693 (s), 1597 (w), 1519 (s), 1498 (s), 1304 (s), 1109 (w), 1027 (m), 740 (s), 580 (m). **HR-MS** (TOF ES+) calcd for $C_{14}H_{13}FNO_2$ 246.0925, found 246.0928 (M+H$^+$).

Data is consistent with previous reports: Doi.org/10.1016/j.tetlet.2008.05.010

Benzyl (4-chloro-3-methylphenyl)carbamate (3e):

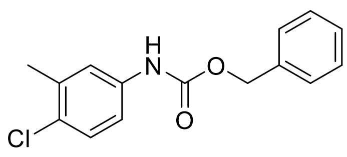

Chemical Formula: $C_{15}H_{14}ClNO_2$
Exact Mass: 275.0713

Yield: 97% (267 mg, 0.97 mmol)
Appearance: white solid

^{1}H-NMR (400 MHz, CDCl$_3$) δ/ppm 7.45-7.35 (m, 5H), 7.34-7.30 (m, 1H), 7.29-7.27 (s, 1H), 7.16 (dd, *J* = 11.5 Hz, 4.3 Hz, 1H), 6.61 (br s, 1H), 5.22 (m, 2H), 2.37 (s, 3H). **^{13}C-NMR (100 MHz, CDCl$_3$)** δ/ppm 153.3 (C), 136.7 (C), 136.3 (C), 135.9 (C), 129.4 (CH), 128.8 (C), 128.6 (2CH), 128.4 (CH), 128.3 (2CH), 121.0 (CH), 117.4 (CH), 67.1 (CH_2), 20.2 (CH_3). **IR (neat)** ν/cm^{-1}: 3274 (m), 1693 (s), 1609 (m), 1537 (s), 1305 (w), 1229 (s), 1078 (s), 883 (m), 814 (w), 737 (s). **HR-MS** (TOF ES+) calcd for $C_{15}H_{15}ClNO_2$ 276.0786, found 276.0790 (M+H$^+$).

Benzyl (2-nitrobenzyl)carbamate (3f):

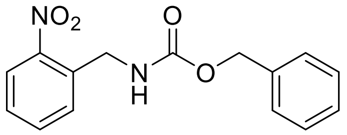

Chemical Formula: $C_{15}H_{14}N_2O_4$
Exact Mass: 286.0954

Yield: 65% (406 mg, 1.42 mmol)
Appearance: yellow solid.

^{1}H-NMR (400 MHz, $CDCl_3$) δ/ppm 8.06 (d, *J* = 8.5 Hz, 1H), 7.67-7.58 (m, 2H), 7.45 (td, J = 7.5 Hz, 2.2 Hz, 1H), 7.37-7.27 (m, 5H), 5.60 (br tr, 1H), 5.09 (s, 2H), 4.62 (d, *J* = 6.7 Hz, 2H). **^{13}C-NMR (100 MHz, $CDCl_3$)** δ/ppm 156.4 (C), 136.2 (C), 134.1 (CH), 134.0 (C), 131.9 (CH), 129.2 (C), 128.8 (CH), 128.5 (2CH), 128.2 (CH), 128.1 (2CH), 125.2 (CH), 67.0 (CH_2), 42.9 (CH_2). **IR (neat)** ν/cm^{-1}: 3310 (m), 1681 (s), 1536 (s), 1510 (s), 1342 (m), 1217 (s), 967 (m), 832 (m), 698 (s), 474 (m). **HR-MS** (TOF ES+) calcd for $C_{15}H_{15}N_2O_4$ 287.1026, found 287.1030 ($M+H^+$).

Data is consistent with previous reports: *J. Org. Chem.* **2018**, *83*, 2802-2807.

Benzyl (3,4-dimethoxybenzyl)carbamate (3g):

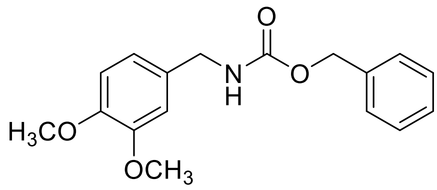

Chemical Formula: $C_{17}H_{19}NO_4$
Exact Mass: 301.1314

Yield: 70% (438 mg, 1.46 mmol).
Appearance: white solid.

^{1}H-NMR (400 MHz, $CDCl_3$) δ/ppm 7.36-7.27 (m, 5H), 6.81-6.77 (m, 3H), 5.12 (s, 2H), 4.99 (br s, 1H), 4.30 (d, *J* = 5.9 Hz, 2H), 3.86-3.82 (m, 6H). **^{13}C-NMR (125 MHz, $CDCl_3$)** δ/ppm 156.4 (C), 149.1 (C), 148.5 (C), 136.5 (C), 131.0 (C), 128.5 (3CH), 128.2 (2CH), 119.8 (CH), 111.2 (CH), 110.9 (CH), 66.8 (CH_2), 56.0 (CH_3), 55.9 (CH_3), 45.0 (CH_2). **IR (neat)** ν/cm^{-1}: 3330 (m), 2966 (w), 2153 (w), 1678 (m), 1592 (w), 1514 (s), 1230 (s), 1138 (s), 1021 (m), 648 (m). **HR-MS** (TOF ES+) calcd for $C_{17}H_{19}NO_4Na$ 324.1206, found 324.1210 ($M+Na^+$).

Benzyl (4-chlorobenzyl)carbamate (3h):

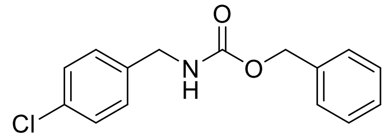

Chemical Formula: $C_{15}H_{14}ClNO_2$
Exact Mass: 275.0713

Yield: 66% (510 mg, 1.85 mmol)
Appearance: white solid

^{1}H-NMR (400 MHz, $CDCl_3$) δ/ppm 7.36-7.25 (m, 7H), 7.20 (br s, 1H), 7.18 (br s, 1H), 5.12 (br s, 1H), 5.11 (s, 2H), 4.32 (d, *J* = 6.2 Hz, 2H). **^{13}C-NMR (100 MHz, $CDCl_3$)** δ/ppm 156.4 (C), 137.0 (C), 136.3 (C), 133.3 (C), 128.8 (2CH), 128.8 (3CH), 128.5 (2CH), 128.2 (CH), 128.1 (CH), 67.0 (CH_2), 44.4 (CH_2). **IR (neat)** ν/cm^{-1}: 3282 (m), 1678 (s), 1542 (m), 1489 (m), 1254 (s), 978 (w), 802 (m), 746 (m), 693 (s), 471 (m). **HR-MS** (TOF ES+) calcd for $C_{15}H_{15}ClNO_2$ 276.0786, found 276.0789 ($M+H^+$).

Data is consistent with previous reports: Doi.org/10.1002/ejoc.200700627

Benzyl (1-(4-isobutylphenyl)ethyl)carbamate (3i):

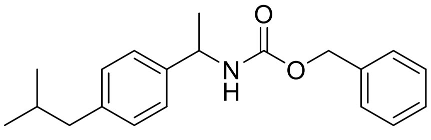

Chemical Formula: $C_{20}H_{25}NO_2$
Exact Mass: 311.1885

Yield: 82% (278 mg, 0.90 mmol)
Appearance: white solid

^{1}H-NMR (400 MHz, $CDCl_3$) δ/ppm 7.36 - 7.27 (m, 5H), 7.22 – 7.17 (m, 2H), 7.13 – 7.08 (m, 2H), 5.11 (d, *J* = 12.0 Hz, 1H), 5.05 (d, *J* = 12.0 Hz, 1H), 5.02 (br s, NH), 4.84 (br m, 1H), 2.45 (d, *J* = 7.2 Hz, 2H), 1.84 (septet, *J* = 6.8 Hz, 1H), 1.48 (d, *J* = 7.6 Hz, 3H), 0.90 (d, *J* = 6.7 Hz, 6H). **^{13}C-NMR (100 MHz, $CDCl_3$)** δ/ppm 155.5 (C), 140.8 (C), 140.6 (C), 136.5 (C), 129.5 (2CH), 128.5 (2CH), 128.1 (2CH+CH), 125.8 (2CH), 66.7 (CH_2), 50.4 (CH), 45.0 (CH_2), 30.2 (CH), 22.4 (2+1CH_3). **IR (neat)** ν/cm^{-1}: 3343 (m), 2959 (m), 1682 (s), 1529 (s), 1453 (w), 1319 (w), 1248 (s), 1081 (m), 845 (w), 642 (m). **HR-MS** (TOF ES+) calcd for $C_{20}H_{26}NO_2$ 312.1958, found 312.1961 (M+H$^+$).

Benzyl cyclohexylcarbamate (3j):

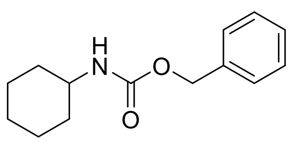

Chemical Formula: $C_{14}H_{19}NO_2$
Exact Mass: 233.1416

Yield: 64% (140 mg, 0.64 mmol)
Appearance: white solid

^{1}H-NMR (400 MHz, $CDCl_3$) δ/ppm 7.36 – 7.27 (m, 5H), 5.07 (s, 2H), 4.60 (br s, 1H), 3.48 (br s, 1H), 1.92 (dd, *J* = 12.6 Hz, 3.4 Hz, 2H), 1.68 (dt, *J* = 14.5 Hz, 3.8 Hz, 2H), 1.62 – 1.54 (m, 1H), 1.30-1.37 (m, 2H), 1.17 – 1.08 (m, 3H). **^{13}C-NMR (100 MHz, $CDCl_3$)** δ/ppm 155.5 (C), 136.7 (C), 128.5 (3CH), 128.1 (CH), 128.0 (CH), 66.4 (CH_2), 49.9 (CH), 33.4 (CH_2), 25.5 (2CH_2), 24.7 (2CH_2). **IR (neat)** ν/cm^{-1}: 3317 (m), 2931 (m), 2853 (m), 1684 (s), 1537 (s), 1452 (w), 1310 (m), 1231 (s), 1045 (s), 693 (s). **HR-MS** (TOF ES+) calcd for $C_{14}H_{20}NO_2$ 234.1489, found 234.1492 (M+H$^+$).

Data is consistent with previous reports: Doi.org/10.1002/ejoc.200700627

Benzyl cyclobutylcarbamate (3k):

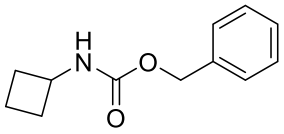

Chemical Formula: $C_{12}H_{15}NO_2$
Exact Mass: 205.1103

Yield: 77% (357 mg, 1.74 mmol)
Appearance: white solid

^{1}H-NMR (400 MHz, $CDCl_3$) δ/ppm 7.27-7.35 (m, 5H), 5.06 (s, 2H), 4.94 (br s, NH), 4.09-4.20 (m, 1H), 2.25-2.33 (m, 2 H), 1.75-1.85 (m, 2H), 1.60-1.70 (m, 2H). **^{13}C-NMR (100 MHz, $CDCl_3$)** δ/ppm 155.2 (C), 136.6 (C), 128.5 (2CH), 128.1 (2+1CH), 66.5 (CH_2), 46.2 (CH), 31.4 (2CH_2), 14.7 (CH_2). **IR (neat)** ν/cm^{-1}: 3315 (s), 2977 (m), 2964 (m), 2867 (m), 1683 (s), 1536 (s), 1453 (m), 1349 (m), 1269 (s), 1214 (s), 1042 (s), 693 (s). **HR-MS** (TOF ES+) calcd for $C_{12}H_{16}NO_2$ 206.1176, found 206.1175 (M+H$^+$).

Benzyl (*E*)-but-2-en-2-ylcarbamate (3l):

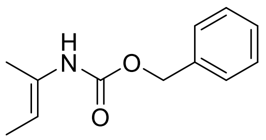

Chemical Formula: $C_{12}H_{15}NO_2$
Exact Mass: 205.1103

Yield: 85% (367 mg, 1.80 mmol)
Appearance: white solid

^{1}H-NMR (500 MHz, $CDCl_3$) δ/ppm 7.30-7.39 (m, 5H), 5.88 (br s, NH), 5.55 (br q, *J* = 8.0 Hz), 5.10 (s, 2H), 1.85 (s, 3H), 1.63 (d, *J* = 8.0 Hz, 3H). **^{13}C-NMR (125 MHz, $CDCl_3$)** δ/ppm 153.8 (C), 136.4 (C), 130.3 (C), 128.5 (2CH), 128.2 (2CH), 128.2 (CH), 109.3 (CH), 66.6 (CH_2), 15.7 (CH_3), 12.4 (CH_3). **IR (neat)** ν/cm^{-1}: 3323 (s), 3033 (w), 2979 (w), 2940 (w), 1704 (s), 1519 (m), 1454 (m), 1386 (m), 1330 (m), 1243 (s), 1214 (s), 1055 (s), 736 (s), 696 (s). **HR-MS** (TOF ES+) calcd for $C_{12}H_{16}NO_2$ 206.1176, found 206.1176 ($M+H^+$).

Benzyl (3-oxocyclobutyl)carbamate (3m):

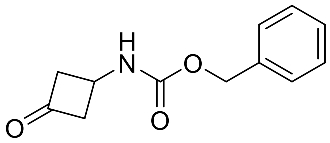

Chemical Formula: $C_{12}H_{13}NO_3$
Exact Mass: 219.0895

Yield: 48% (112 mg, 0.51 mmol)
Appearance: off-white solid

^{1}H-NMR (600 MHz, d_6-DMSO) δ/ppm 8.72 (br d, *J* = 6.0 Hz, NH), 7.32-7.38 (m, 4H), 7.27-7.32 (m, 1H), 5.02 (s, 2H), 4.10-4.20 (m, 1H), 3.24-3.31 (m, 2H), 2.95-3.05 (m, 2H). **^{13}C-NMR (150 MHz, d_6-DMSO)** δ/ppm 206.6 (C), 156.2 (C), 137.4 (C), 128.8 (2CH), 128.3 (2CH+CH), 65.8 (CH_2), 54.3 ($2CH_2$), 36.8 (CH). **IR (neat)** ν/cm^{-1}: 3356 (m), 3035 (w), 2977 (w), 2935 (w), 1780 (s), 1682 (s), 1528 (s), 1262 (s), 1214 (s), 1073 (s), 1012 (s), 752 (s), 729 (s), 694 (s), 571 (s). **HR-MS** (TOF ES+) calcd for $C_{12}H_{14}NO_3$ 220.0968, found 220.0970 ($M+H^+$).

General procedure of continuous Michael addition of carbamates:

Method 1:

Powdered K_2CO_3 was packed into an Omnifit glass column (10 cm length, 10 mm i.d.). Using a Vapourtec E-series system a solution of the carbamate substrate (0.5 M toluene, 1.0 equiv) containing either acrylonitrile or methyl acrylate (1.2 equiv) was pumped at a flow rate of 0.5 mL/min through the K_2CO_3 column heated to 100 °C. A 100 psi back-pressure regulator (Upchurch) was used. The collected product solution was evaporated and purified by silica column chromatography (10–20% EtOAc/cyclohexane) to render the desired target products **8c**, **8d**, **8e**, **8f**.

Method 2:

Using two peristaltic pumps of a Vapourtec E-series flow reactor, a biphasic liquid–liquid system was employed whereby a stream (0.15 mL/min) containing the carbamate product (0.5 M DCM, 1.0 equiv), the Michael acceptor (1.2 equiv), and tetrabutylammonium bromide (TBAB, 0.1 equiv) was mixed in a T-piece with a stream of aqueous KOH (50 wt %, 0.15 mL/min) creating a biphasic plug flow pattern. This was progressed through a tubular flow coil reactor (10 mL, PFA, rt, 33 min residence time) and collected in a beaker. After phase separation the organic layer was evaporated providing the crude product that was purified by silica column chromatography (10–20% EtOAc/cyclohexane) to render the desired target products (**8a**, **8b**, **8c**, **8d**).

Benzyl (2-cyanoethyl)(2-fluorophenyl)carbamate (8a):

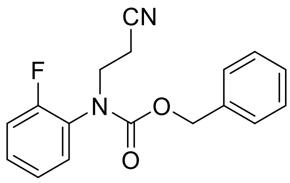

Chemical Formula: $C_{17}H_{15}FN_2O_2$
Exact Mass: 298.1118

Yield: 99% (298 mg, 1.0 mmol)
Appearance: colourless oil
Ratio of rotamers = 77:23.

^{1}H-NMR (400 MHz, CDCl$_3$) δ/ppm 7.45 - 7.10 (m, 9H), 5.30-5.17 (m, 2H, minor), 5.12 (s, 2H, major), 3.88 (t, *J* = 6.92 Hz, 2H), 2.66 (br t, *J* = 6.76 Hz, 2H, major), 2.60-2.50 (m, 2H, minor). **^{13}C-NMR (100 MHz, CDCl$_3$)** δ/ppm 158.1 (d, *J* = 249 Hz, CF), 155.1 (C), 136.0 (C), 129.9 (CH), 129.8 (2CH), 129.7 (2CH), 128.4 (CH), 128.0 (C), 127.3 (CH), 124.9 (d, *J* = 4 Hz, CH), 117.6 (CN), 116.5 (d, *J* = 20 Hz, CH), 68.3 (CH_2, minor), 67.8 (CH_2, major), 46.4 (CH_2, minor), 46.1 (CH_2, major), 17.4 (CH_2, minor), 16.8 (CH_2, major). **^{19}F-NMR (376 MHz, CDCl$_3$)** δ/ppm -120.6 (s, minor), -121.0 (s, major). **IR (neat)** ν/cm^{-1}: 2957 (w), 1705 (s), 1501 (s), 1403 (s), 1303 (s), 1197 (s), 1012 (m), 755 (s), 697 (s), 485 (w). **HR-MS** (TOF ES+) calcd for $C_{17}H_{16}FN_2O_2$ 299.1190, found 299.1193 (M+H$^+$).

Benzyl (2-cyanoethyl)(3,4-dimethoxybenzyl)carbamate (8b):

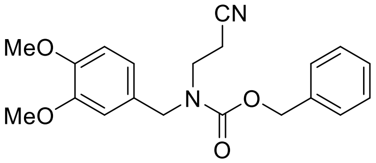

Chemical Formula: $C_{20}H_{22}N_2O_4$
Exact Mass: 354.1580

Yield: 65% (106 mg, 0.30 mmol)
Appearance: colourless oil
Ratio of rotamers: 56:44.

^{1}H-NMR (400 MHz, CDCl$_3$) δ/ppm 7.38 – 7.26 (m, 5H), 6.83 – 6.60 (m, 3H), 5.18 (s, 2H), 4.50 (s, 2H), 3.84 – 3.63 (m, 6H), 3.51 – 3.41 (m, 2H), 2.55 (br t, 2H, major), 2.37 (br t, 2H, minor). **^{13}C-NMR (100 MHz, CDCl$_3$)** δ/ppm 155.9 (C), 149.3 (C), 148.7 (C), 136.2 (C), 129.3 (C), 128.6 (2CH), 128.3 (CH), 128.1 (2CH), 120.4 (CH, minor), 119.9 (CH, major), 118.2 (CN, major), 117.8 (CN, minor), 111.1 (2CH, major),

110.6 (2CH, minor), 67.8 (CH_2, minor), 67.7 (CH_2, major), 55.9 ($2CH_3$, major), 55.7 ($2CH_3$, minor), 51.3 (CH_2), 43.3 (CH_2, major), 42.2 (CH_2, minor), 17.3 (CH_2, minor), 16.6 (CH_2, major). **IR (neat)** ν/cm^{-1}: 2956 (w), 2251 (w), 1703 (s), 1501 (s), 1454 (m), 1402 (s), 1303 (s), 1140 (m), 1025 (m), 754 (s), 697 (s). **HR-MS** (TOF ES+) calcd for $C_{20}H_{23}N_2O_4$ 355.1652, found 355.1653 (M+H$^+$).

Benzyl (2-cyanoethyl)(4-(trifluoromethyl)phenyl)carbamate (8c):

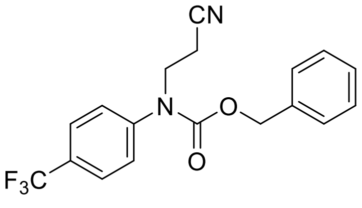

Chemical Formula: $C_{18}H_{15}F_3N_2O_2$
Exact Mass: 348.1086

Yield: 87% (720 mg, 2.07 mmol)
Appearance: colourless solid

^{1}H-NMR (400 MHz, CDCl$_3$) δ/ppm 7.66 – 7.61 (m, 2H), 7.40 – 7.22 (m, 7H), 5.17 (s, 2H), 3.47 (t, *J* = 6.64 Hz, 2H), 2.66 (t, *J* = 6.76 Hz, 2H). **^{13}C-NMR (100 MHz, CDCl$_3$)** δ/ppm 154.6 (C), 144.0 (q, *J* = 2 Hz, C), 135.7 (C), 129.3 (q, *J* = 33 Hz, C), 128.6 (2CH), 128.4 (CH), 127.9 (2CH), 127.4 (2CH), 126.5 (q, *J* = 4 Hz, 2CH), 123.7 (q, *J* = 271 Hz, CF_3), 117.4 (CN), 68.1 (CH_2), 46.3 (CH_2), 17.2 (CH_2). **^{19}F-NMR (376 MHz, CDCl$_3$)** δ/ppm -62.5 (s). **IR (neat)** ν/cm^{-1}: 2957 (w), 2251 (w), 1705 (s), 1615 (m), 1399 (m), 1322 (s), 1108 (s), 1068 (s), 1015 (m), 845 (m), 697 (m). **HR-MS** (TOF ES+) calcd for $C_{18}H_{16}F_3N_2O_2$ 349.1158, found 349.1162 (M+H$^+$).

Methyl 3-(((benzyloxy)carbonyl)(4-(trifluoromethyl)phenyl)amino)propanoate (8d):

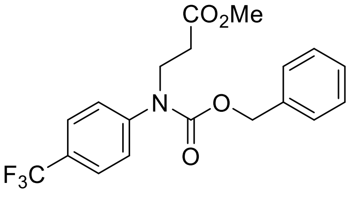

Chemical Formula: $C_{19}H_{18}F_3NO_4$
Exact Mass: 381.1188

Yield: 78% (870 mg, 2.28 mmol)
Appearance: colourless solid

^{1}H-NMR (500 MHz, CDCl$_3$) δ/ppm 7.62 (d, *J* = 8.0 Hz, 2H), 7.27-7.40 (m, 7H), 5.17 (s, 2H), 4.04 (app t, *J* = 5.6 Hz, 2H), 3.57 (s, 3H), 2.61 (app t, *J* = 5.6 Hz, 2H). **^{13}C-NMR (125 MHz, CDCl$_3$)** δ/ppm 171.5 (C), 154.7 (C), 144.6 (C), 136.0 (C), 128.7 (d, *J* = 33 Hz, C), 128.5 (2CH), 128.2 (CH), 127.9 (2CH), 127.2 (2CH), 126.2 (q, *J* = 4 Hz, 2CH), 123.9 (q, *J* = 273 Hz, CF_3), 67.7 (CH_2), 51.7 (CH_3), 46.3 (CH_2), 33.2 (CH_2). **^{19}F-NMR (470 MHz, CDCl$_3$)** δ/ppm -62.5 (s). **IR (neat)** ν/cm^{-1}: 2954 (w), 1739 (s), 1692 (s), 1611 (m), 1449 (m), 1323 (s), 1305 (s), 1109 (s), 752 (m), 700 (m). **HR-MS** (TOF ES+) calcd for $C_{19}H_{19}NF_3O_4$ 382.1261, found 382.1265 (M+H$^+$).

Methyl 3-(((benzyloxy)carbonyl)(2-bromo-5-fluorophenyl)amino)propanoate (8e):

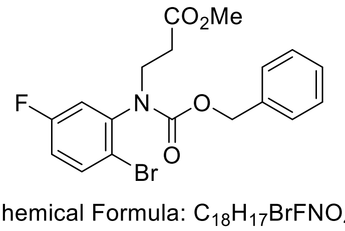

Chemical Formula: $C_{18}H_{17}BrFNO_4$
Exact Mass: 409.0325

Yield: 68% (280 mg, 0.68 mmol)
Appearance: yellow oil
Ratio of rotamers = 77:23

^{1}H-NMR (400 MHz, CDCl$_3$) δ/ppm 7.55 (dd, *J* = 8.88 Hz, 5.68 Hz, 1H), 7.44-7.12 (m, 5H), 7.06-6.87 (m, 2H), 5.28-5.14 (m, 2H, minor), 5.09 (s, 2H, major), 4.09 (ddd, *J* = 14.2 Hz, 14.4 Hz, 7.2 Hz, 1H), 3.74 (ddd, *J* = 14.2 Hz, 14.3 Hz, 7.1 Hz, 1H), 3.60 (s, 3H, major), 3.58 (br s, 3H, minor), 2.74 – 2.58 (m, 2H). **^{13}C-NMR (100 MHz, CDCl$_3$)** δ/ppm 171.7 (C), 161.8 (d, *J* = 248 Hz, CF), 154.6 (C), 141.5 (d, *J* = 10 Hz, C), 136.1 (C), 134.0 (d, *J* = 9, CH), 128.3 (d, *J* = 46 Hz, CH), 128.3 (2CH), 127.9 (CH), 127.5 (CH), 118.3 (d, *J* = 4 Hz, C), 117.8 (d, *J* = 23 Hz, CH), 116.5 (d, *J* = 22 Hz, CH), 67.9 (CH_2, minor) 67.6 (CH_2, major), 51.7 (CH_3), 46.0 (CH_2), 33.5 (CH_2, minor), 32.9 (CH_2, major). **^{19}F-NMR (376**

MHz, $CDCl_3$) δ/ppm -112.7 (m, minor), -112.8 (q, *J* = 7.84 Hz, major). **IR (neat)** ν/cm^{-1}: 2952 (w), 1708 (s), 1597 (w), 1473 (m), 1301 (s), 1171 (s), 1011 (m), 813 (m), 696 (m), 599 (m). **HR-MS** (TOF ES+) calcd for $C_{18}H_{18}BrFNO_3$ 410.0398, found 410.0396 (M+H$^+$).

Methyl 3-(((benzyloxy)carbonyl)(4-(trifluoromethoxy)phenyl)amino)propanoate (8f):

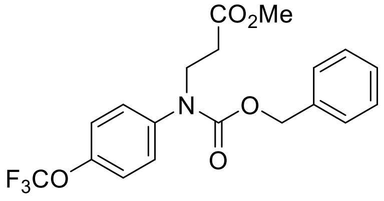

Chemical Formula: $C_{19}H_{18}F_3NO_5$
Exact Mass: 397.1137

Yield: 82% (655 mg, 1.65 mmol)
Appearance: colourless oil.

^{1}H-NMR (400 MHz, $CDCl_3$) δ/ppm 7.15-7.35 (m, 9H), 5.13 (s, 2H), 3.98 (app t, *J* = 8.0 Hz, 2H), 3.55 (s, 3H), 2.58 (app t, *J* = 8.0 Hz, 2H). **^{13}C-NMR (100 MHz, $CDCl_3$)** δ/ppm 171.6 (C), 155.0 (C), 147.5 (C), 139.9 (C), 136.2 (C), 128.7 (br, 2CH), 128.5 (2CH), 128.0 (CH), 127.7 (br, 2CH), 121.6 (2CH), 120.4 (q, *J* = 258 Hz, CF_3), 67.5 (CH_2), 51.6 (CH_3), 46.5 (CH_2), 33.2 (CH_2). **^{19}F-NMR (376 MHz, $CDCl_3$)** δ/ppm -58.0 (s). **IR (neat)** ν/cm^{-1}: 3034 (w), 2954 (w), 1736 (m), 1704 (s), 1509 (s), 1439 (m), 1402 (m), 1252 (s), 1201 (s), 1156 (s), 1066 (m), 1015 (m), 765 (m), 735 (m), 697 (m). **HR-MS** (TOF ES+) calcd for $C_{19}H_{19}NF_3O_5$ 398.1210, found 398.1212 (M+H$^+$).

3-(((Benzyloxy)carbonyl)(4-(trifluoromethyl)phenyl)amino)propanoic acid (9d):

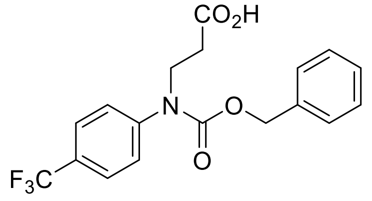

Chemical Formula: $C_{18}H_{16}F_3NO_4$
Exact Mass: 367.1031

Yield: 84% (510 mg, 1.39 mmol)
Appearance: colourless solid

^{1}H-NMR (400 MHz, $CDCl_3$) δ/ppm 7.59 (d, *J* = 7.8 Hz, 2H), 7.30-7.40 (m, 5H), 7.23-7.28 (m, 2H), 5.14 (s, 2H), 4.50 (br s, 1H), 4.01 (app t, *J* = 5.6 Hz, 2H), 2.62 (app t, *J* = 5.6 Hz, 2H). **^{13}C-NMR (100 MHz, $CDCl_3$)** δ/ppm 176.1 (C), 154.8 (C), 144.4 (C), 135.9 (C), 128.9 (q, *J* = 33 Hz, C), 128.5 (2CH), 128.2 (CH), 127.8 (2CH), 127.2 (2CH), 126.2 (q, *J* = 4 Hz, 2CH), 123.8 (q, *J* = 273 Hz, CF_3), 67.8 (CH_2), 46.0 (CH_2), 33.0 (CH_2). **^{19}F-NMR (376 MHz, $CDCl_3$)** δ/ppm -62.5 (s). **IR (neat)** ν/cm^{-1}: 3068 (broad), 2937 (w), 2906 (w), 1720 (m), 1692 (s), 1613 (m), 1398 (m), 1321 (m), 1301 (m), 1217 (s), 1198 (s), 1164 (s), 1113 (s), 1102 (s), 1064 (s), 1012 (m), 923 (m), 852 (m), 736 (m), 698 (m), 625 (m). **HR-MS** (TOF ES+) calcd for $C_{18}H_{17}F_3NO_4$ 368.1104, found 368.1104 (M+H$^+$).

Copies of NMR spectra

Benzyl (4-(trifluoromethoxy)phenyl)carbamate (3b): ^{1}H, ^{13}C, ^{19}F, HSQC

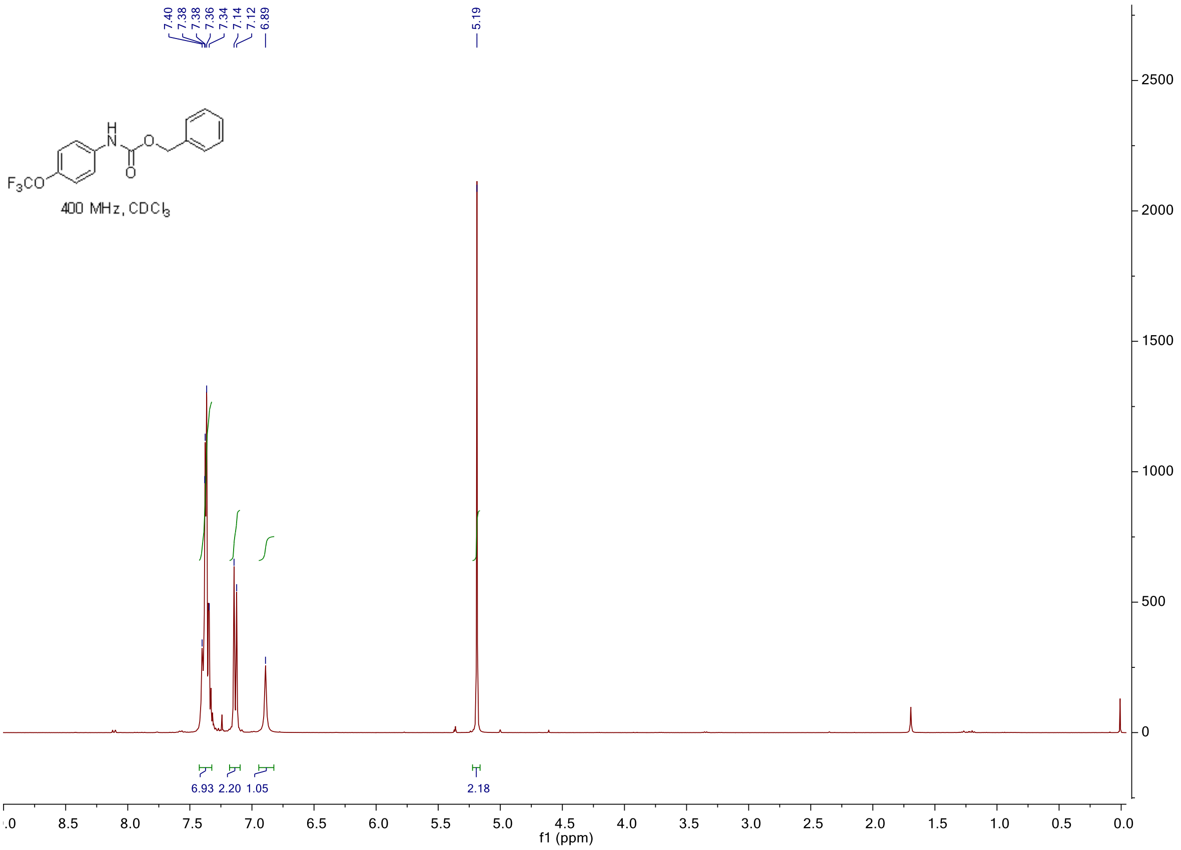

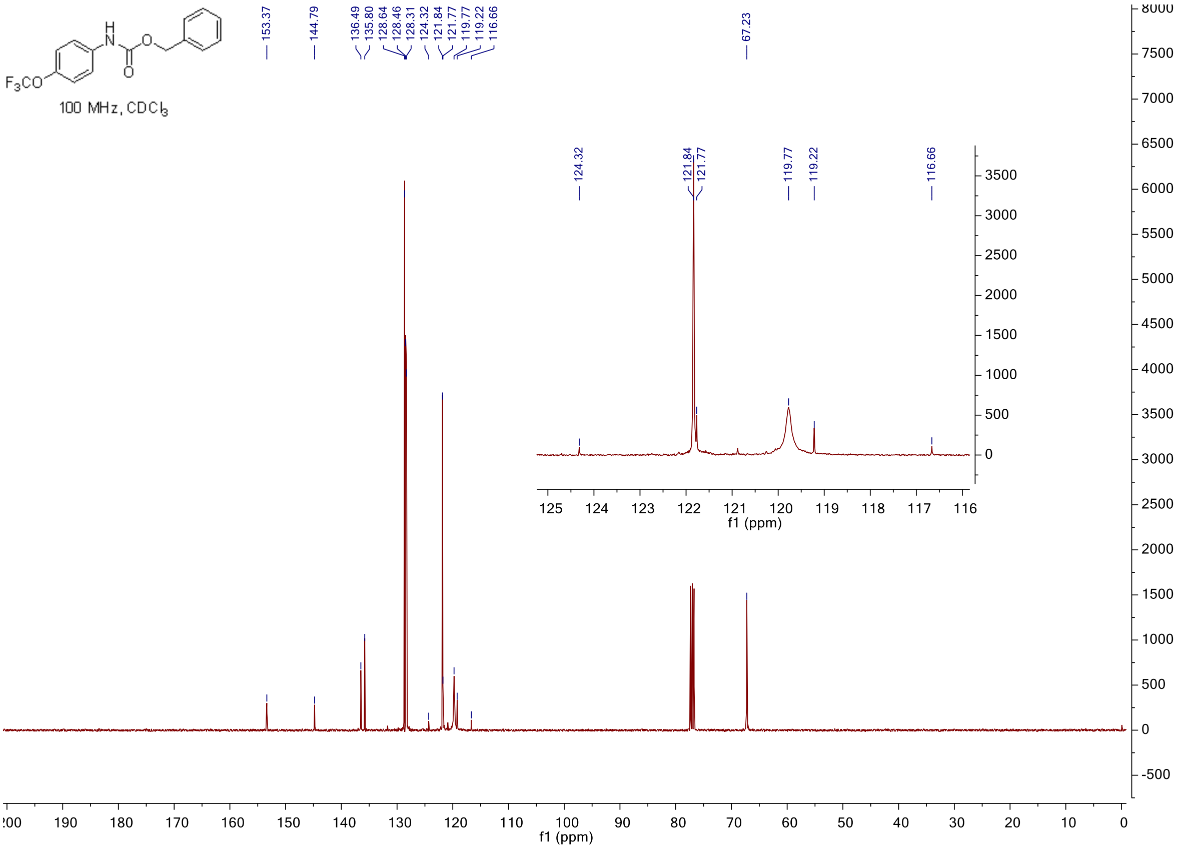

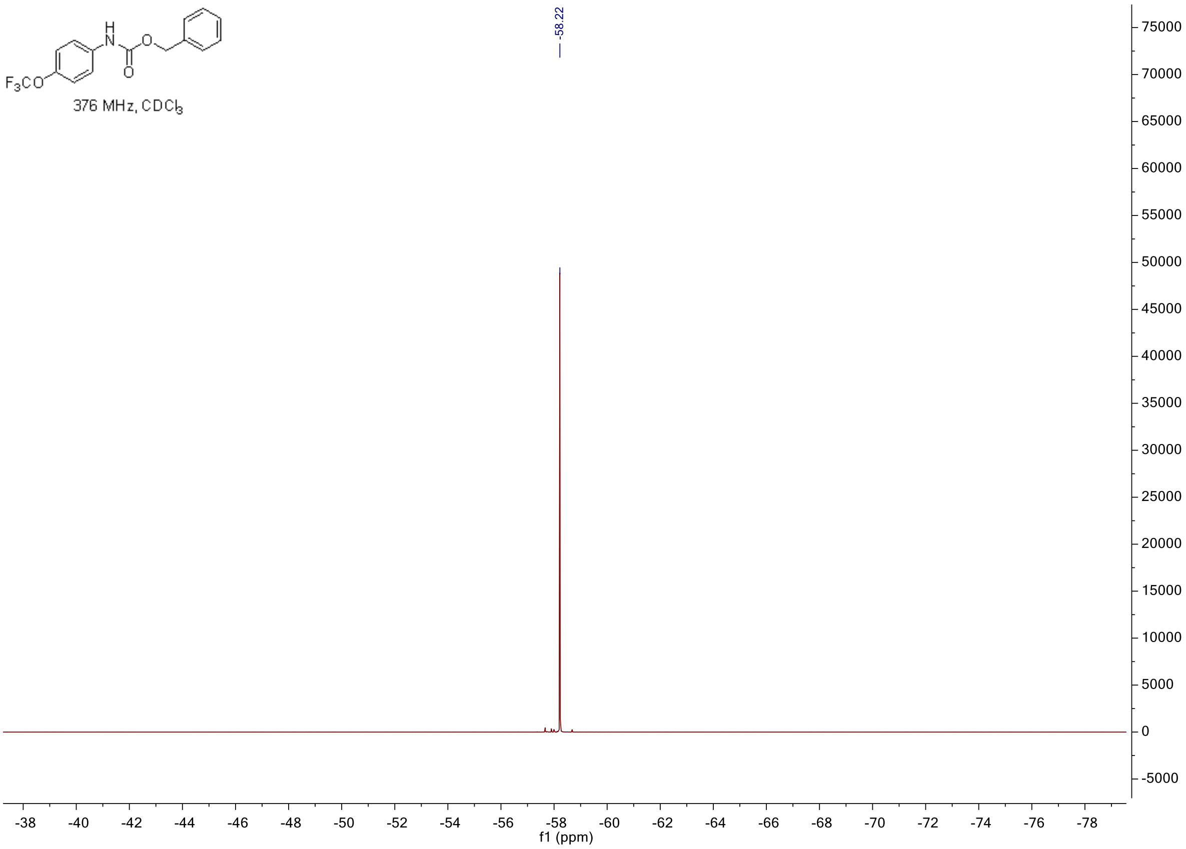

F3CO
376 MHz, CDCl3
-58.22
f1 (ppm)

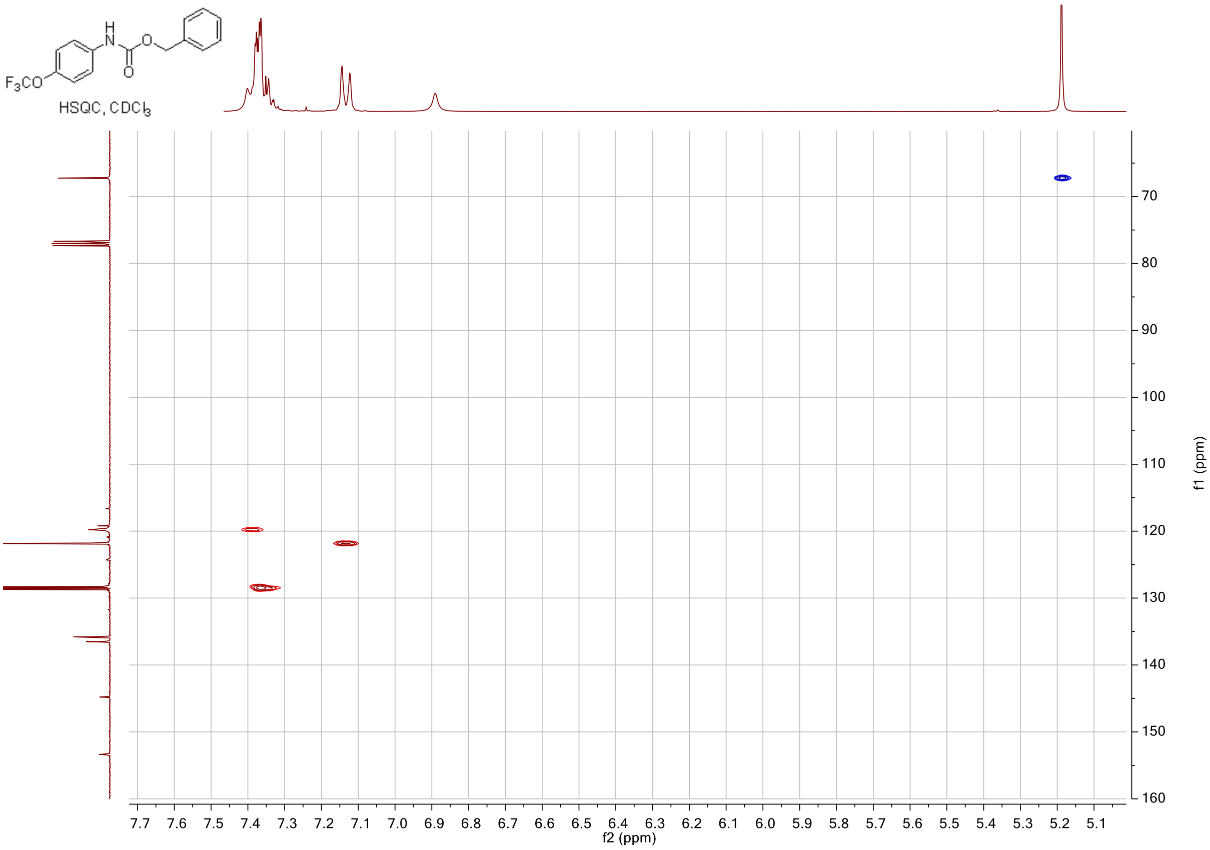

F3CO
HSQC, CDCl3
f2 (ppm)
f1 (ppm)

Benzyl (2-bromo-5-fluorophenyl)carbamate (3c): ^{1}H, ^{13}C, ^{19}F, HSQC

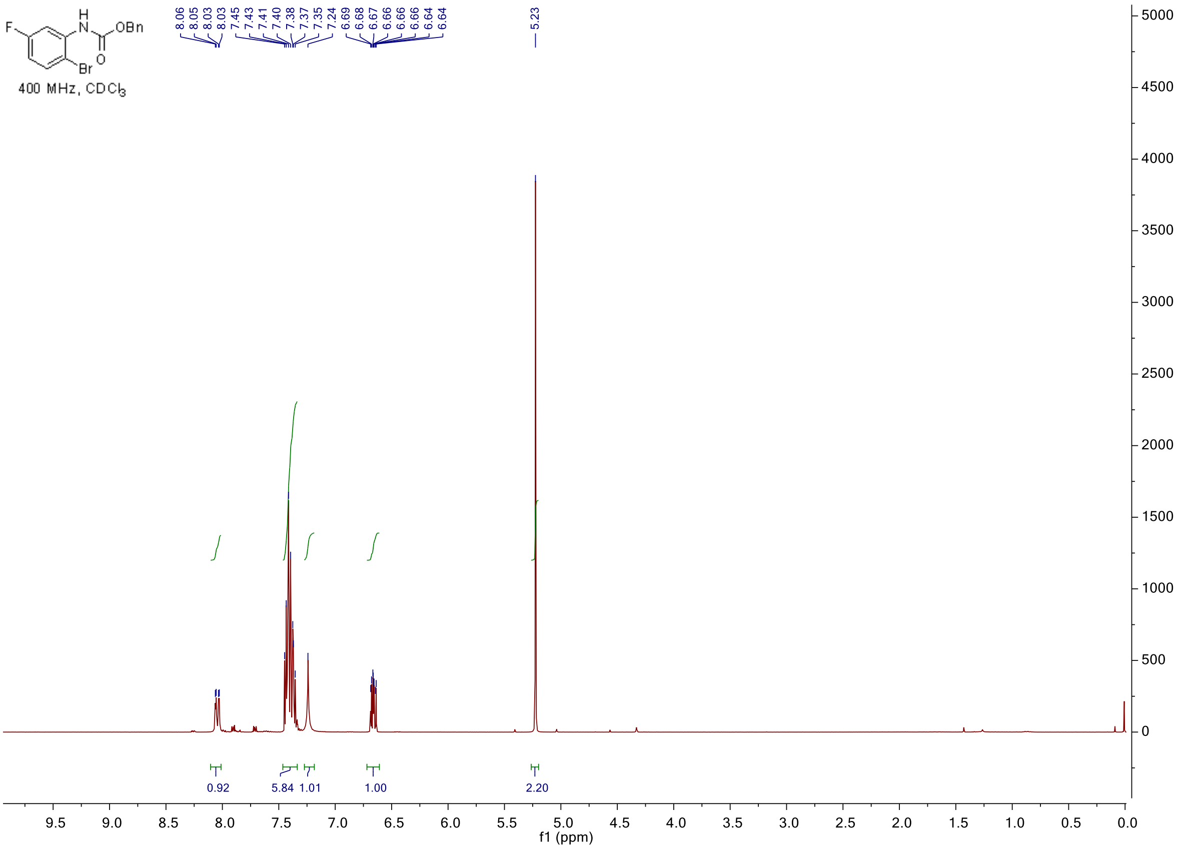

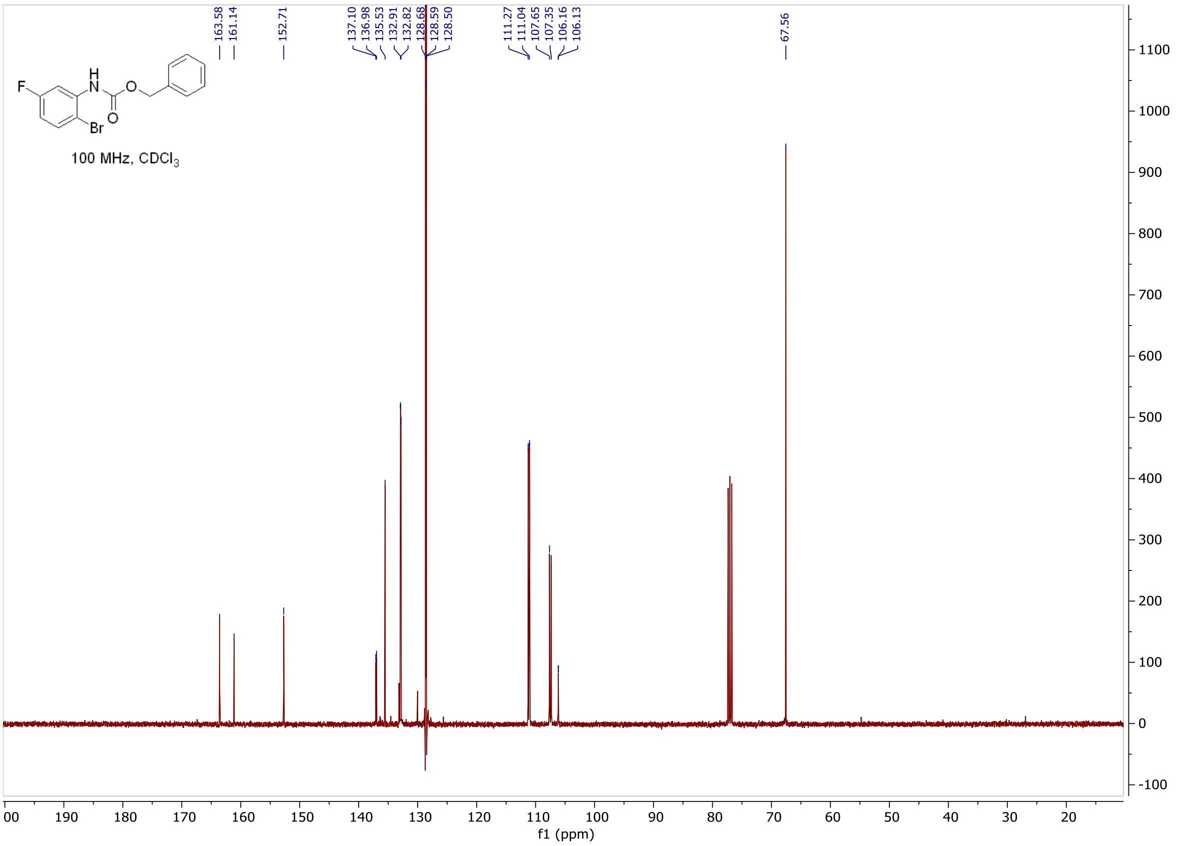

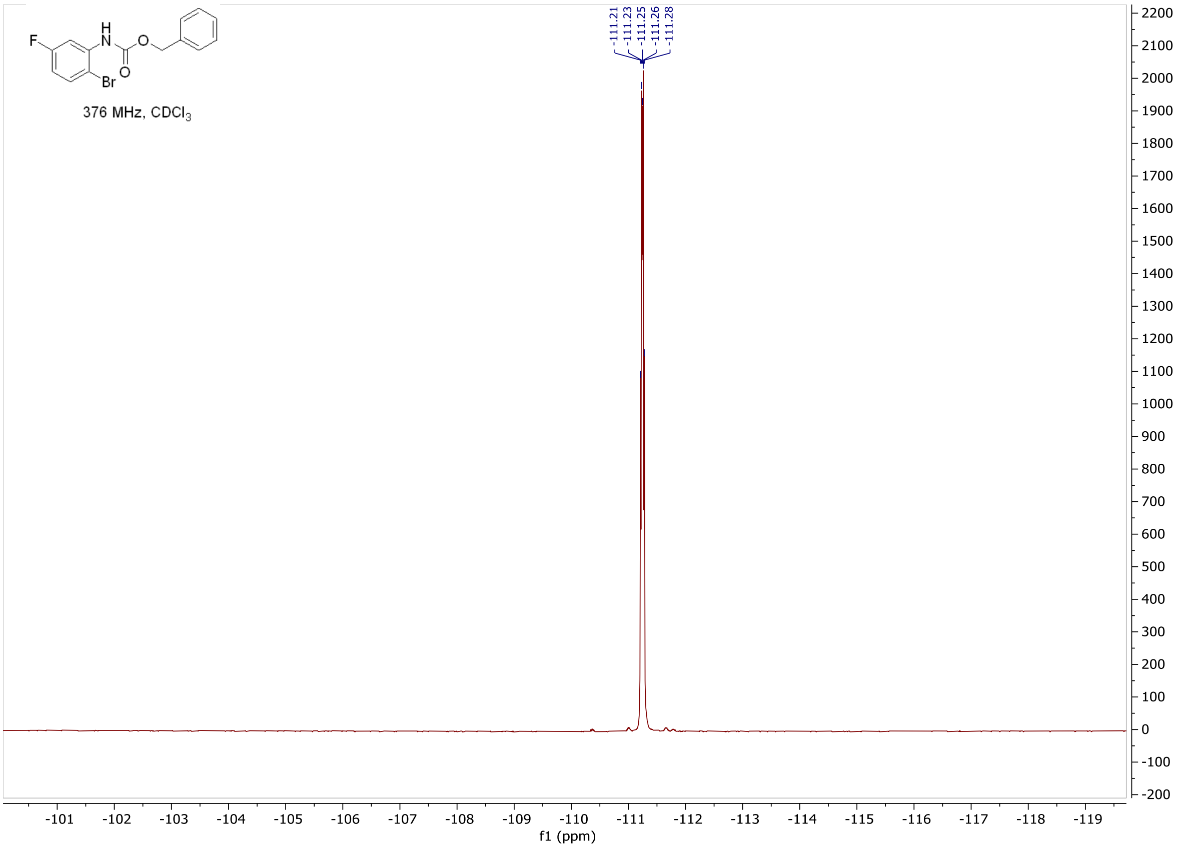

F
H
N
O
O
Br
376 MHz, CDCl3
-111.21
-111.23
-111.25
-111.26
-111.28
f1 (ppm)

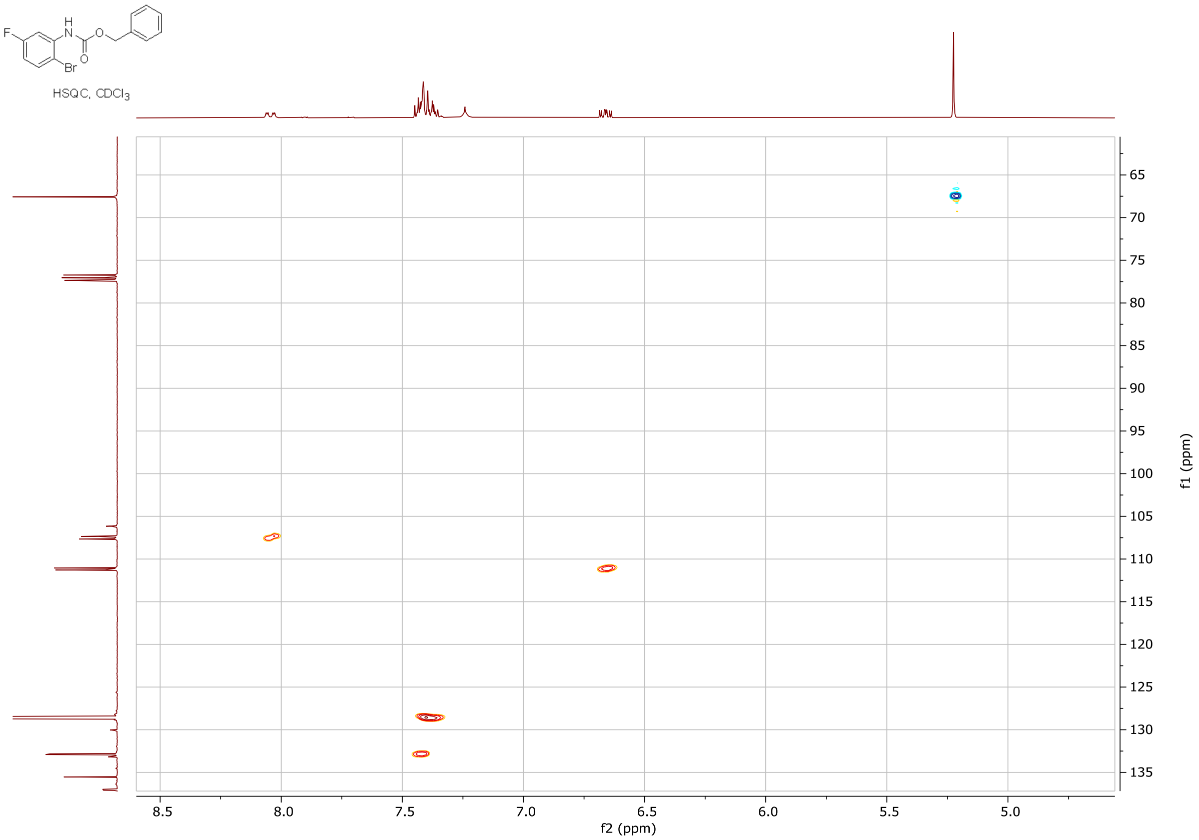

F
H
N
O
O
Br
HSQC, CDCl3
f2 (ppm)
f1 (ppm)

Benzyl (2-fluorophenyl)carbamate (3d): ^{1}H, ^{13}C, ^{19}F, HSQC

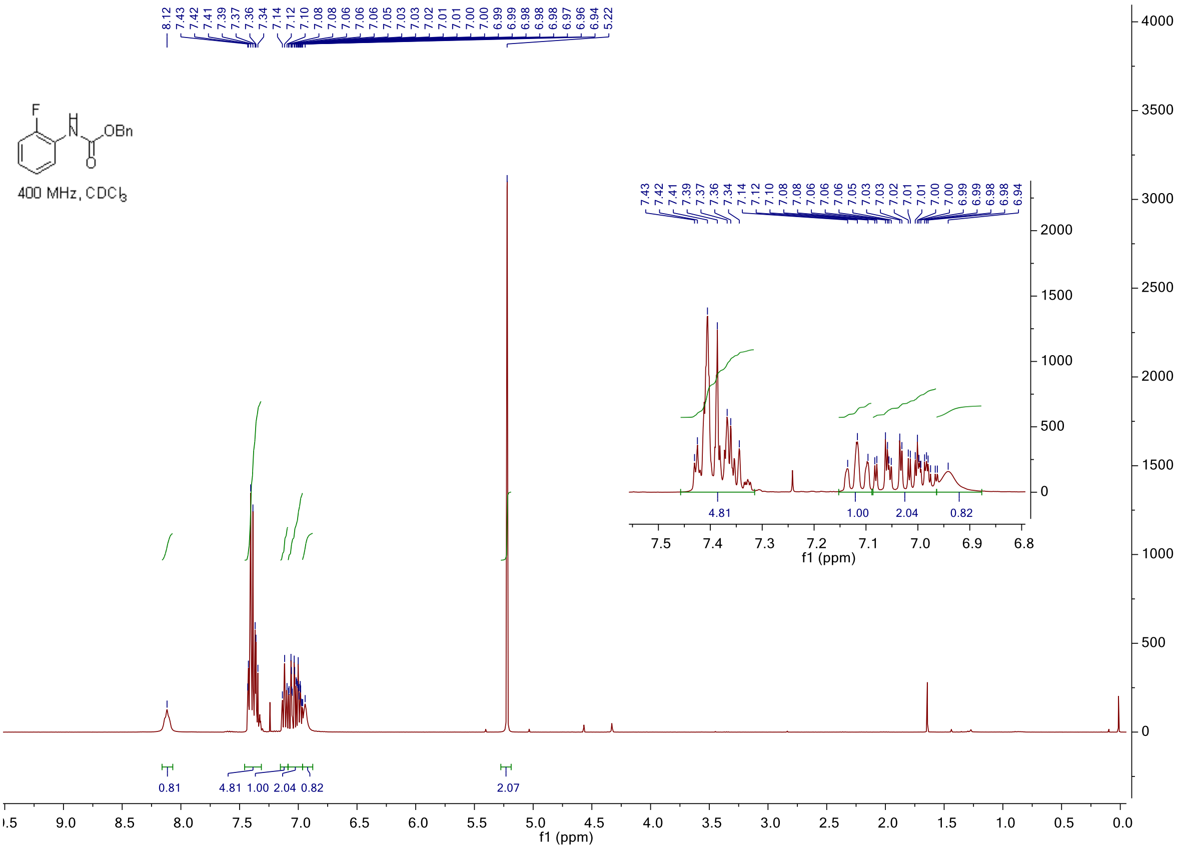

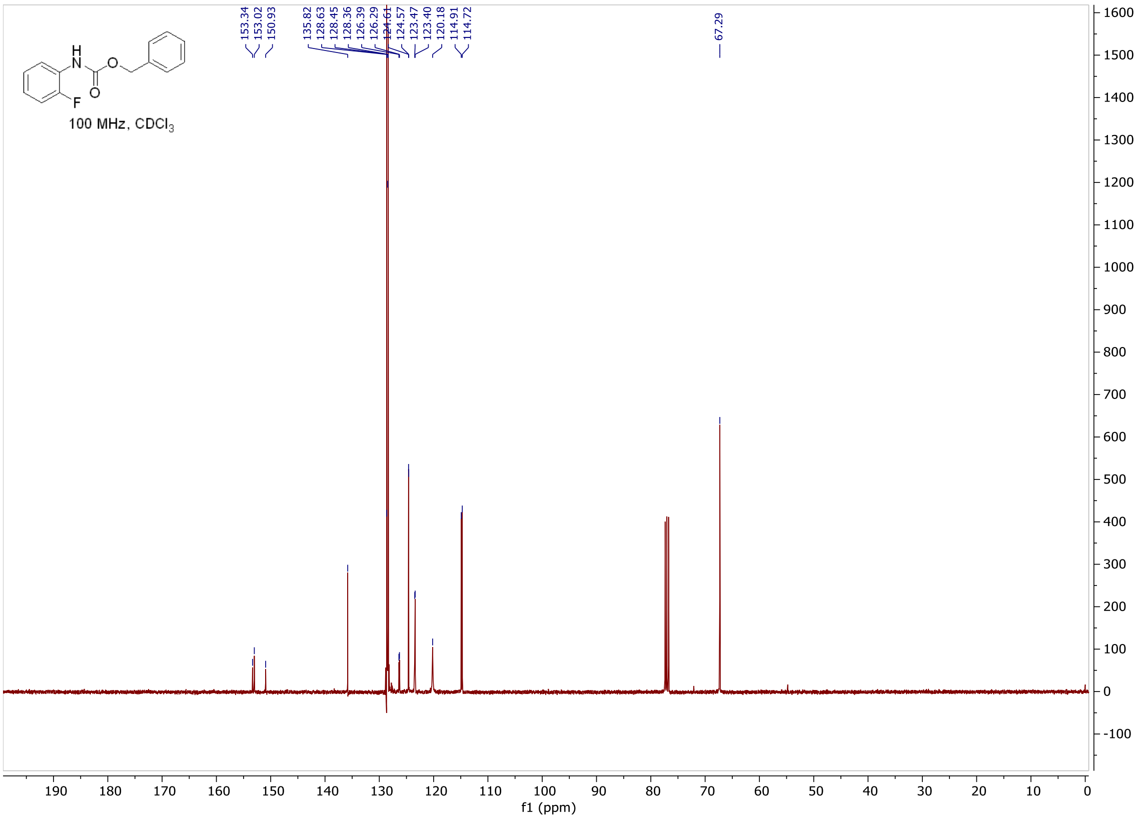

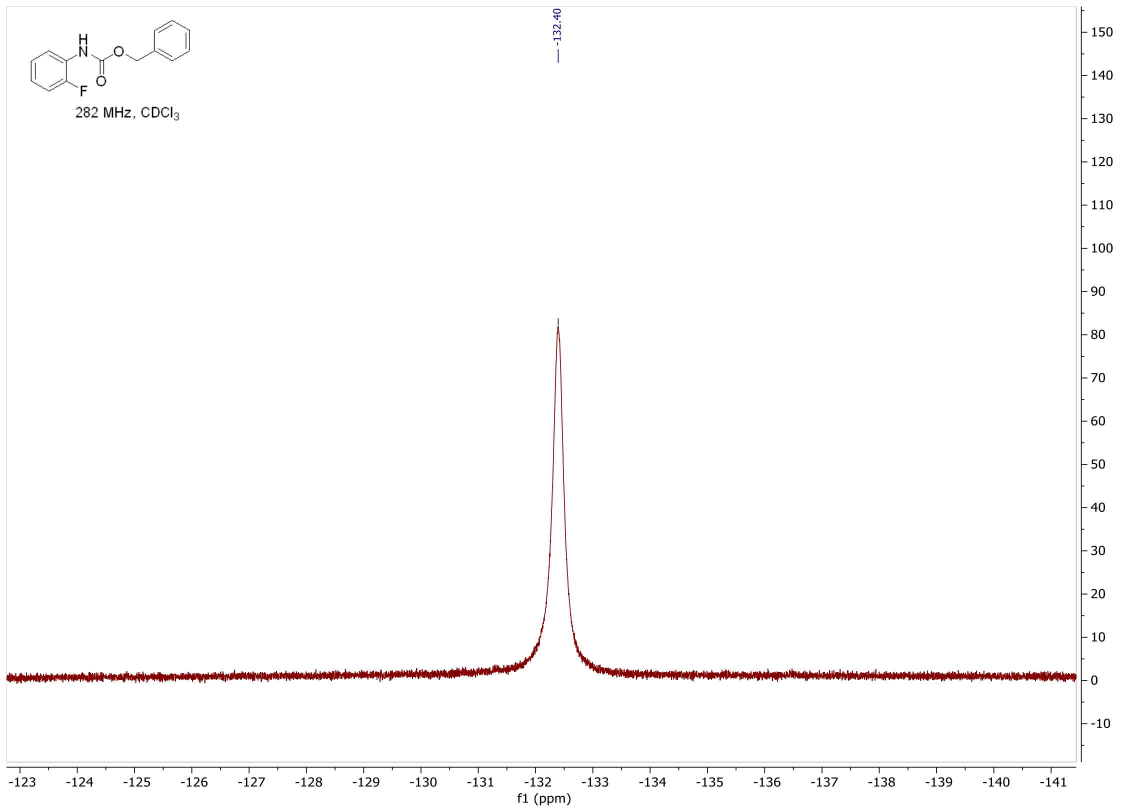

282 MHz, CDCl3
-132.40
f1 (ppm)

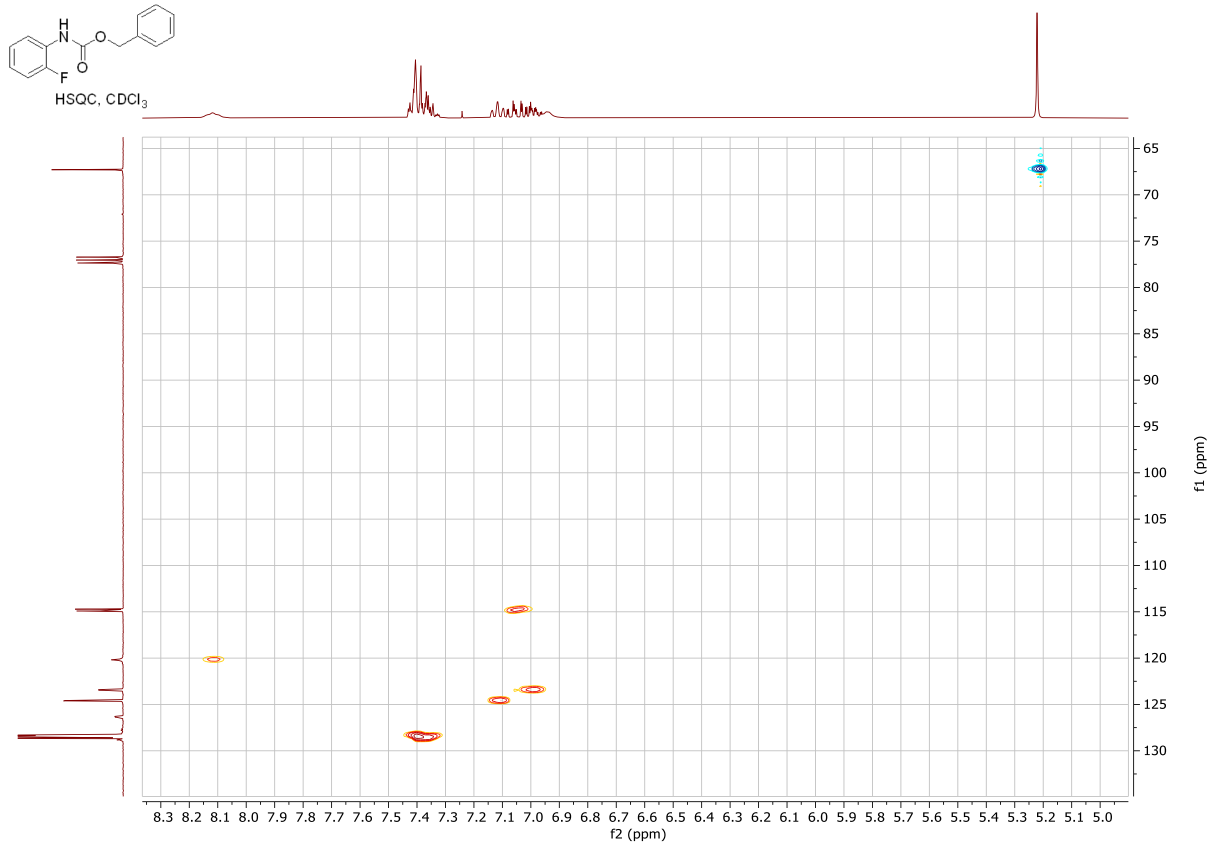

HSQC, CDCl3
f2 (ppm)
f1 (ppm)

Benzyl (4-chloro-3-methylphenyl)carbamate (3e): ^{1}H, ^{13}C, HSQC

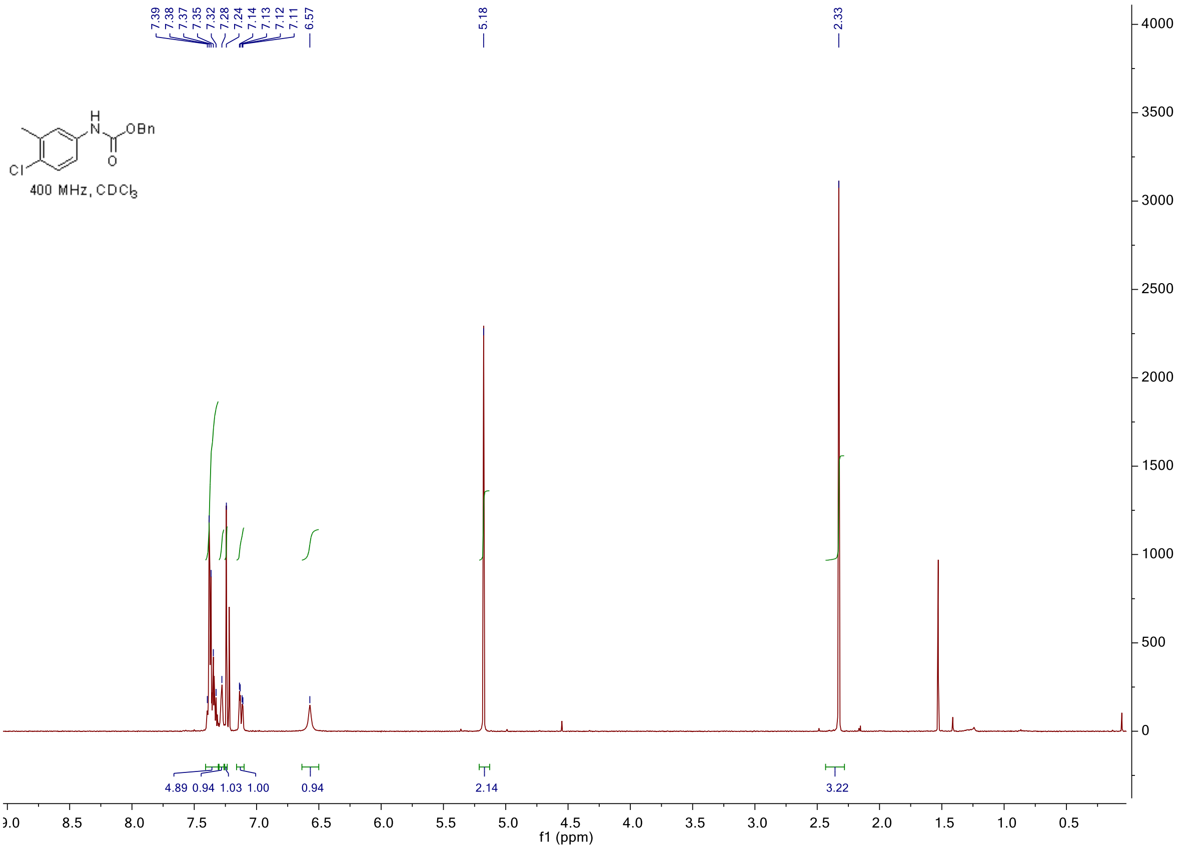

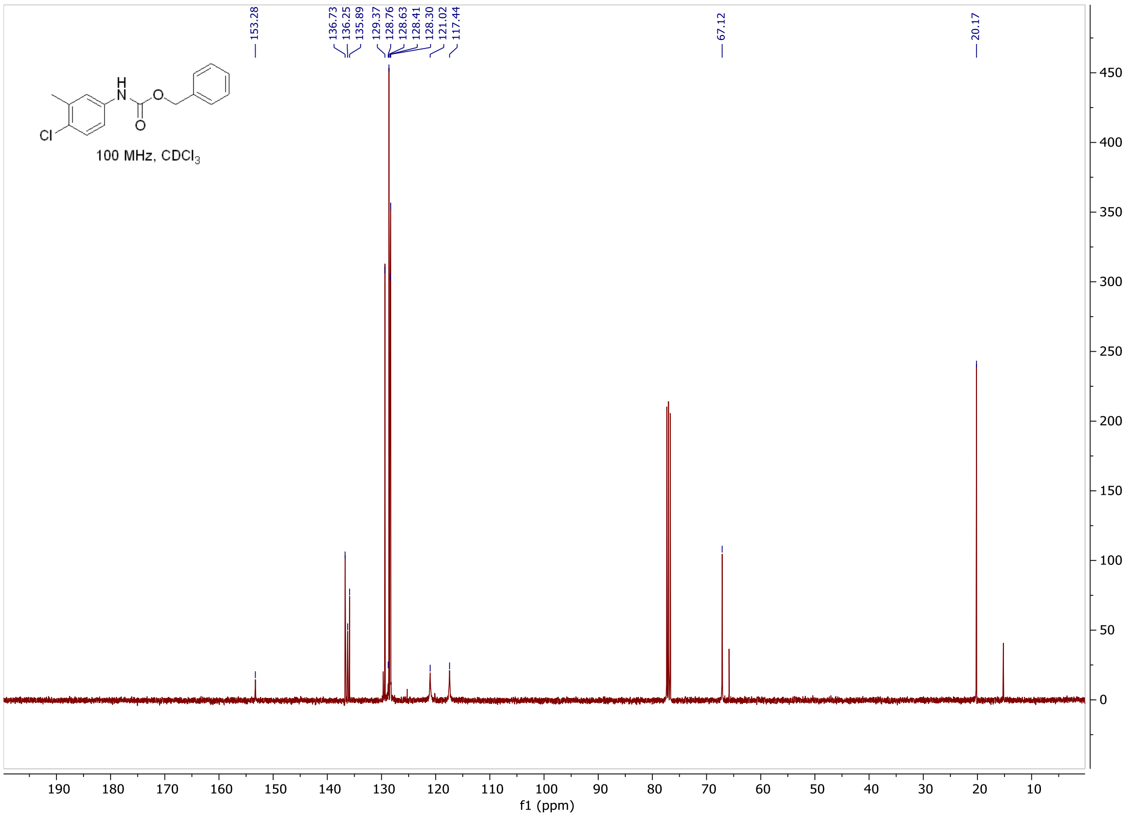

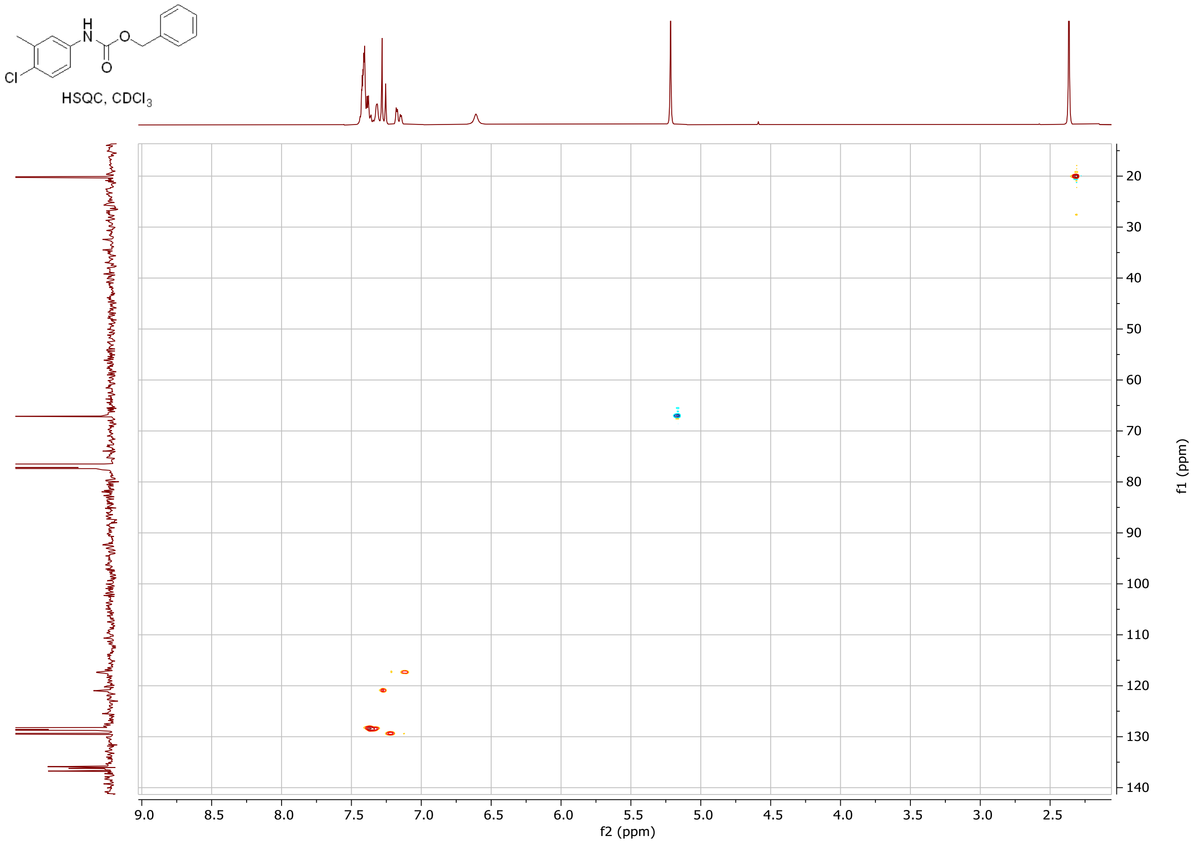
HSQC, CDCl3
f2 (ppm)
f1 (ppm)

Benzyl (2-nitrobenzyl)carbamate (3f): ^{1}H, ^{13}C, HSQC

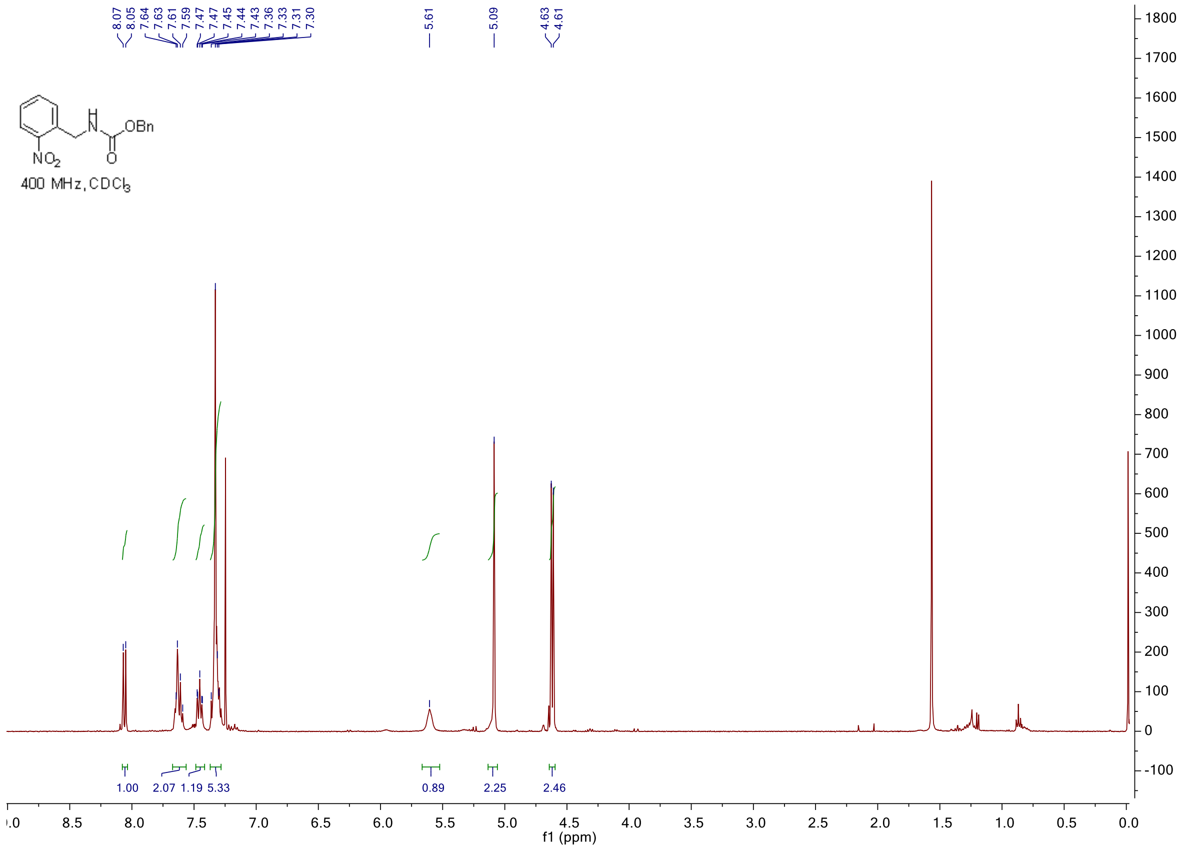

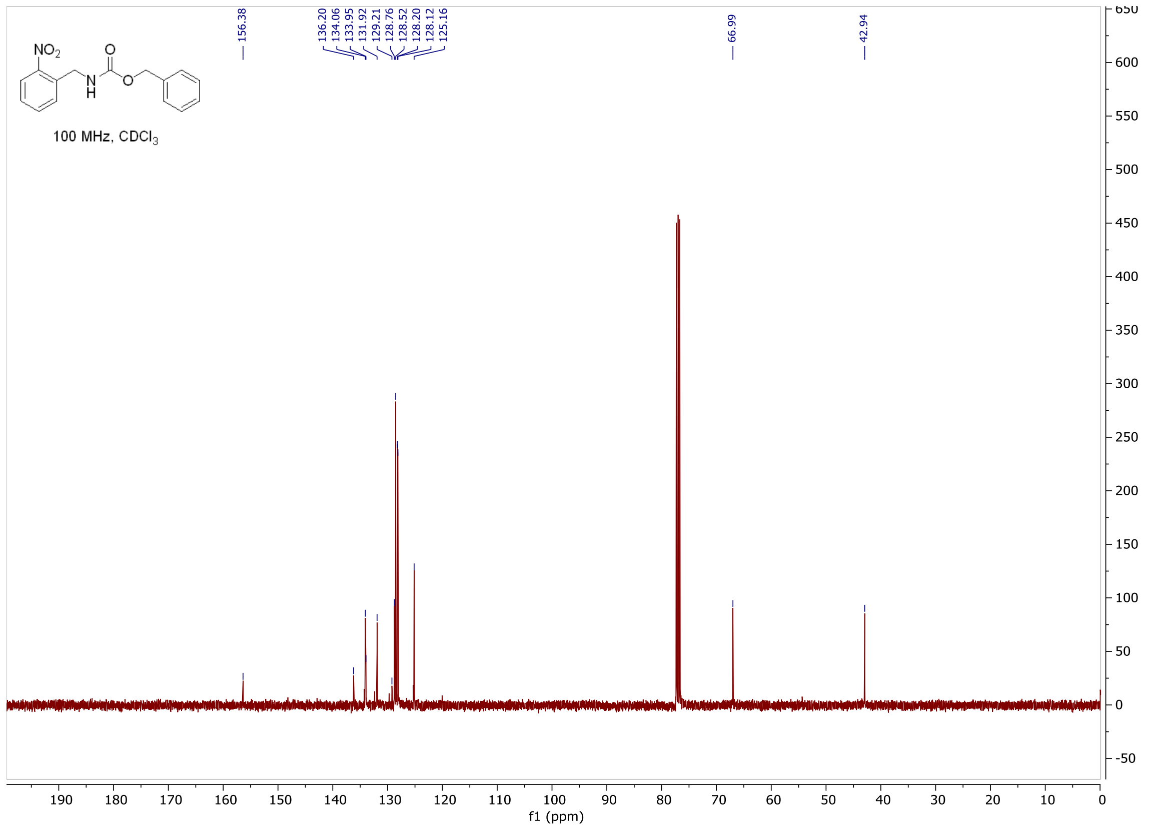

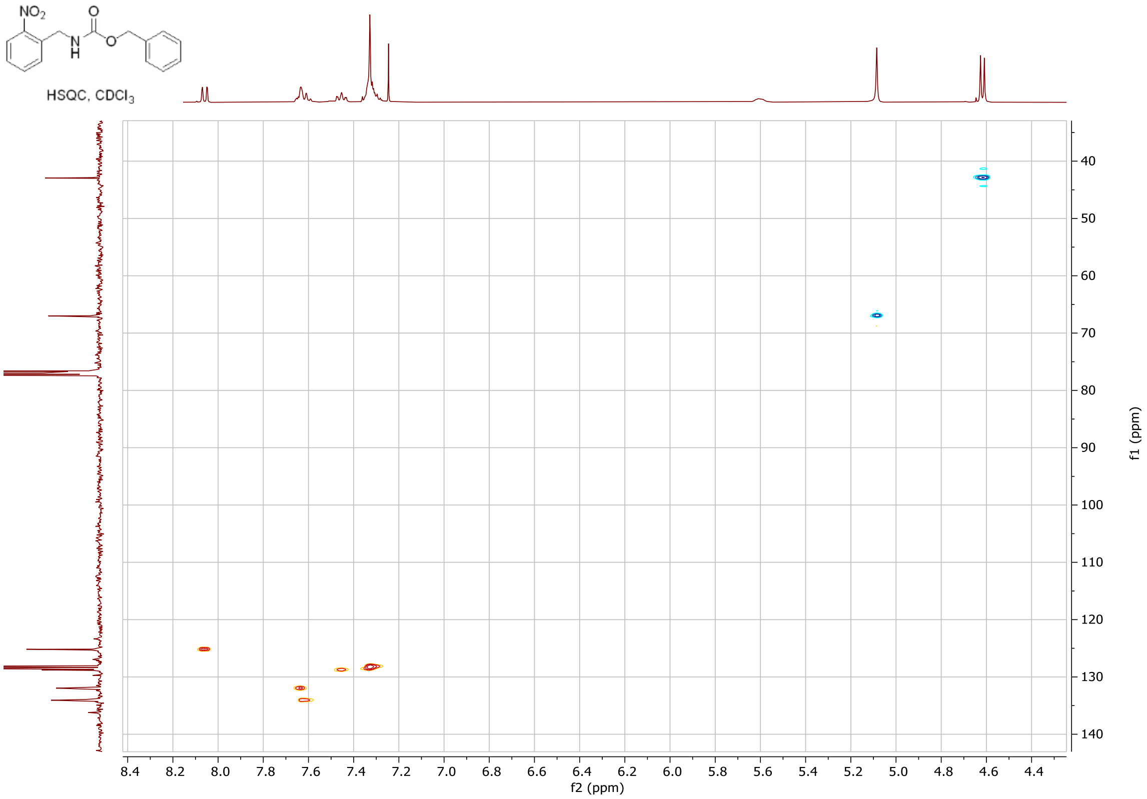
NO2
O
N
H
O
HSQC, CDCl3
40
50
60
70
80
90
100
110
120
130
140
f1 (ppm)
8.4 8.2 8.0 7.8 7.6 7.4 7.2 7.0 6.8 6.6 6.4 6.2 6.0 5.8 5.6 5.4 5.2 5.0 4.8 4.6 4.4
f2 (ppm)

Benzyl (3,4-dimethoxybenzyl)carbamate (3g): ^{1}H, ^{13}C, HSQC

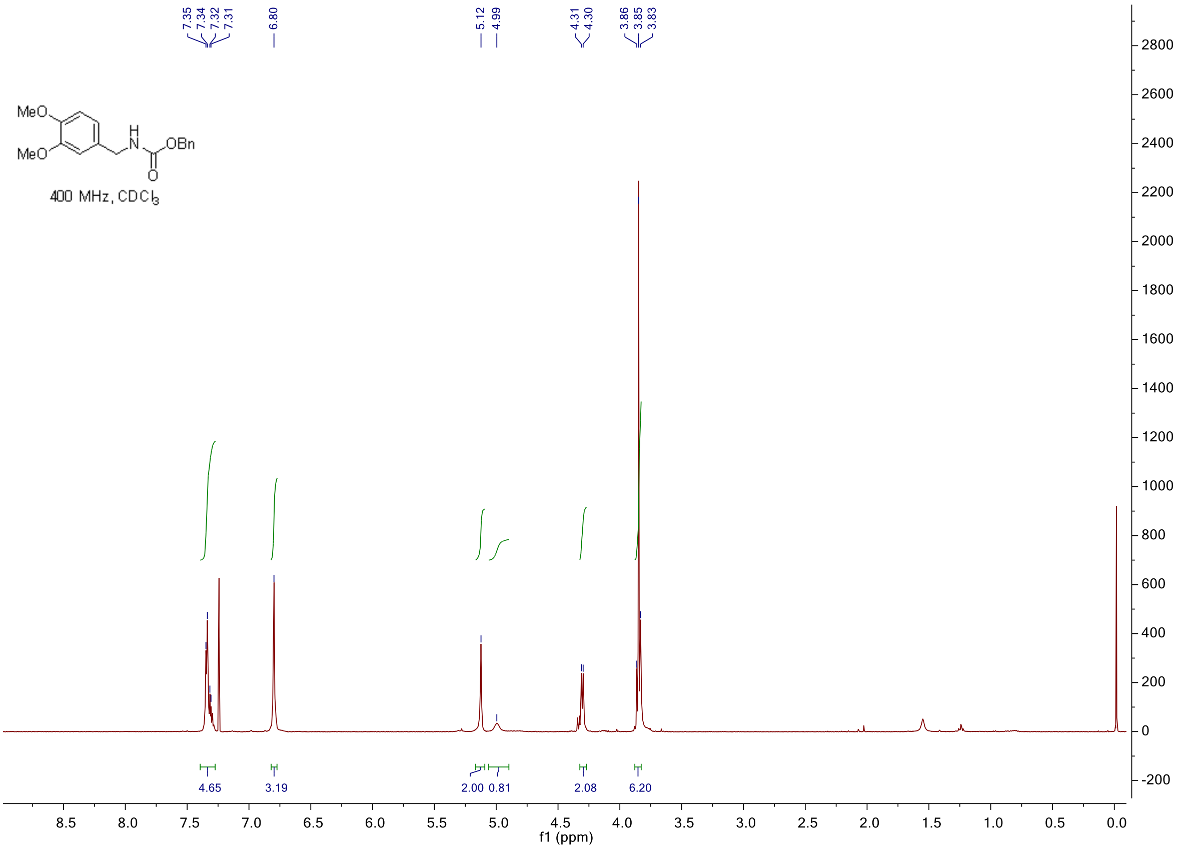

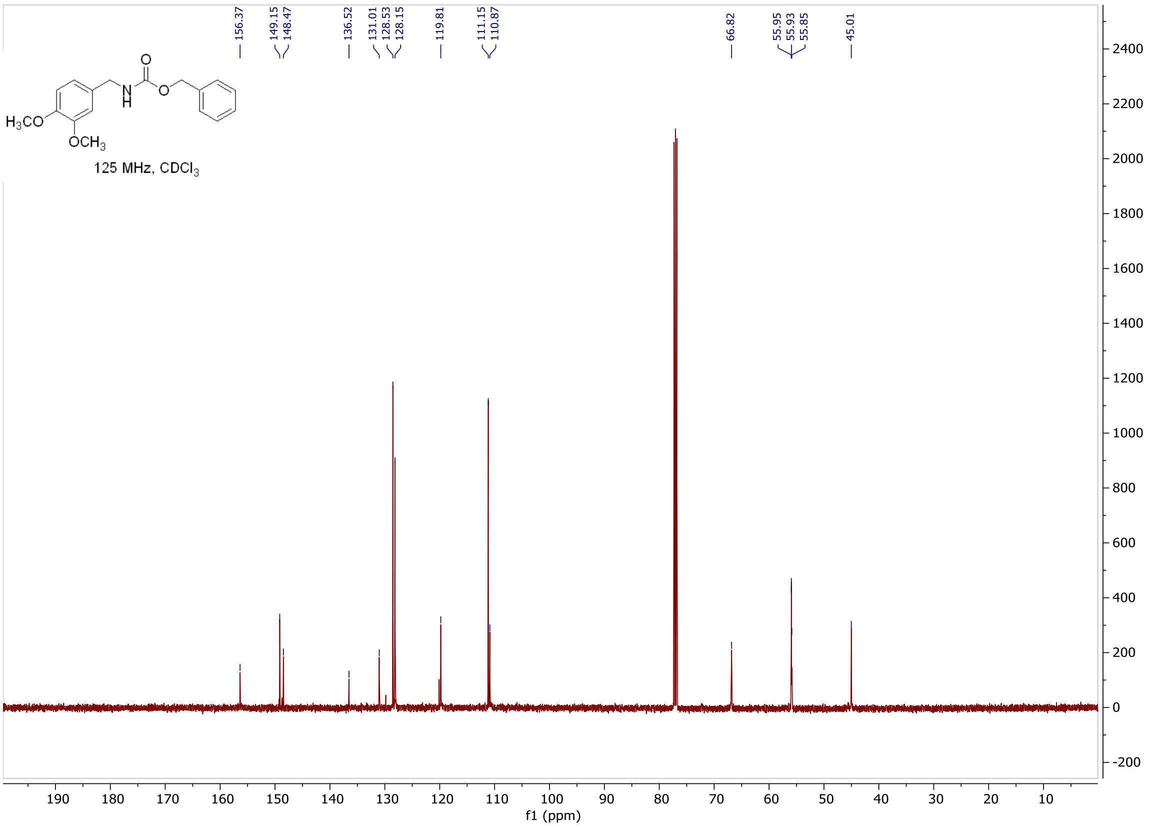

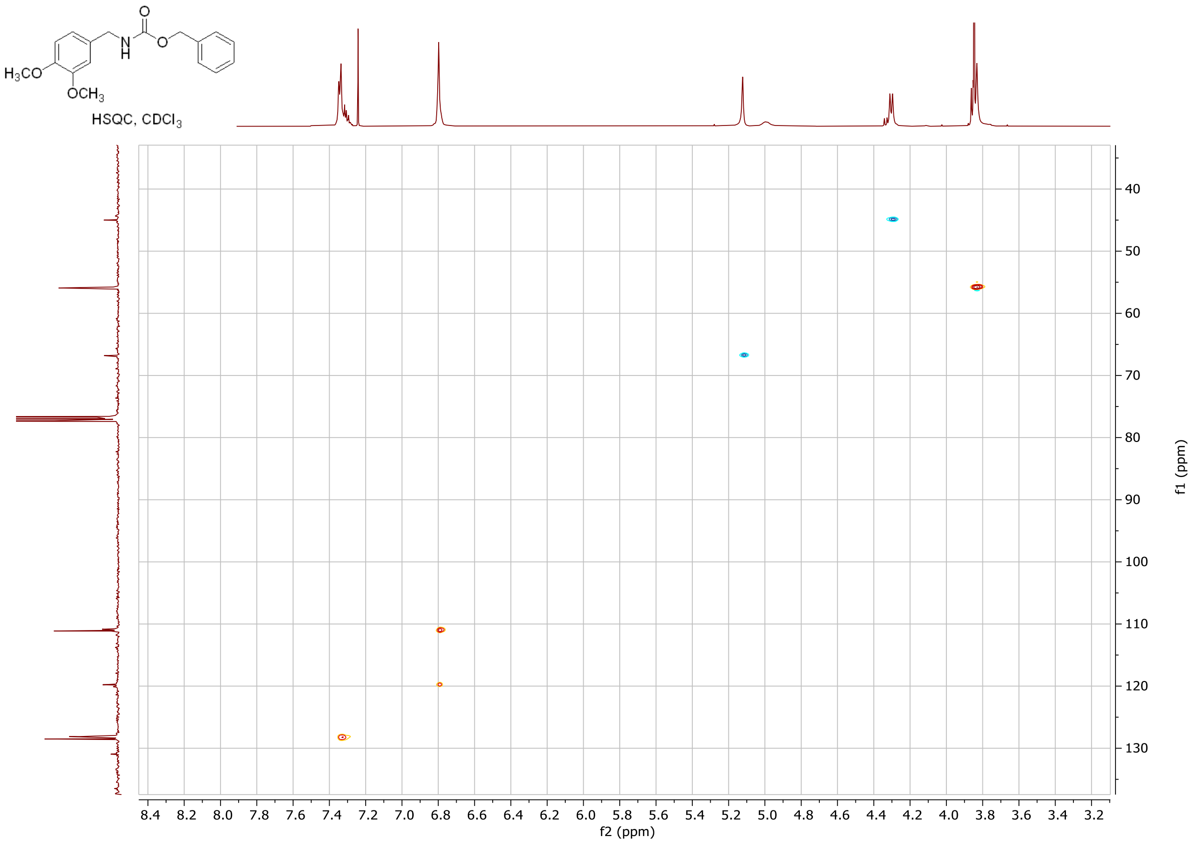
H3CO
OCH3
HSQC, CDCl3
f1 (ppm)
f2 (ppm)

Benzyl (4-chlorobenzyl)carbamate (3h): ^{1}H, ^{13}C, HSQC

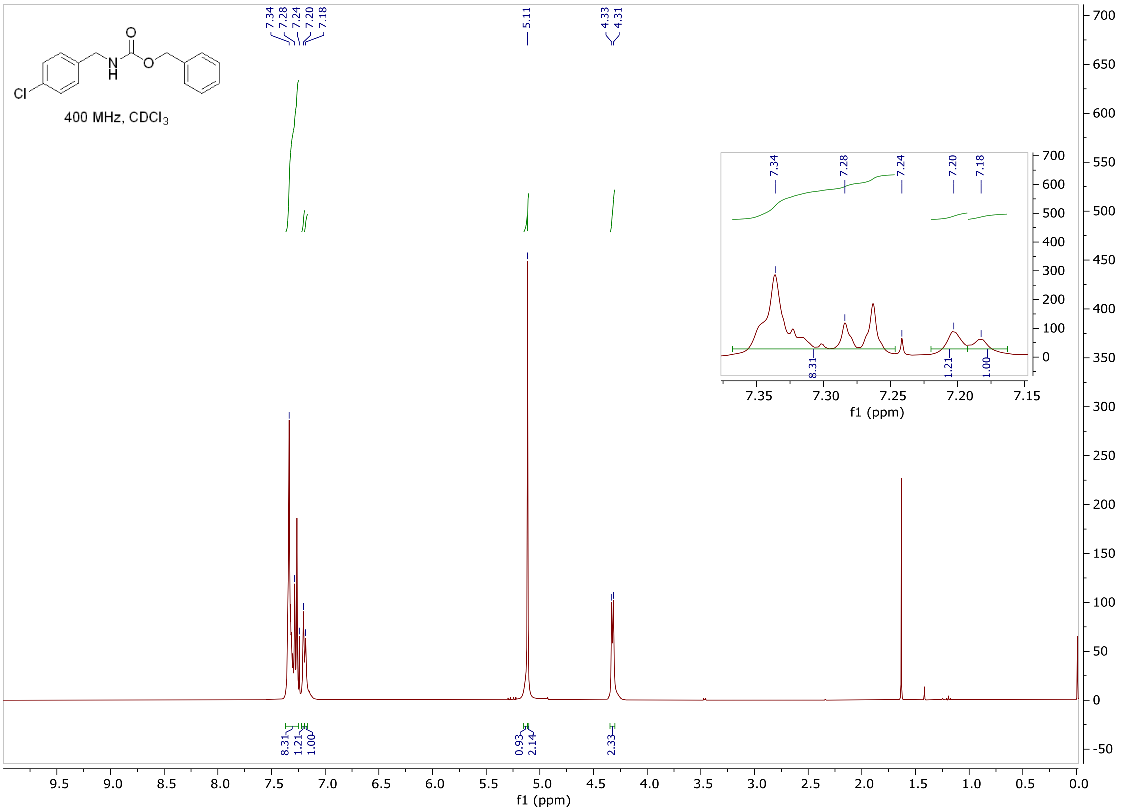

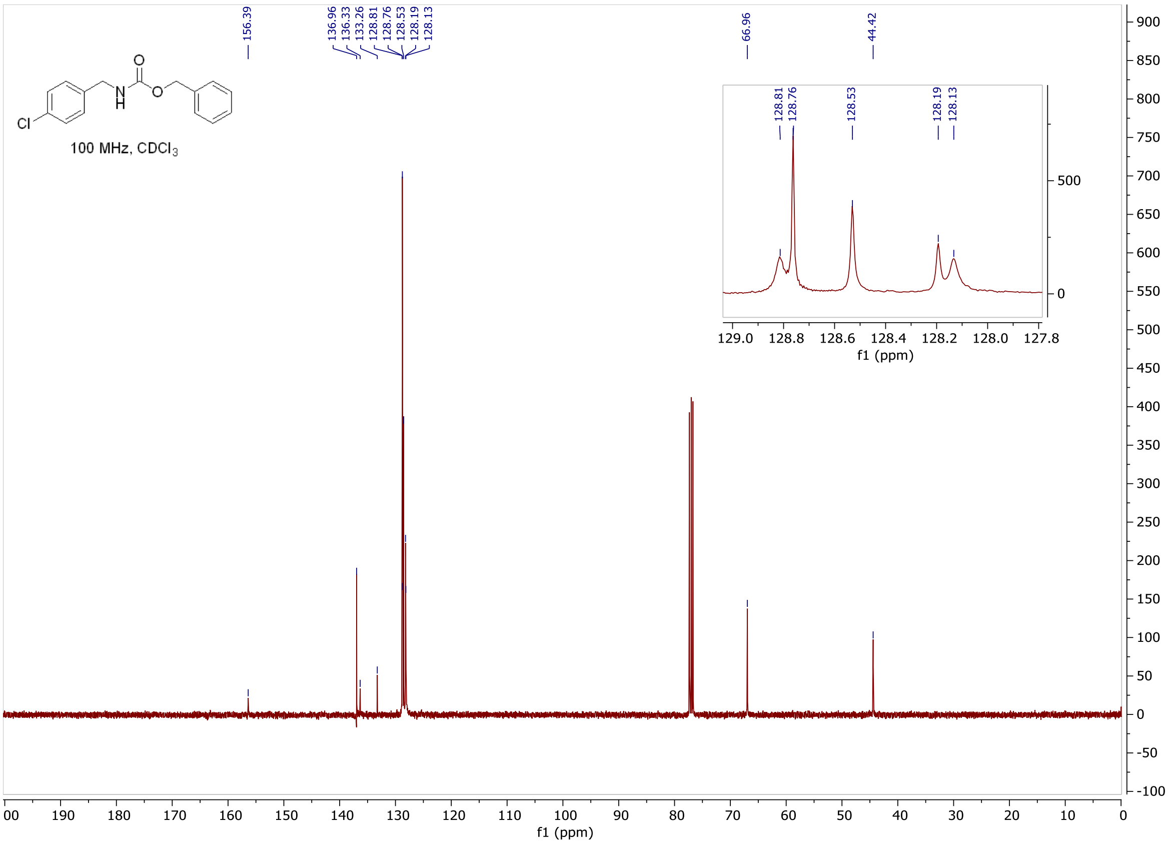

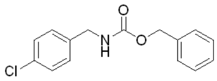

HSQC, $CDCl_3$

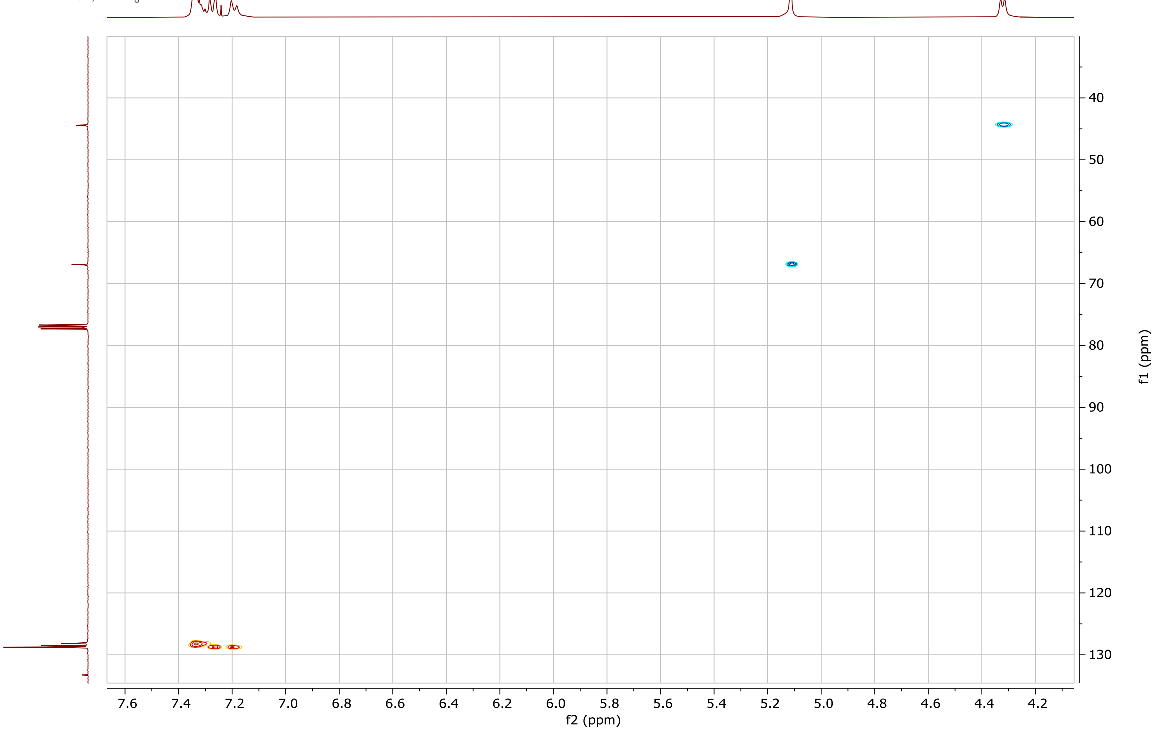

Benzyl (1-(4-isobutylphenyl)ethyl)carbamate (3i): ^{1}H, ^{13}C, HSQC

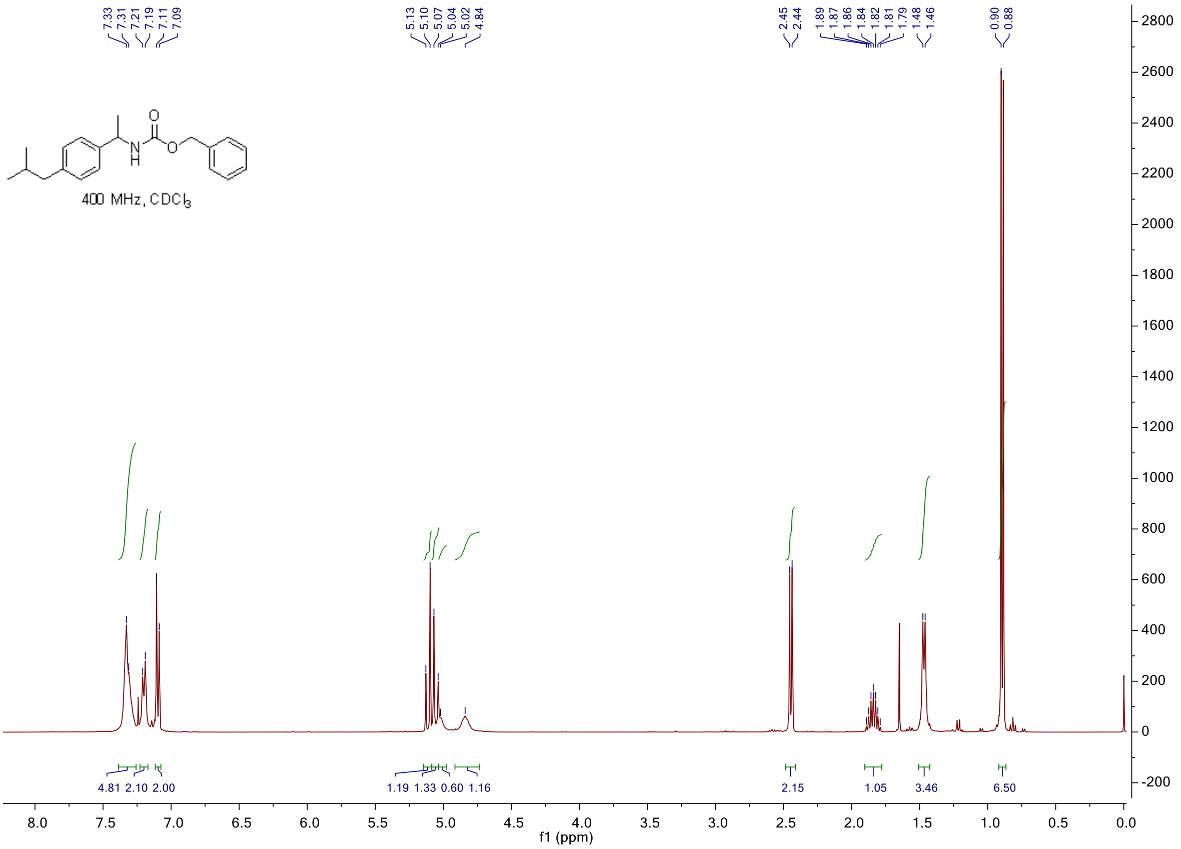

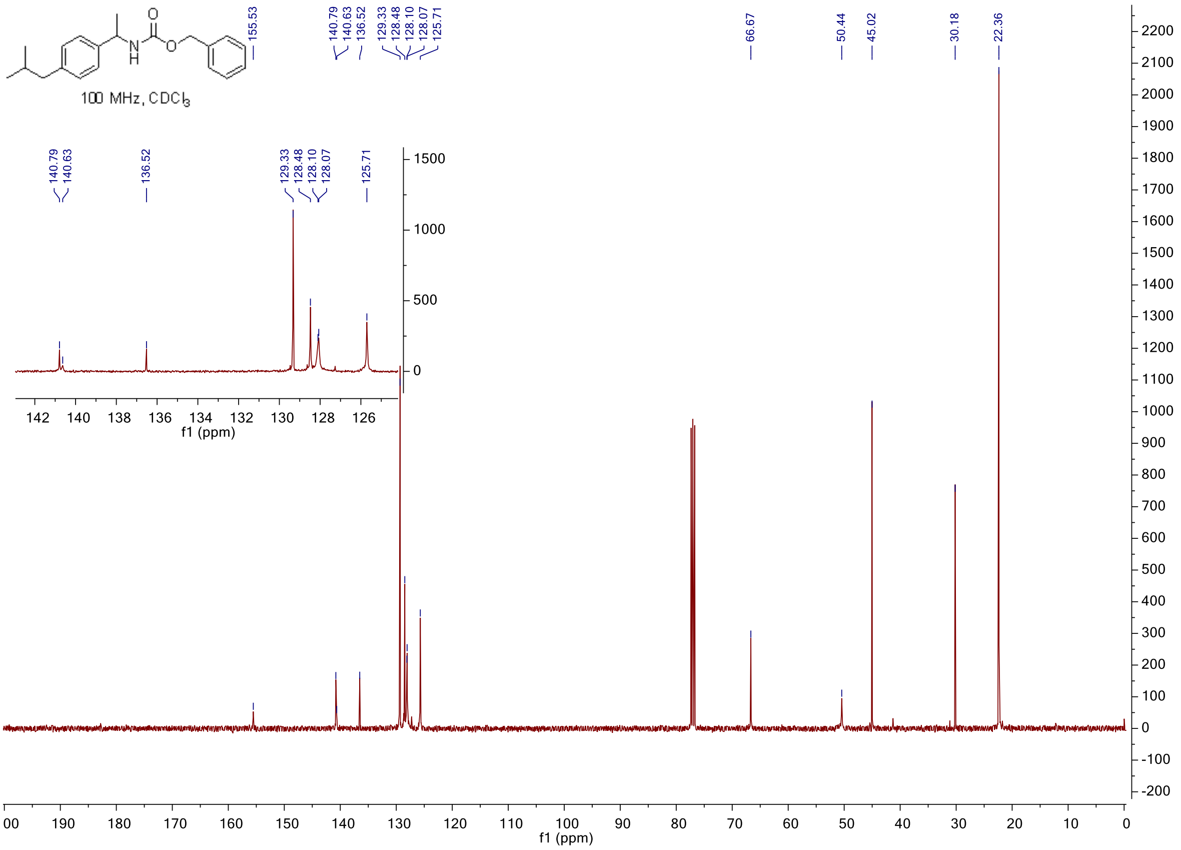

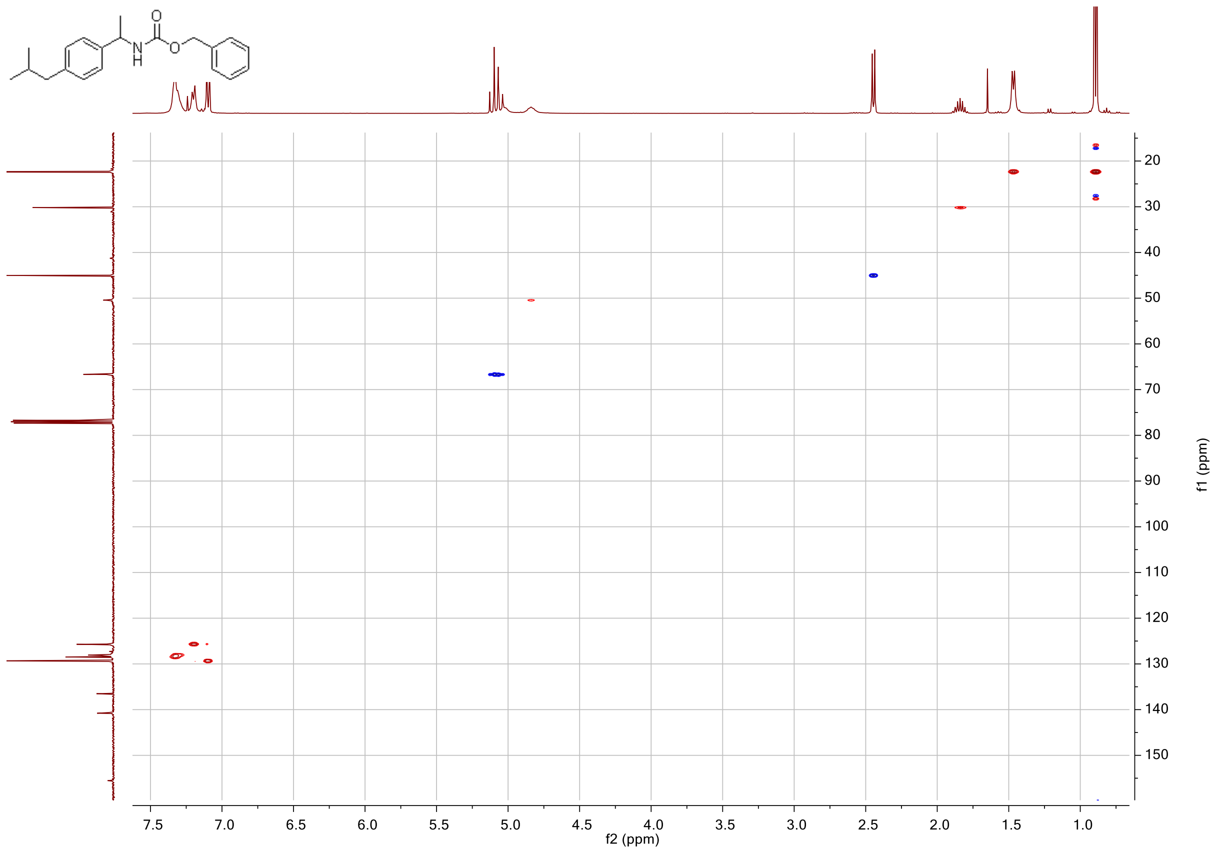

O
N
H
O
f1 (ppm)
f2 (ppm)

Benzyl cyclohexylcarbamate (3j): ^{1}H, ^{13}C, HSQC

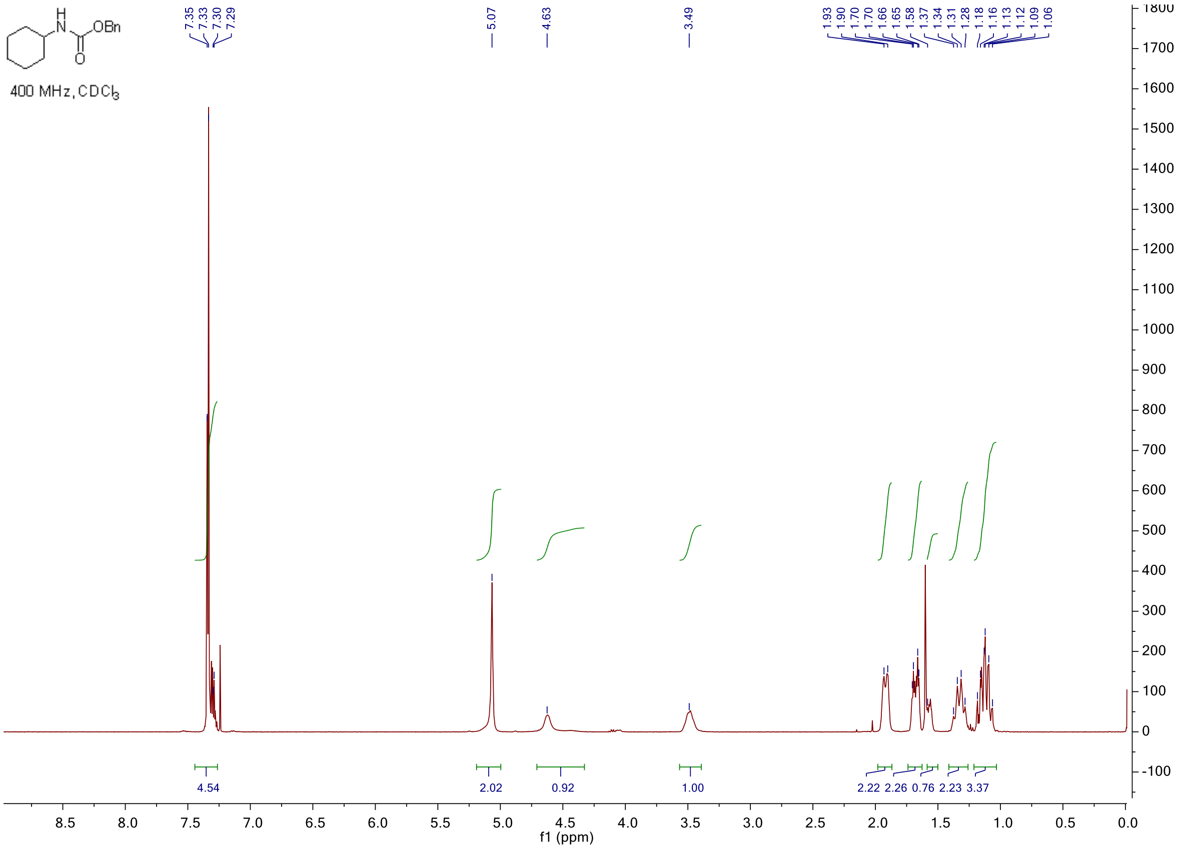

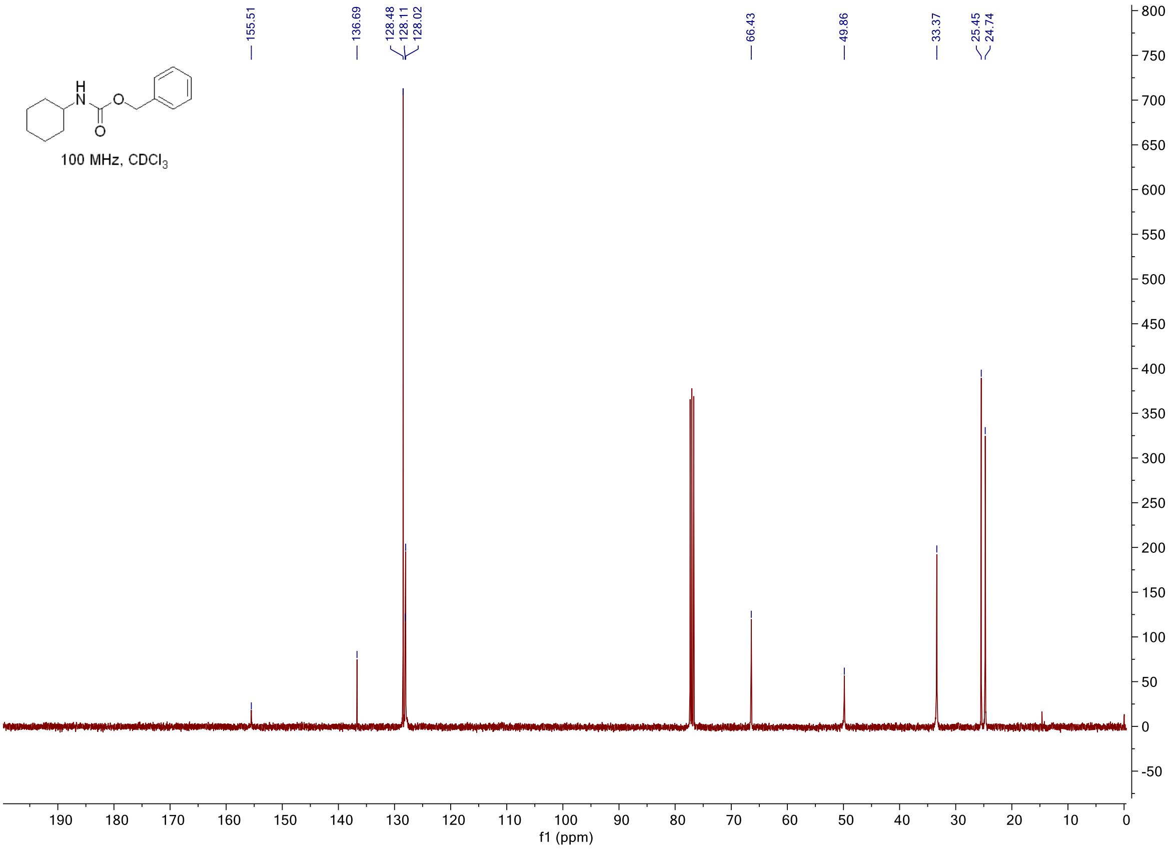

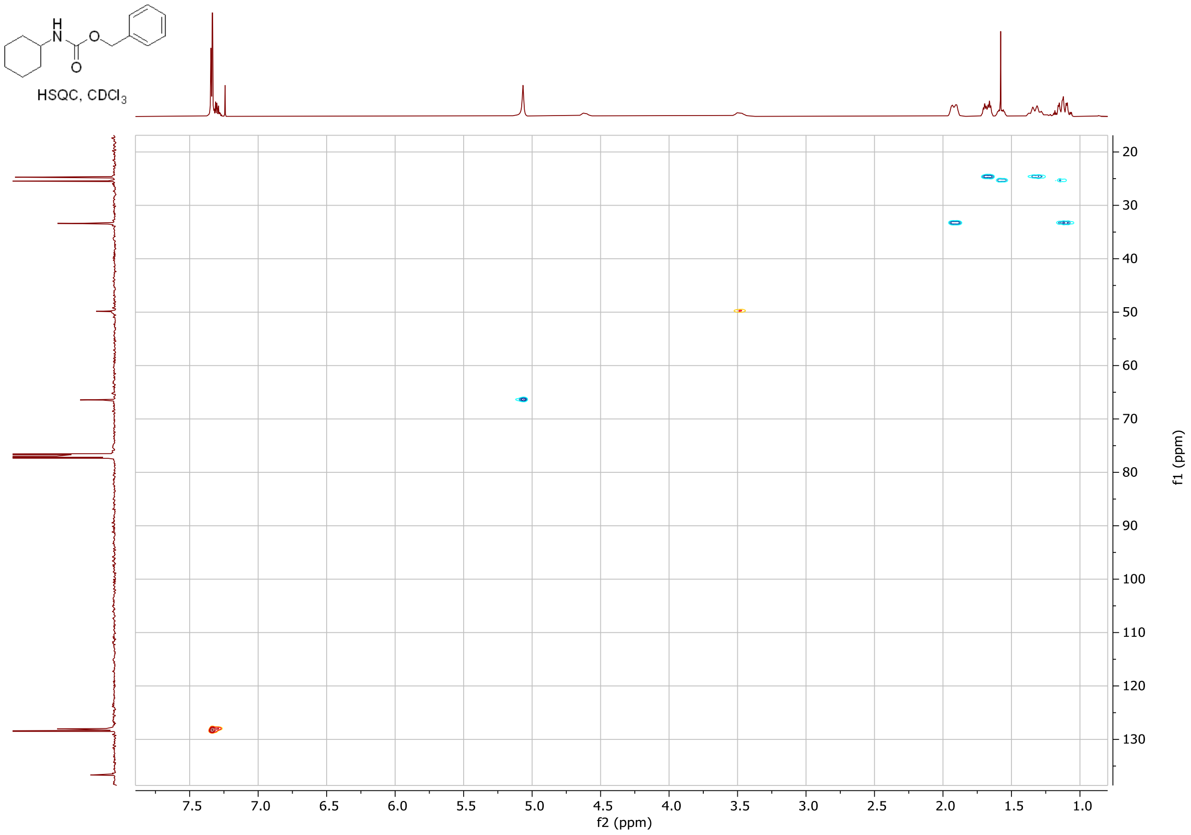
HSQC, CDCl3
f2 (ppm)
f1 (ppm)

Benzyl (*E*)-but-2-en-2-ylcarbamate (3l): ^{1}H, ^{13}C, HSQC, NOESY

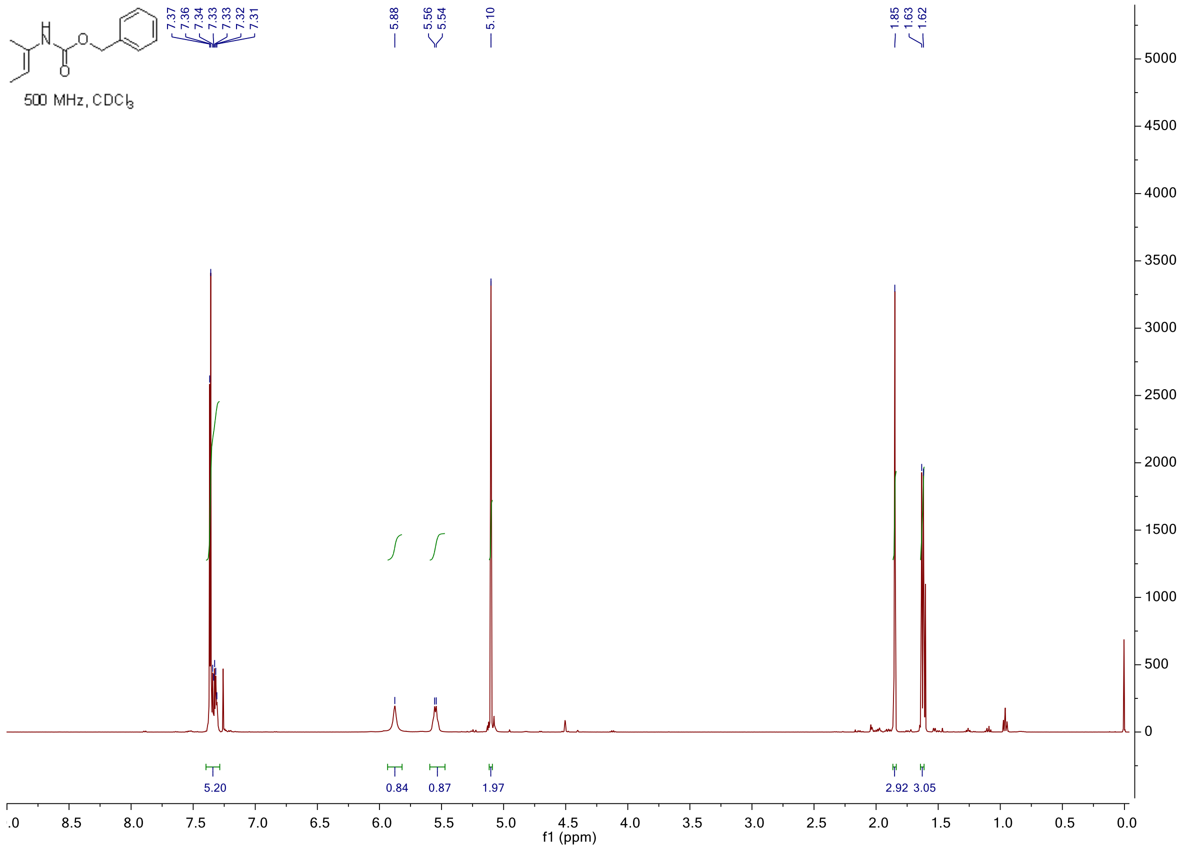

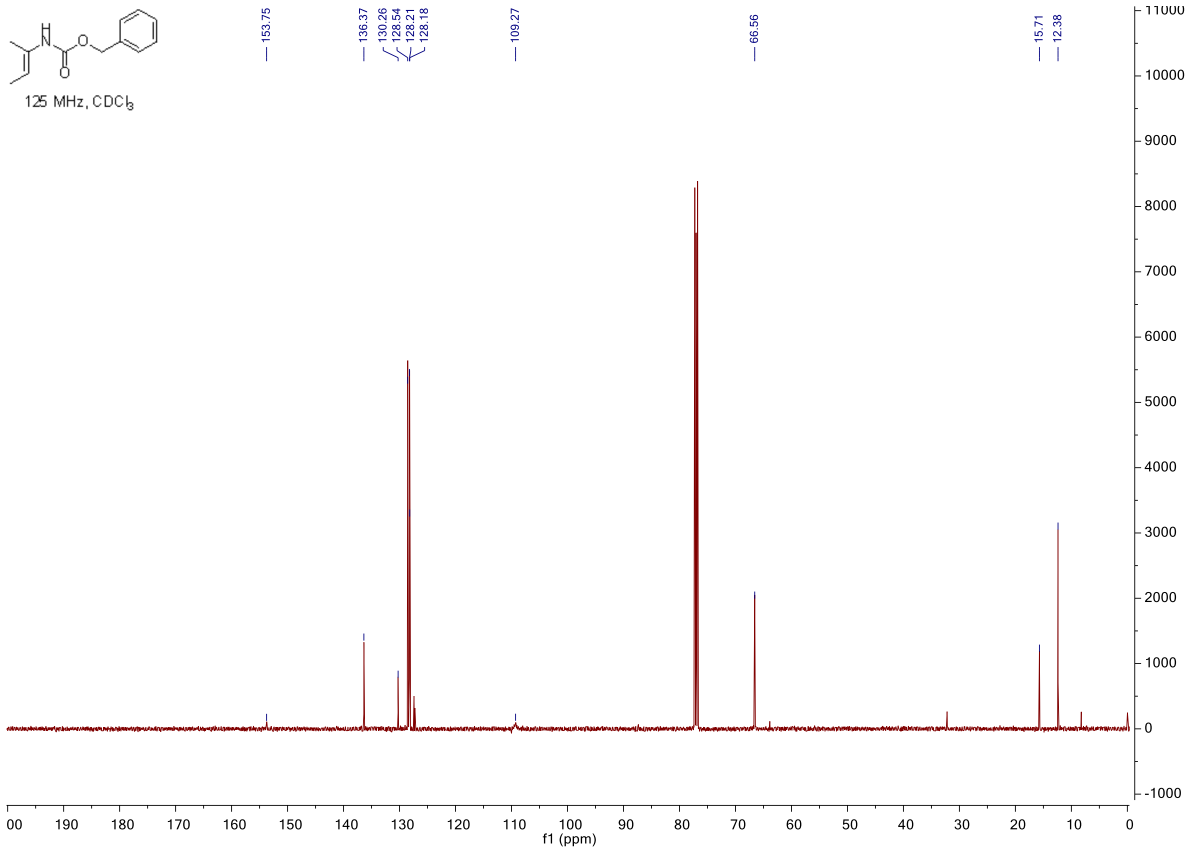

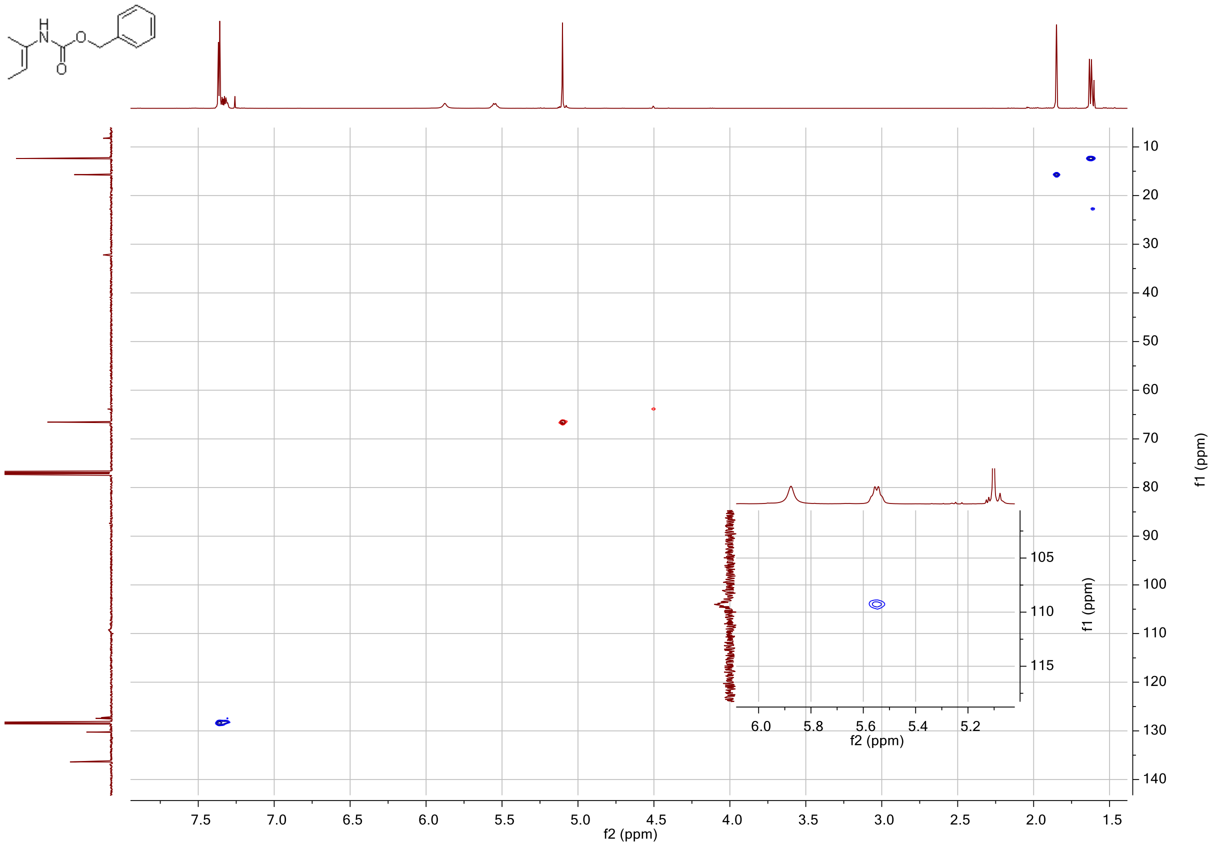
f2 (ppm)
f1 (ppm)

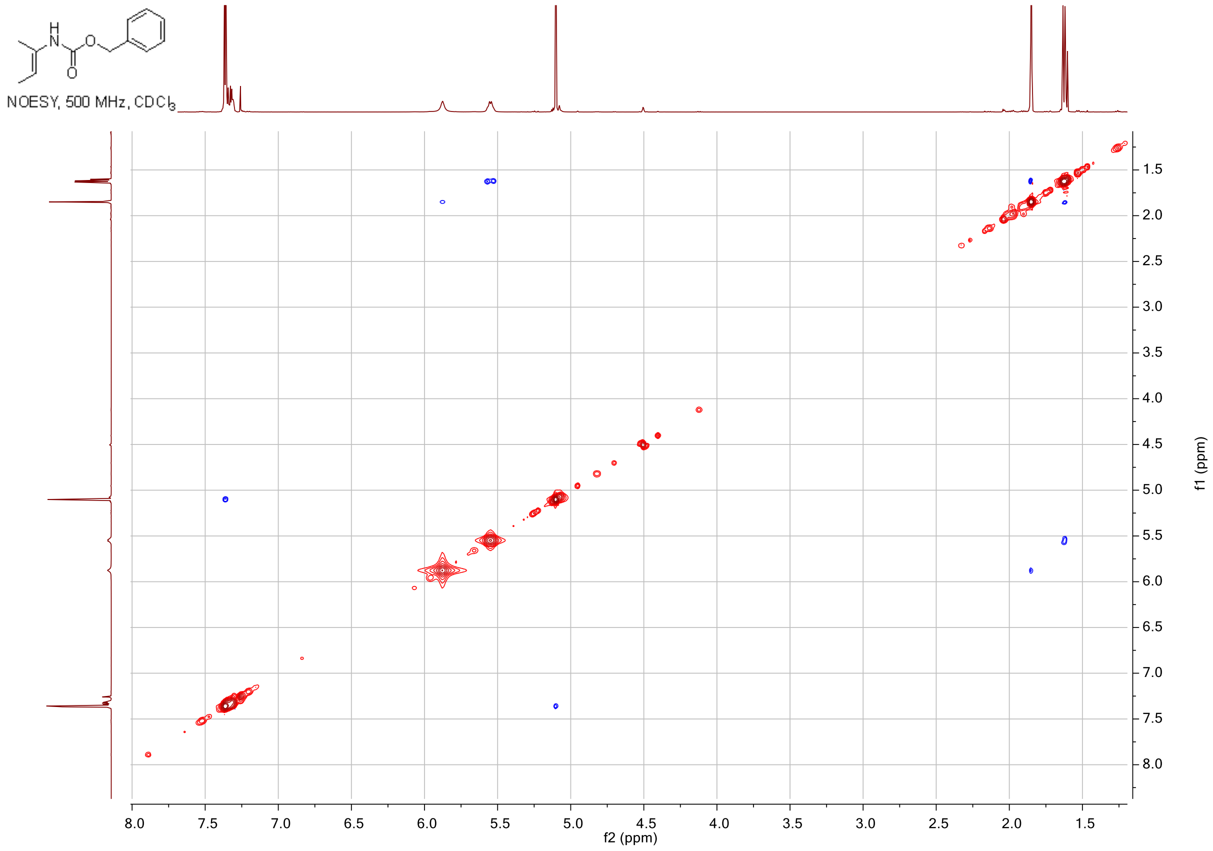
NOESY, 500 MHz, CDCl3
f2 (ppm)
f1 (ppm)

Benzyl cyclobutylcarbamate (3k): ^{1}H, ^{13}C, HSQC

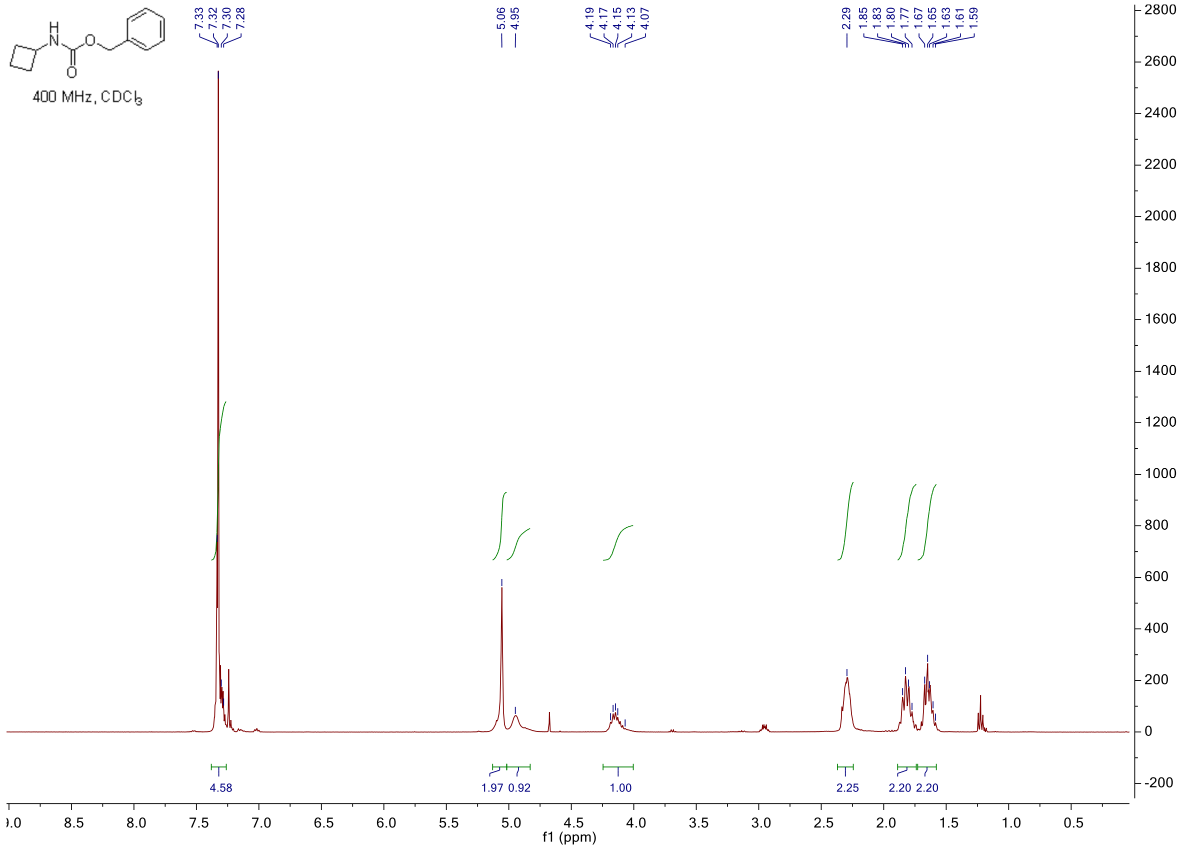

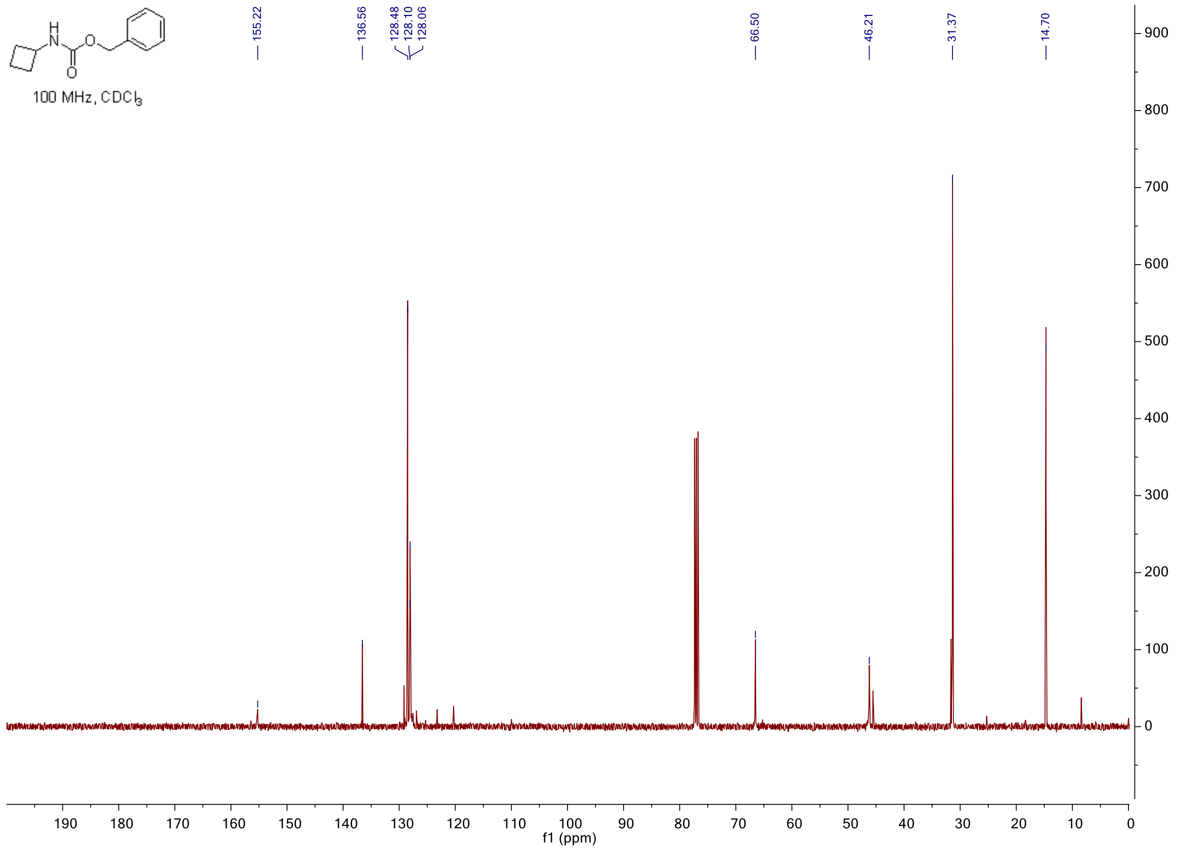

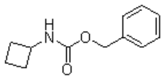

HSQC

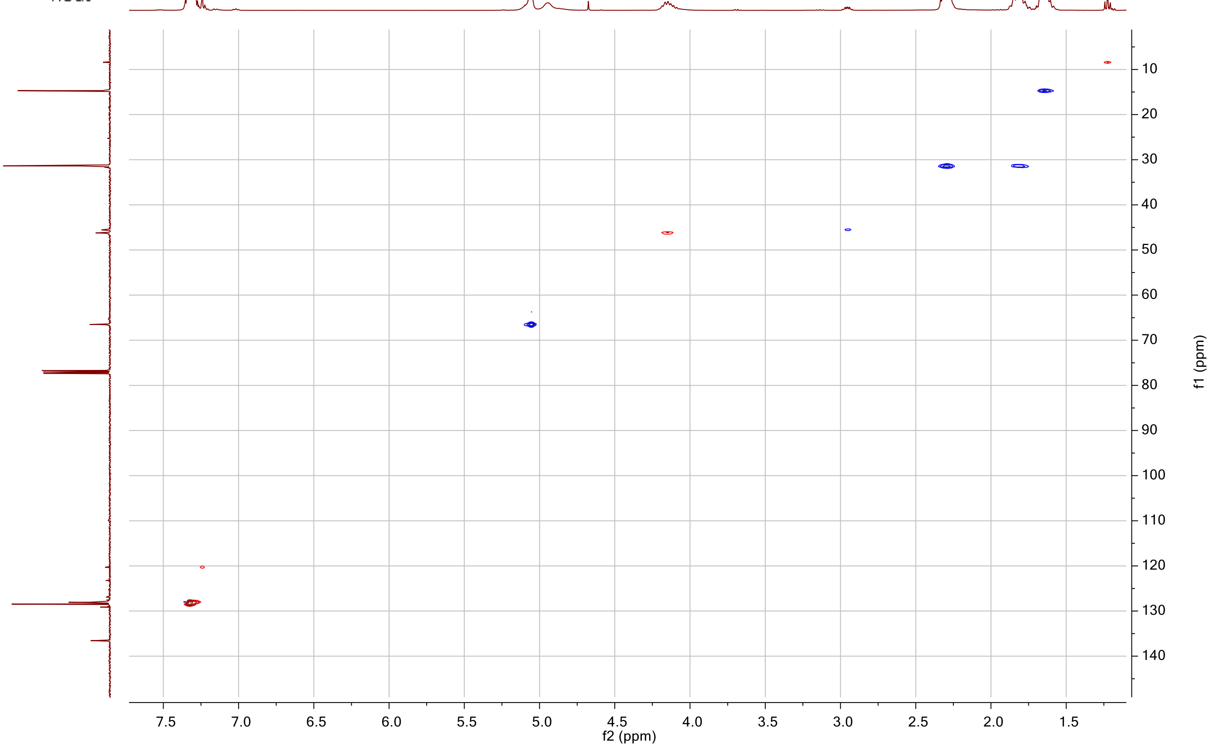

Benzyl (3-oxocyclobutyl)carbamate (3m): ^{1}H, ^{13}C, HSQC

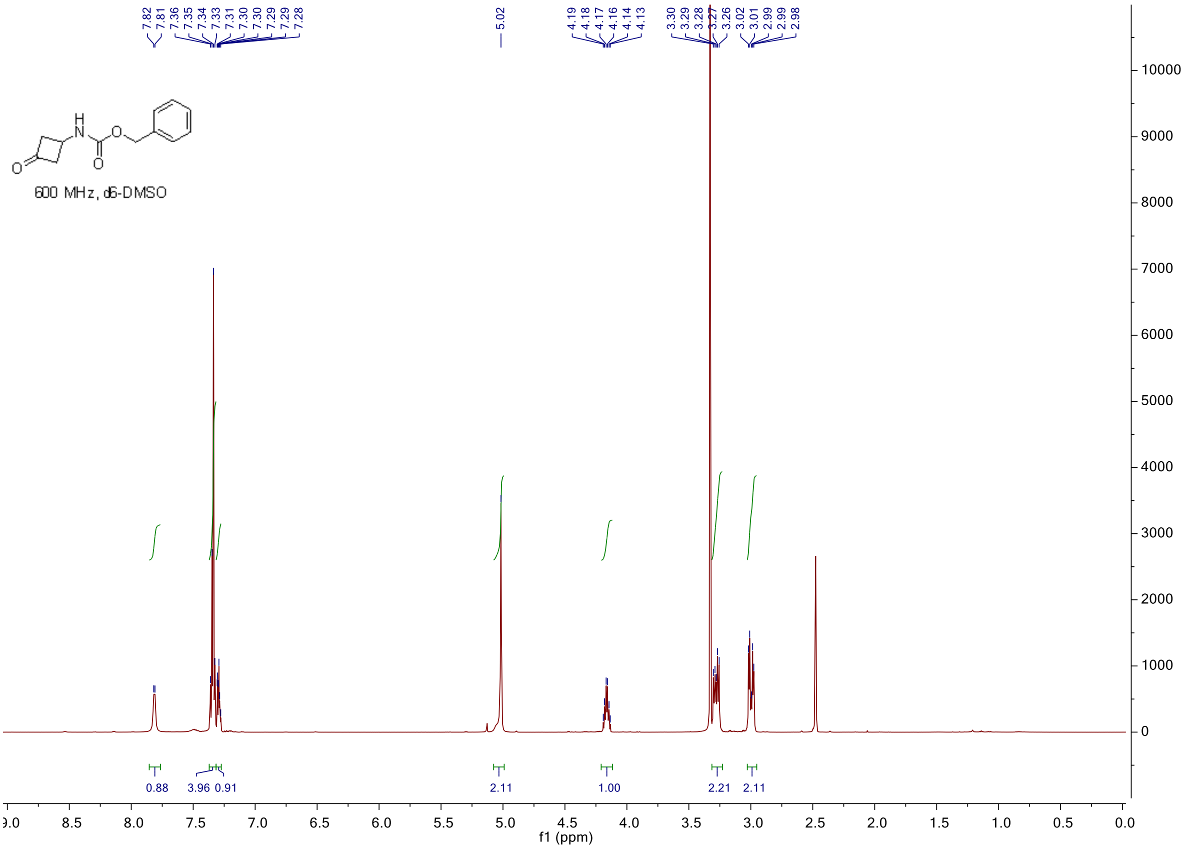

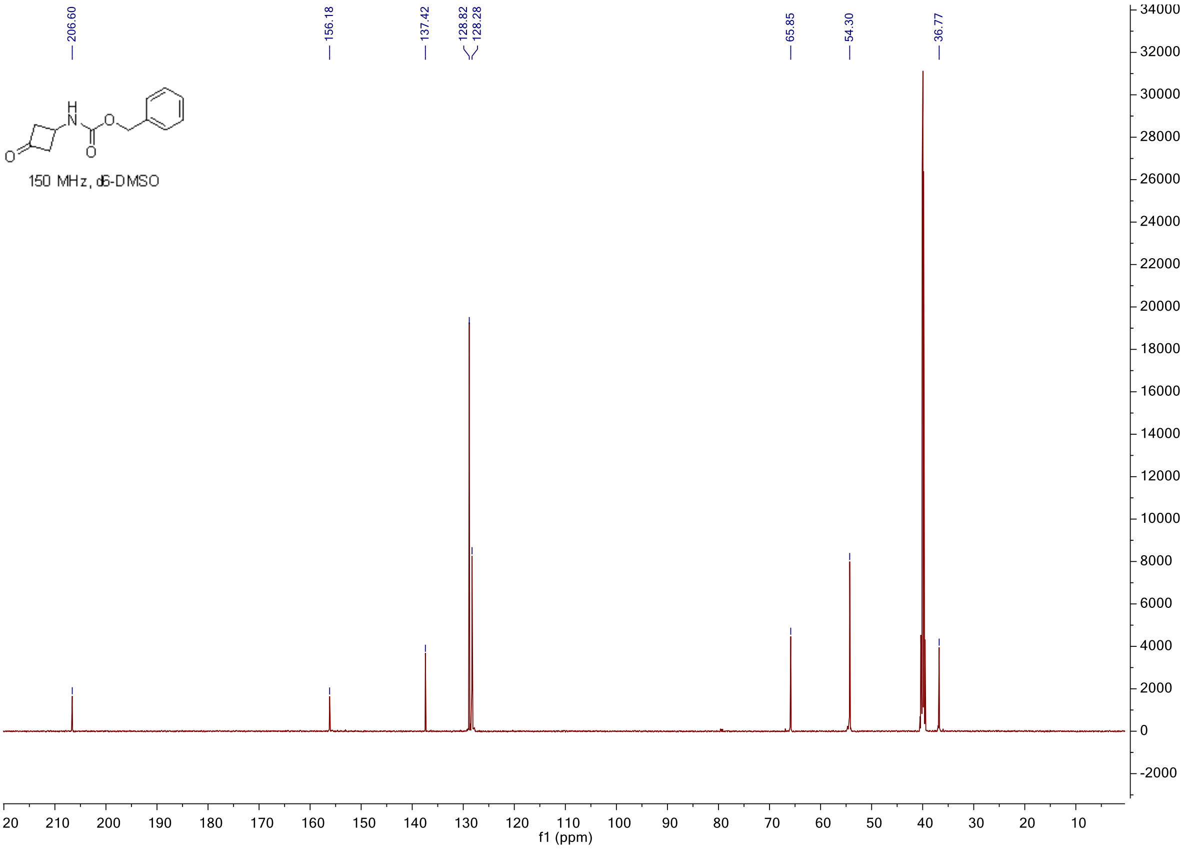

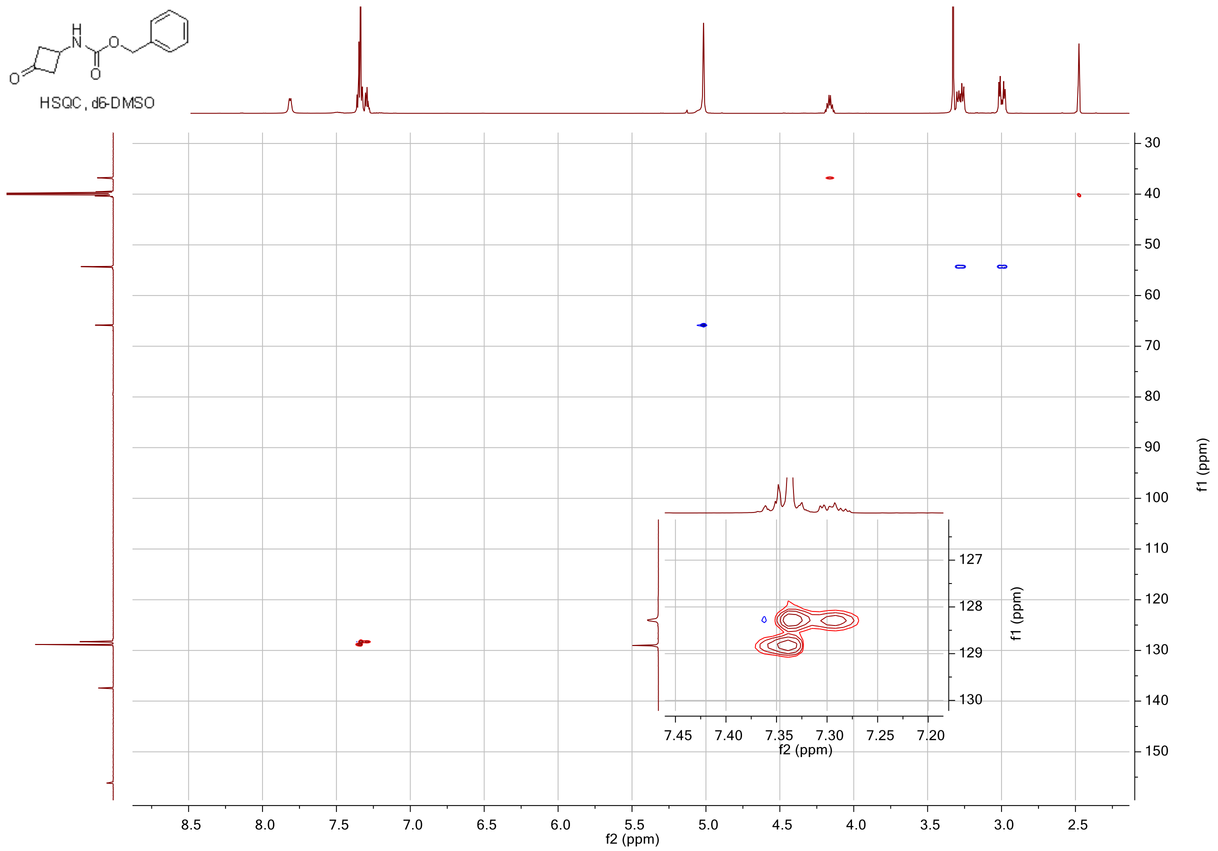
HSQC , d6-DMSO
f1 (ppm)
f2 (ppm)

Benzyl (2-cyanoethyl)(2-fluorophenyl)carbamate (8a): ^{1}H, ^{13}C, ^{19}F, HSQC

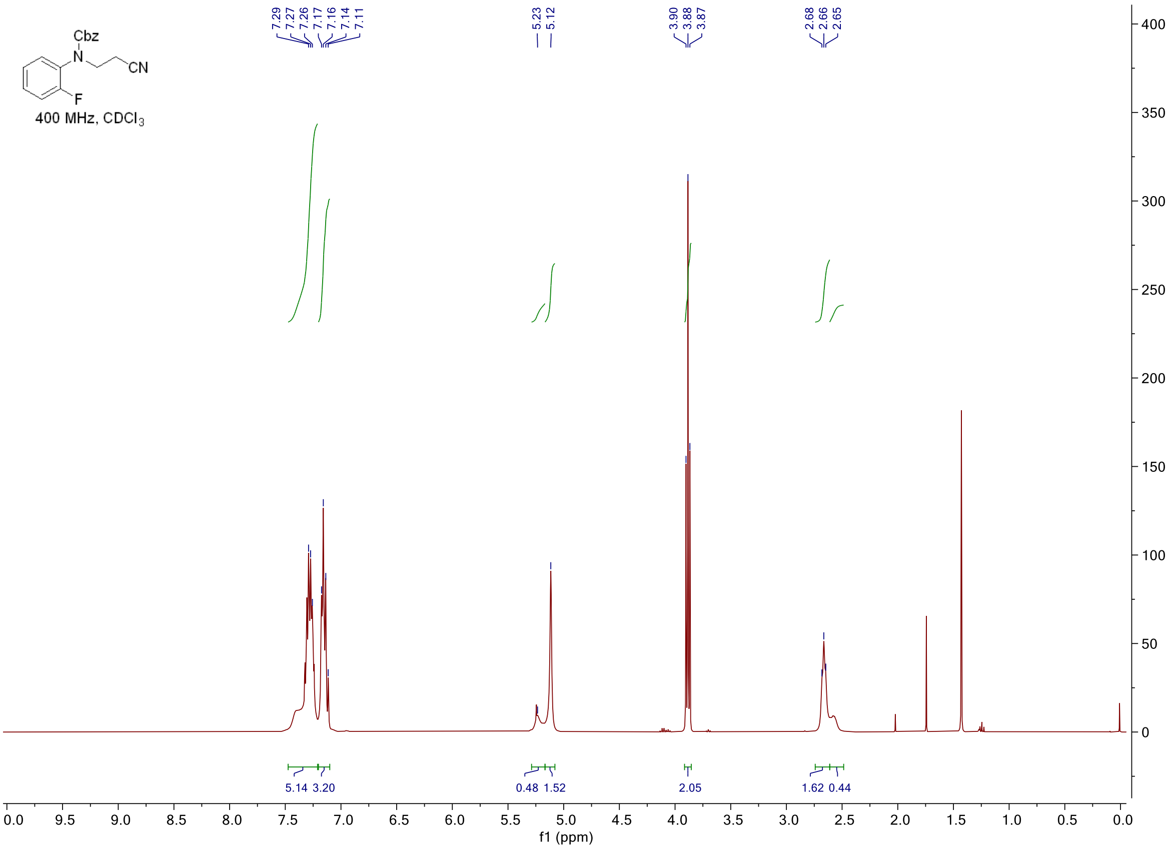

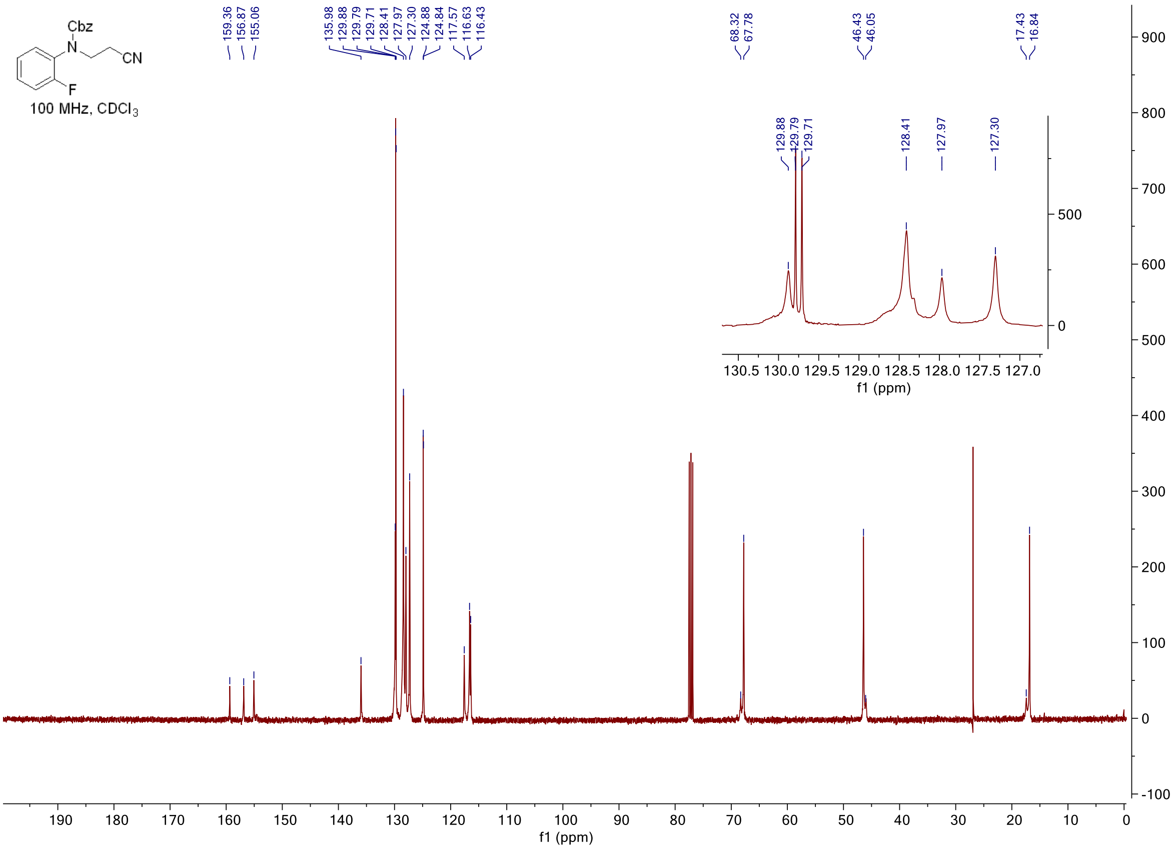

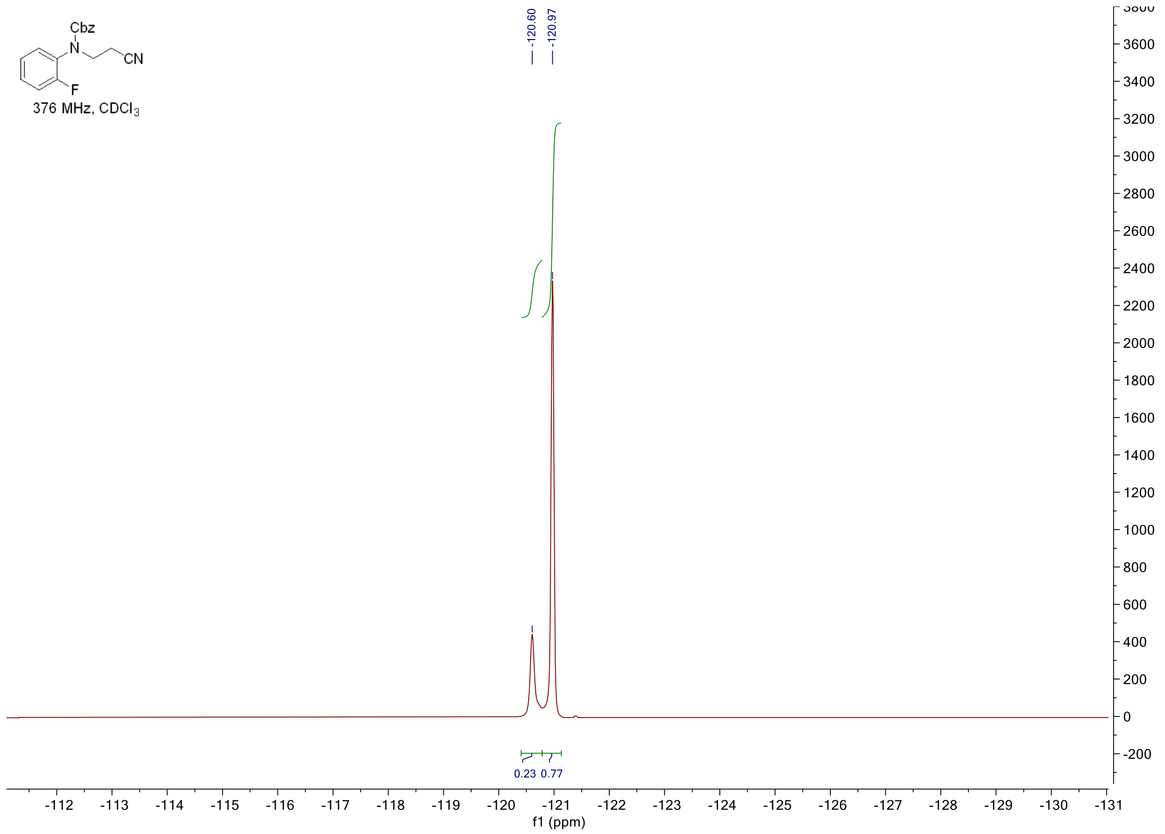
Cbz
N
CN
F
376 MHz, CDCl3
-120.60
-120.97
0.23 0.77
f1 (ppm)

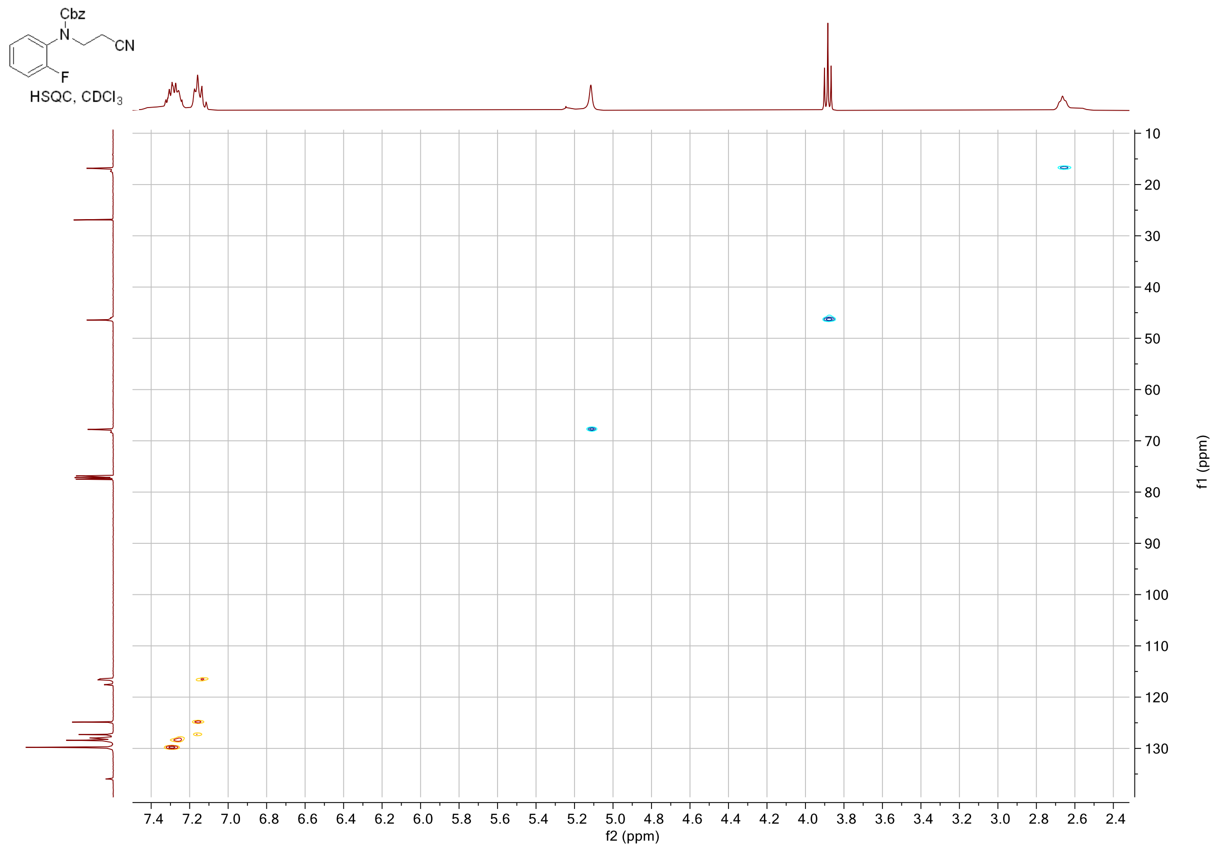
Cbz
N
CN
F
HSQC, CDCl3
f2 (ppm)
f1 (ppm)

Benzyl (2-cyanoethyl)(3,4-dimethoxybenzyl)carbamate (8b): ^{1}H, ^{13}C, HSQC

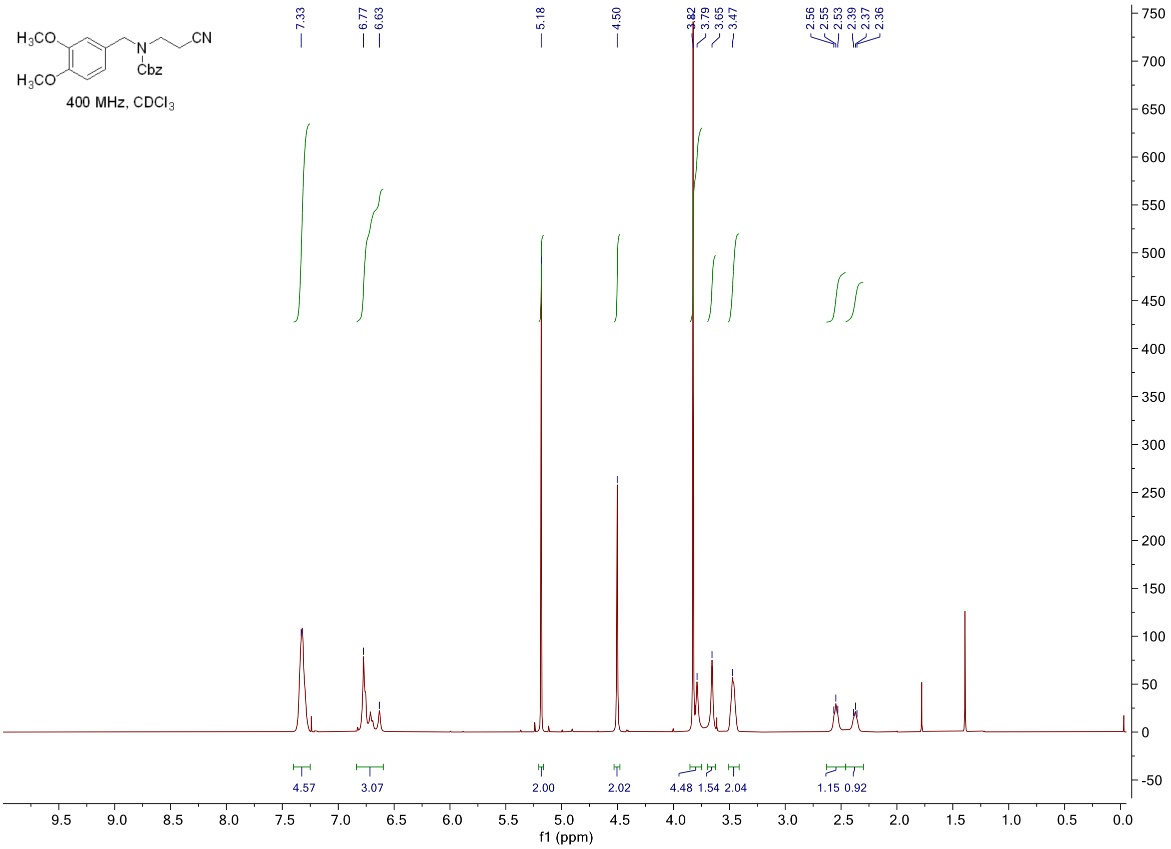

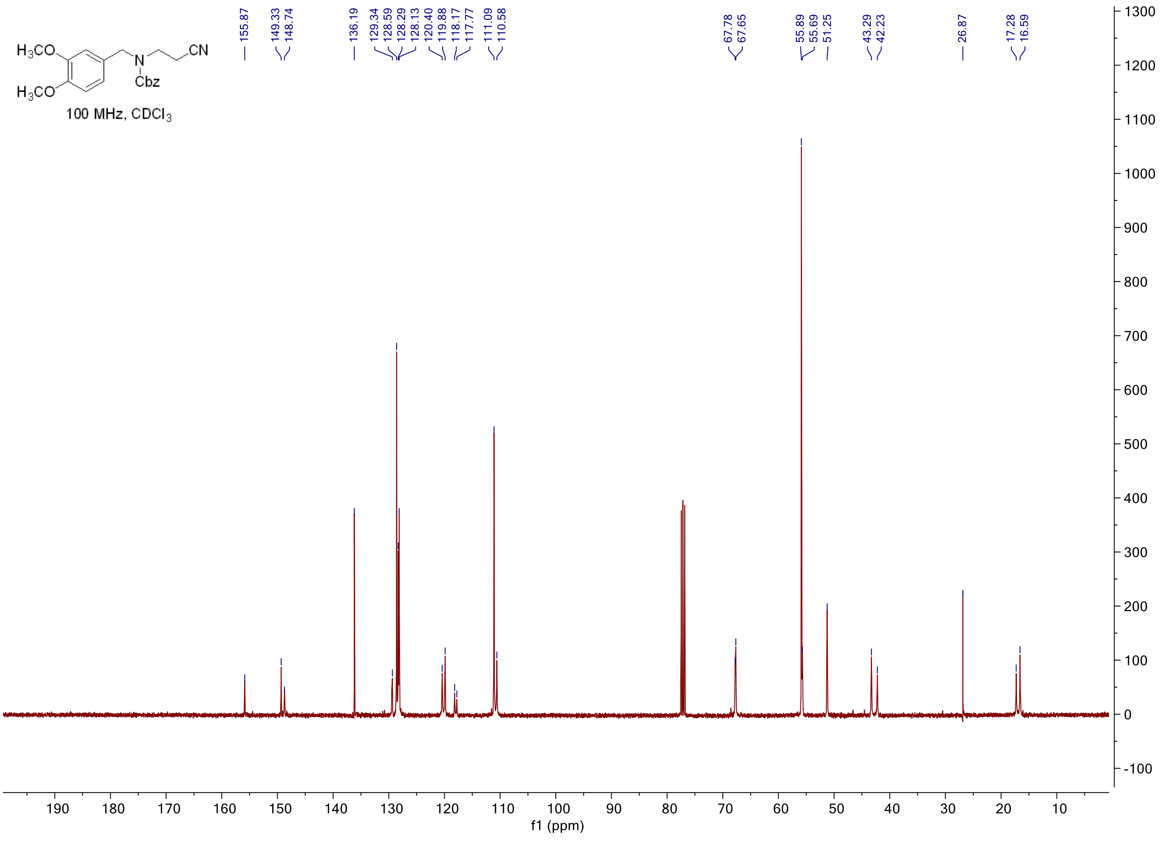

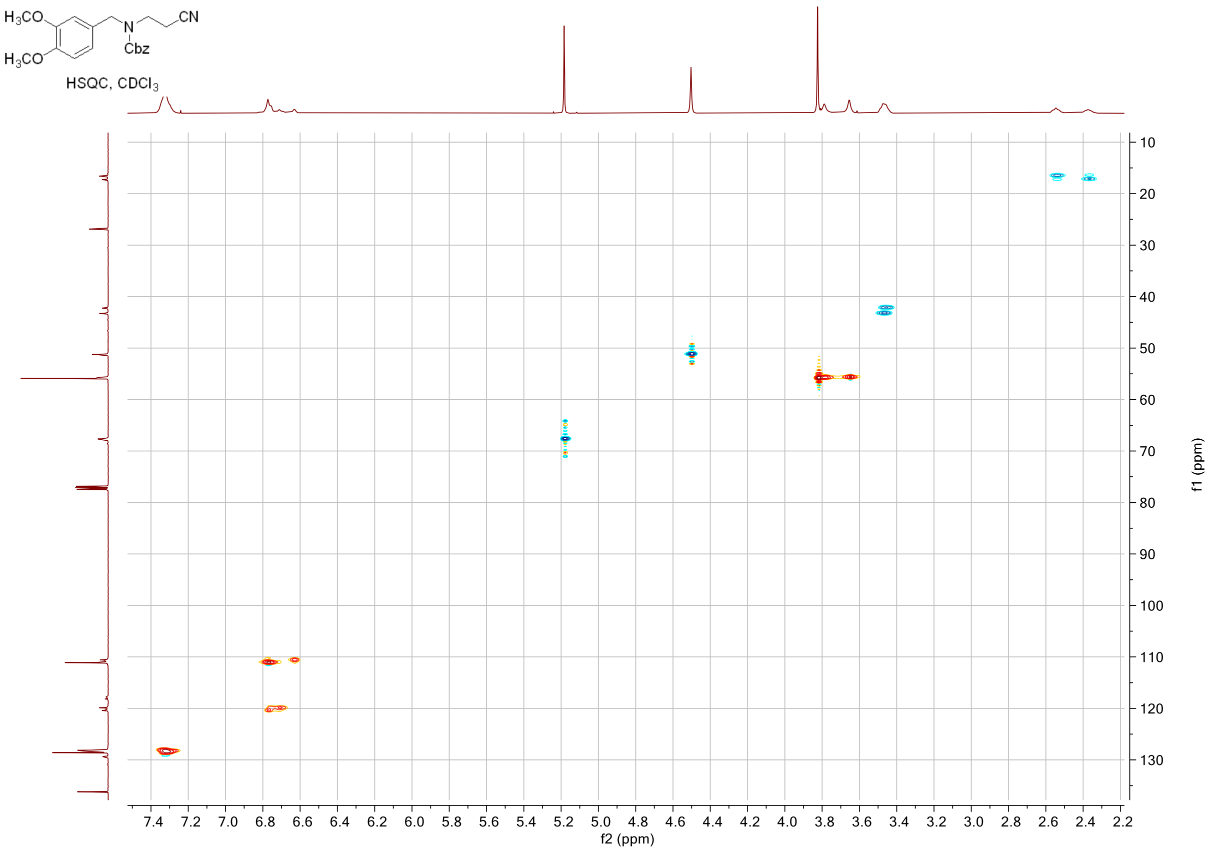

H3CO
H3CO
N
CN
Cbz
HSQC, CDCl3
f1 (ppm)
f2 (ppm)

Benzyl (2-cyanoethyl)(4-(trifluoromethyl)phenyl)carbamate (8c): ^{1}H, ^{13}C, ^{19}F, HSQC

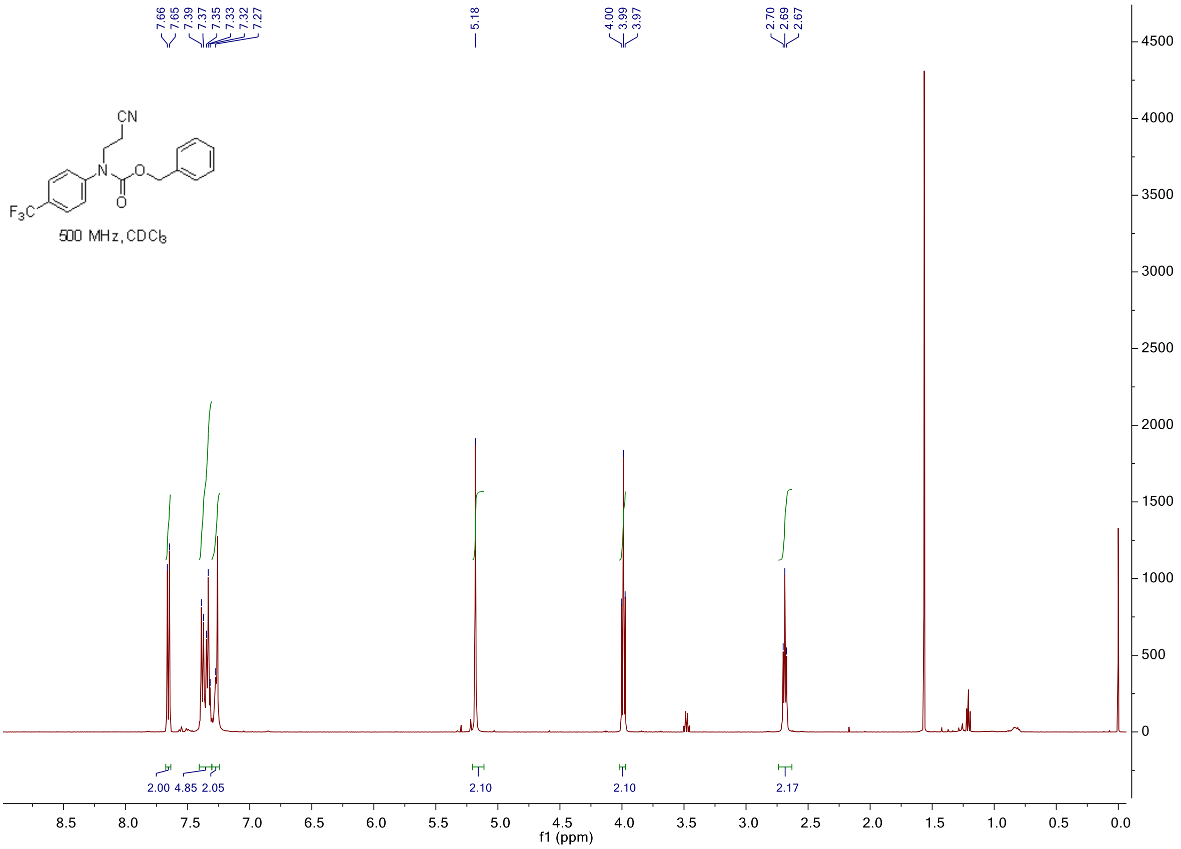

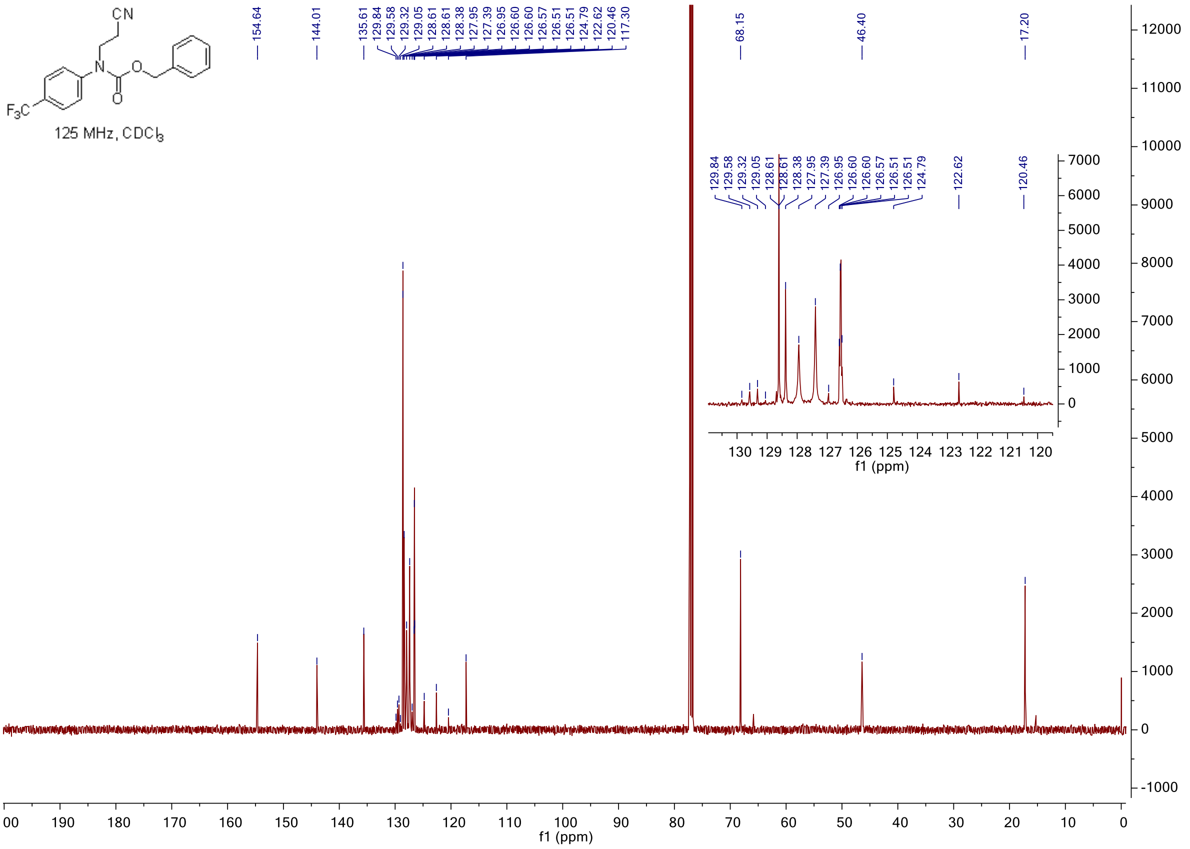

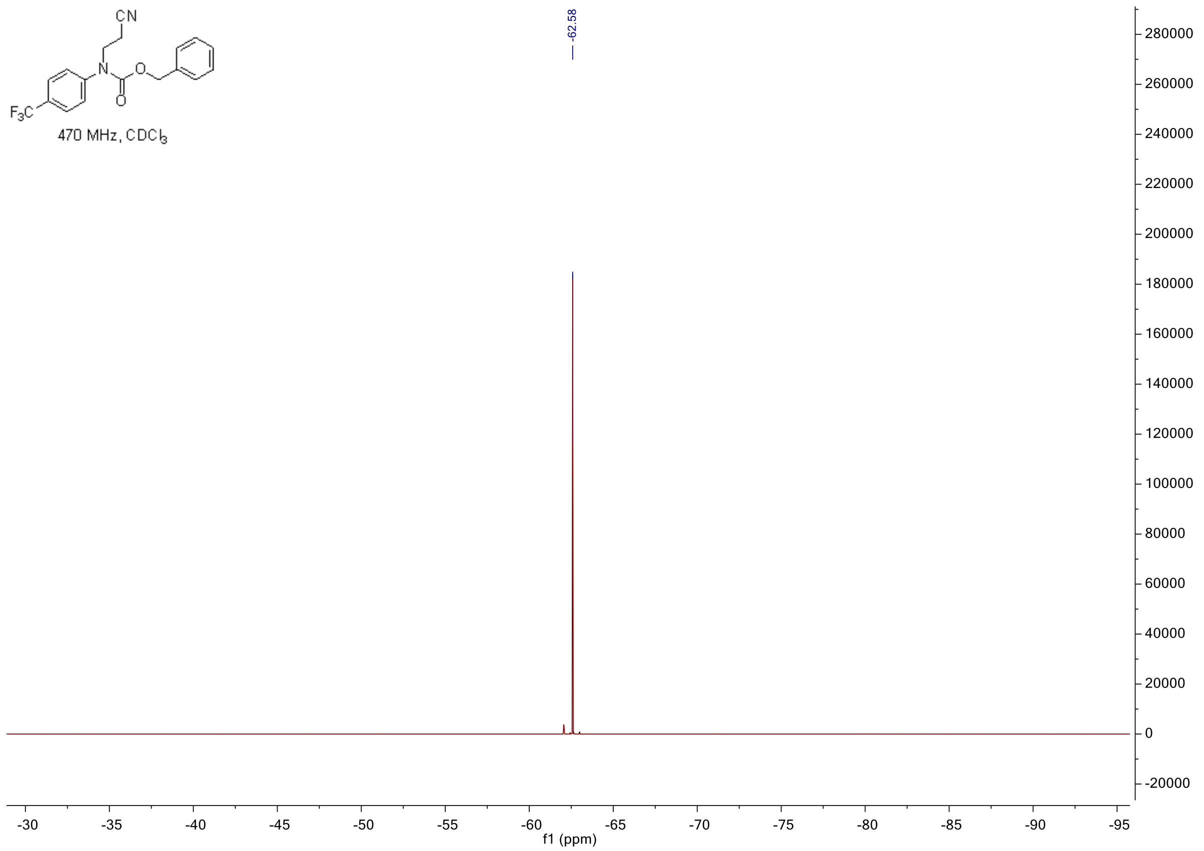
CN
N
O
O
F3C
470 MHz, CDCl3
-62.58
f1 (ppm)

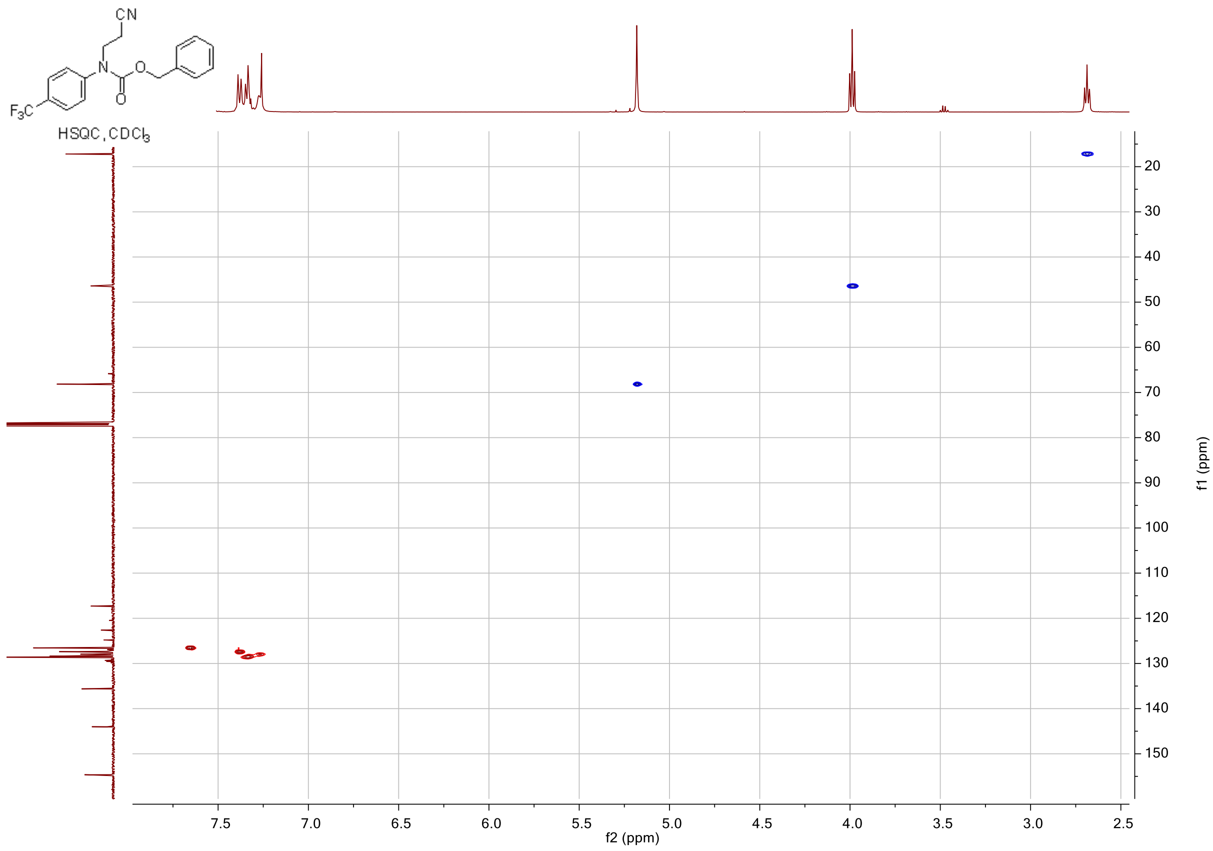
CN
N
O
O
F3C
HSQC, CDCl3
f2 (ppm)
f1 (ppm)

Methyl 3-(((benzyloxy)carbonyl)(4-(trifluoromethyl)phenyl)amino)propanoate (8d): ^{1}H, ^{13}C, ^{19}F, HSQC

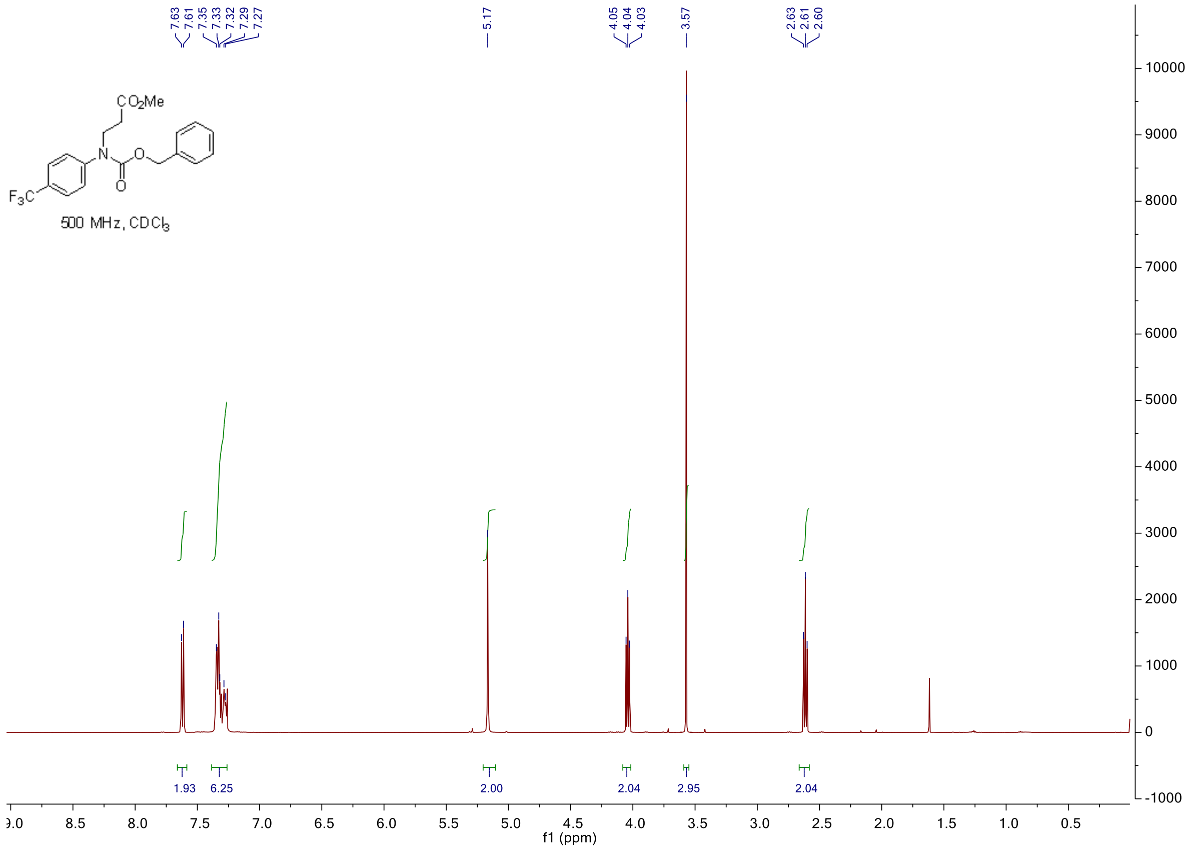

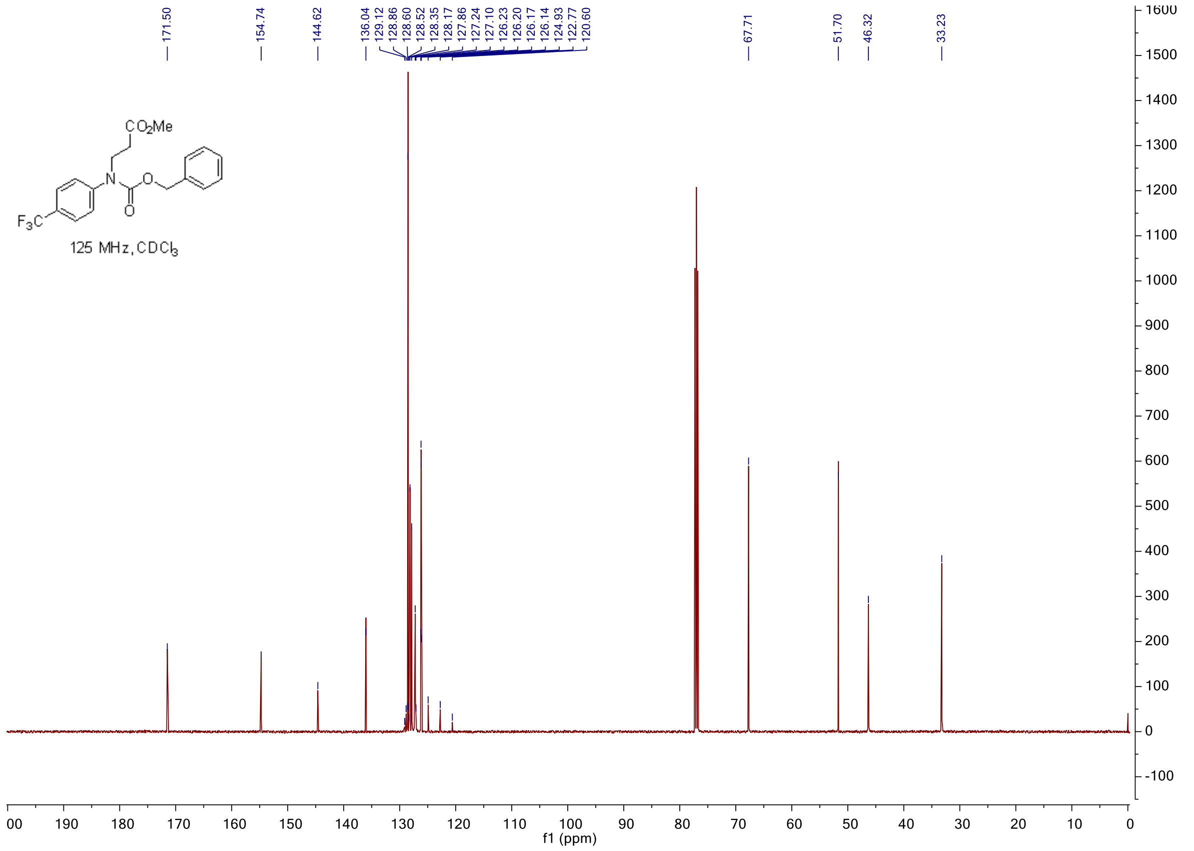

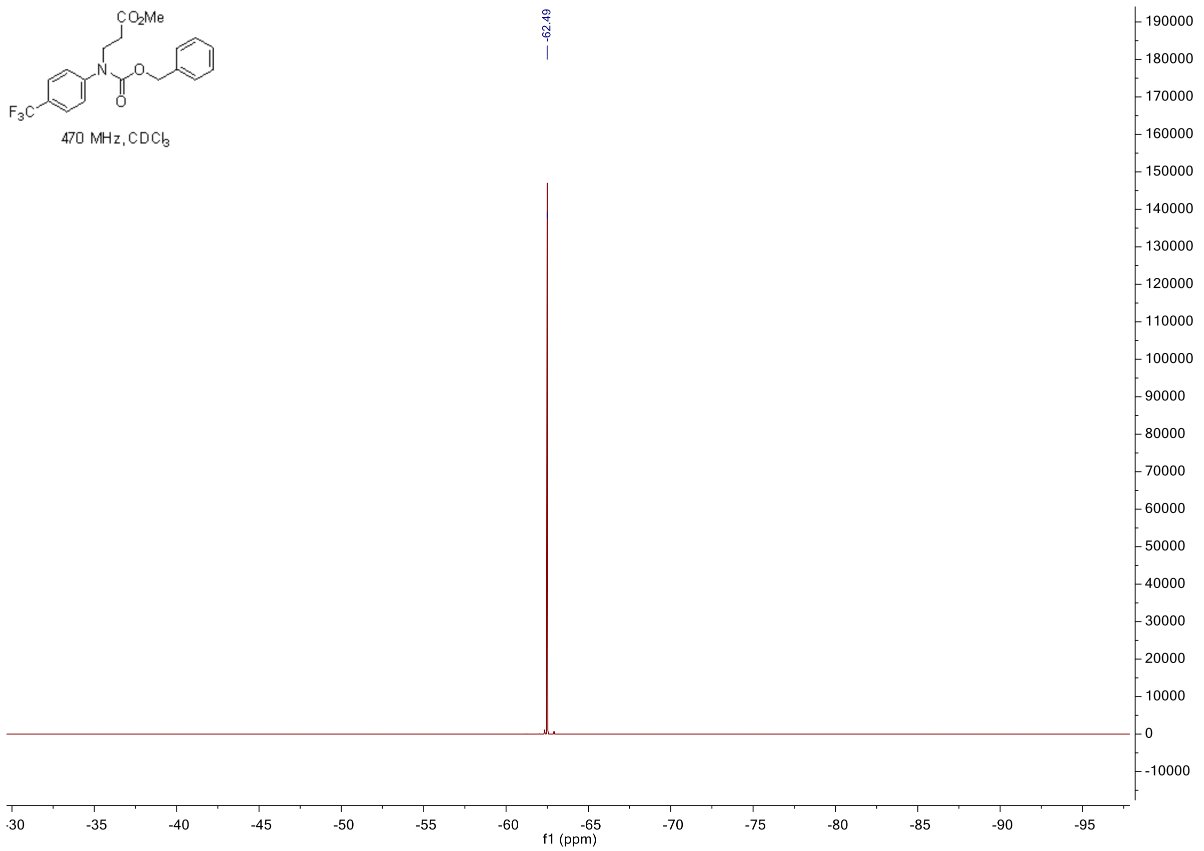

CO2Me
N
O
O
F3C
470 MHz, CDCl3
-62.49
f1 (ppm)

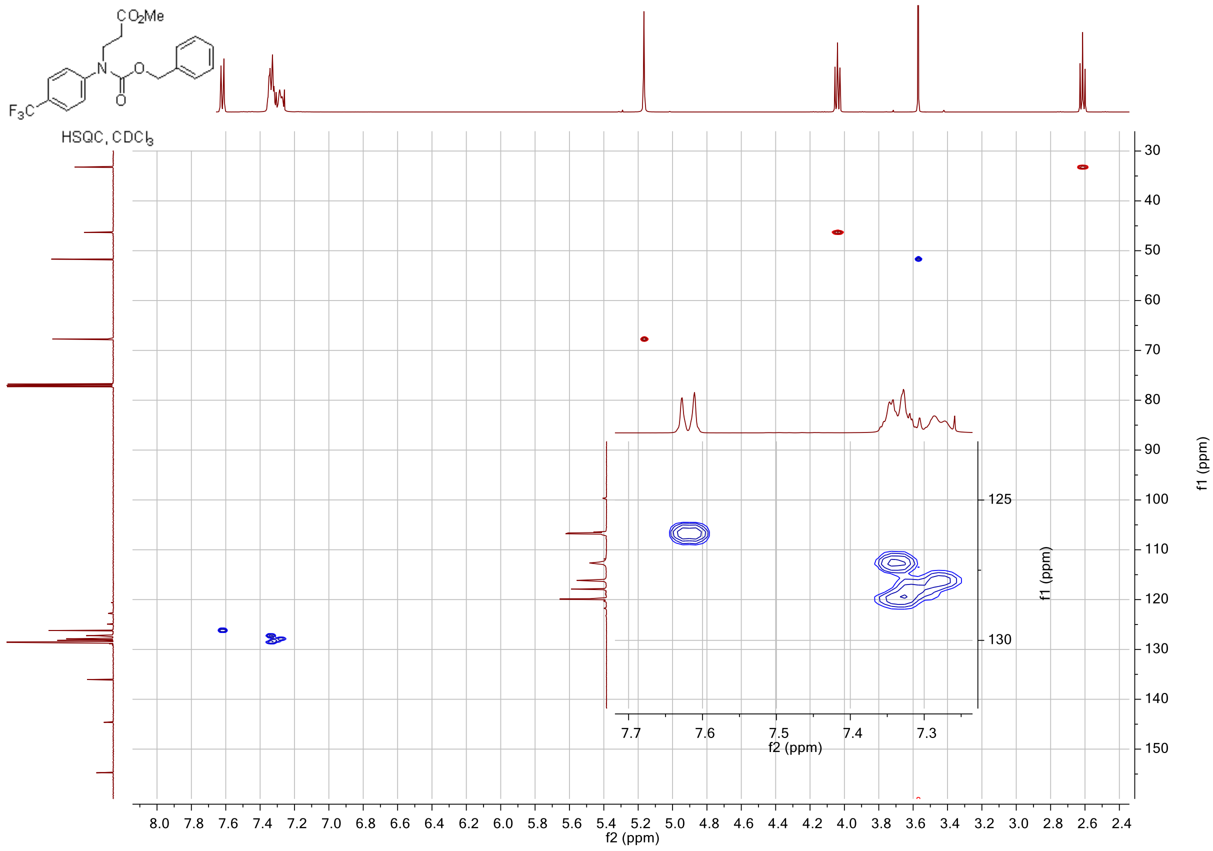

CO2Me
N
O
O
F3C
HSQC, CDCl3
f2 (ppm)
f1 (ppm)

Methyl 3-(((benzyloxy)carbonyl)(2-bromo-5-fluorophenyl)amino)propanoate (8e): ^{1}H, ^{13}C, ^{19}F, HSQC

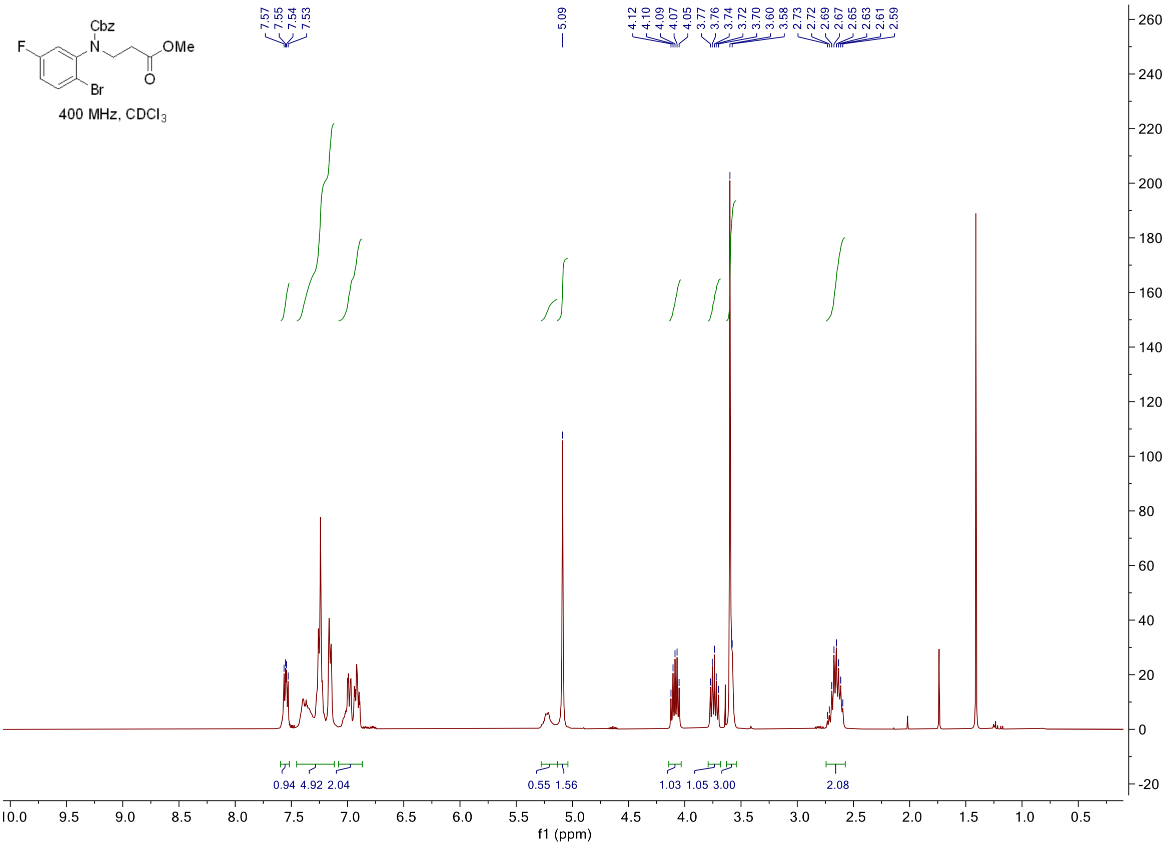

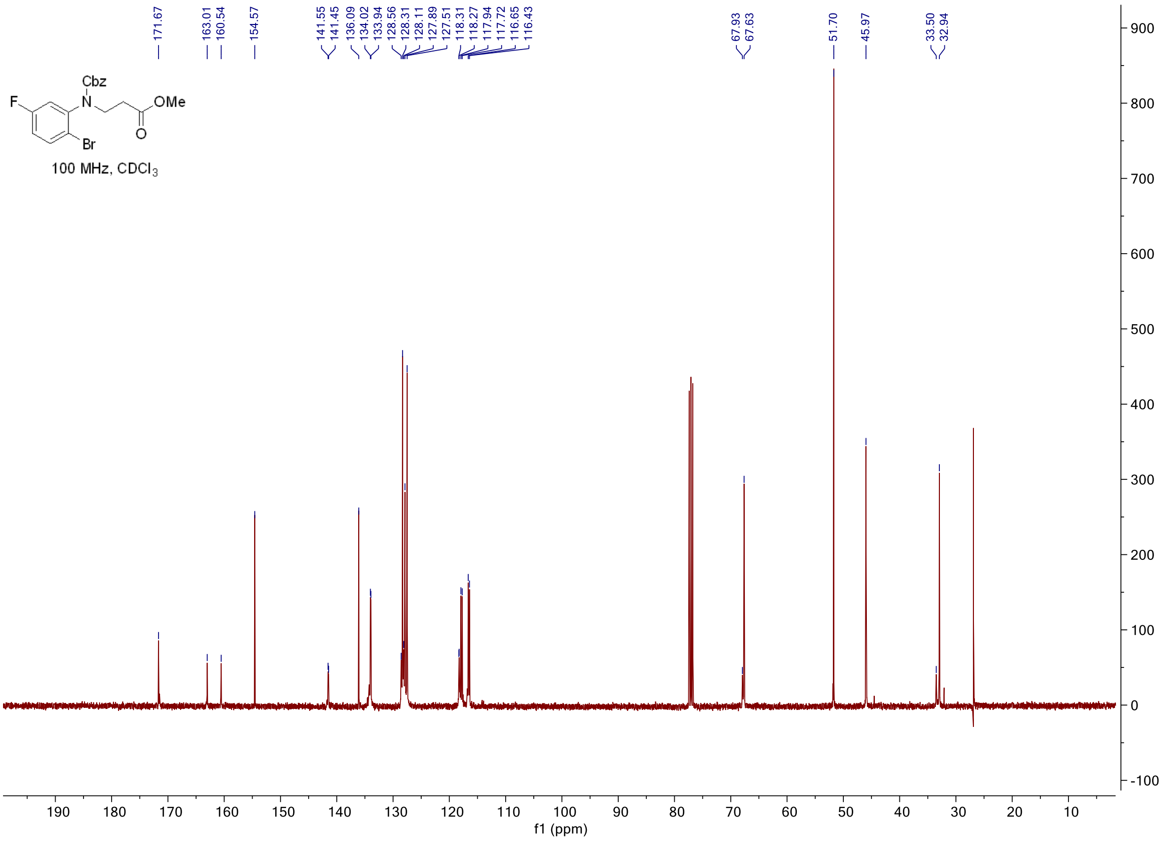

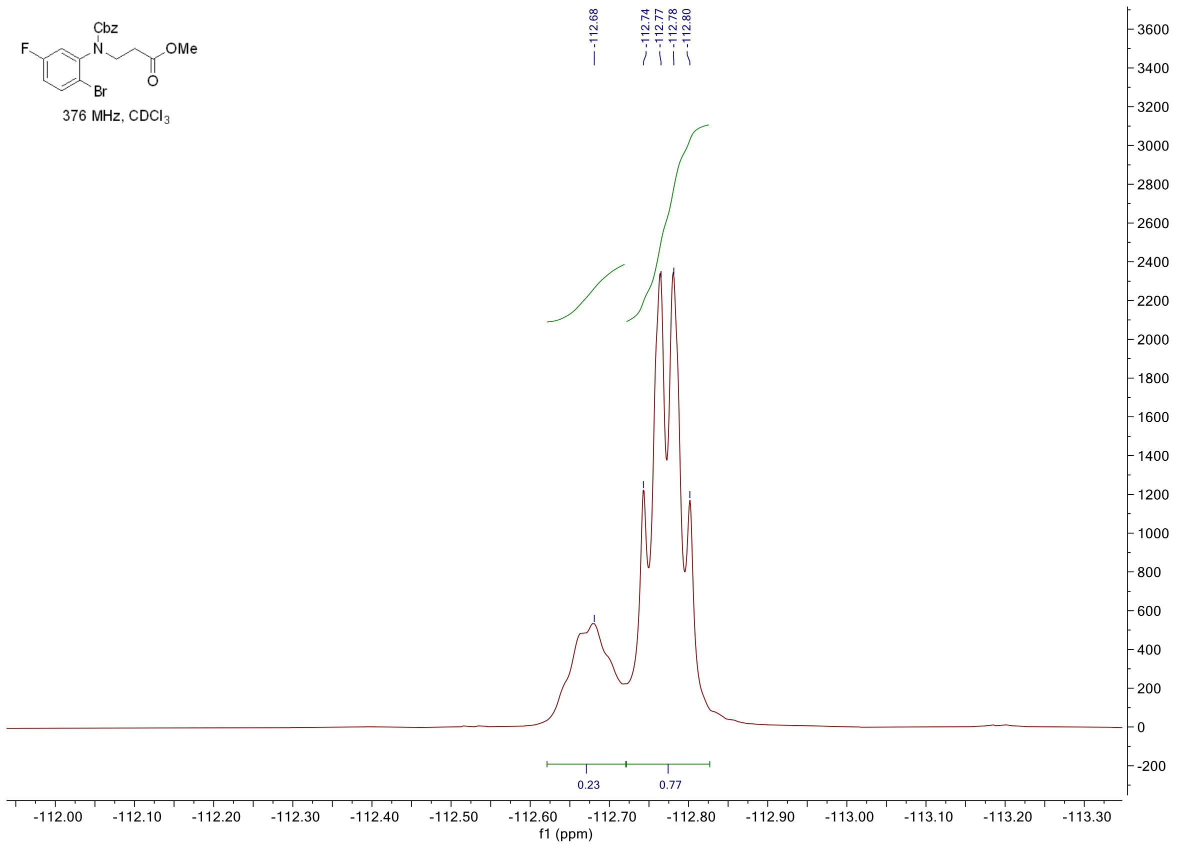
Cbz
N
F
OMe
O
Br
376 MHz, CDCl3
-112.68
-112.74
-112.77
-112.78
-112.80
0.23
0.77
f1 (ppm)

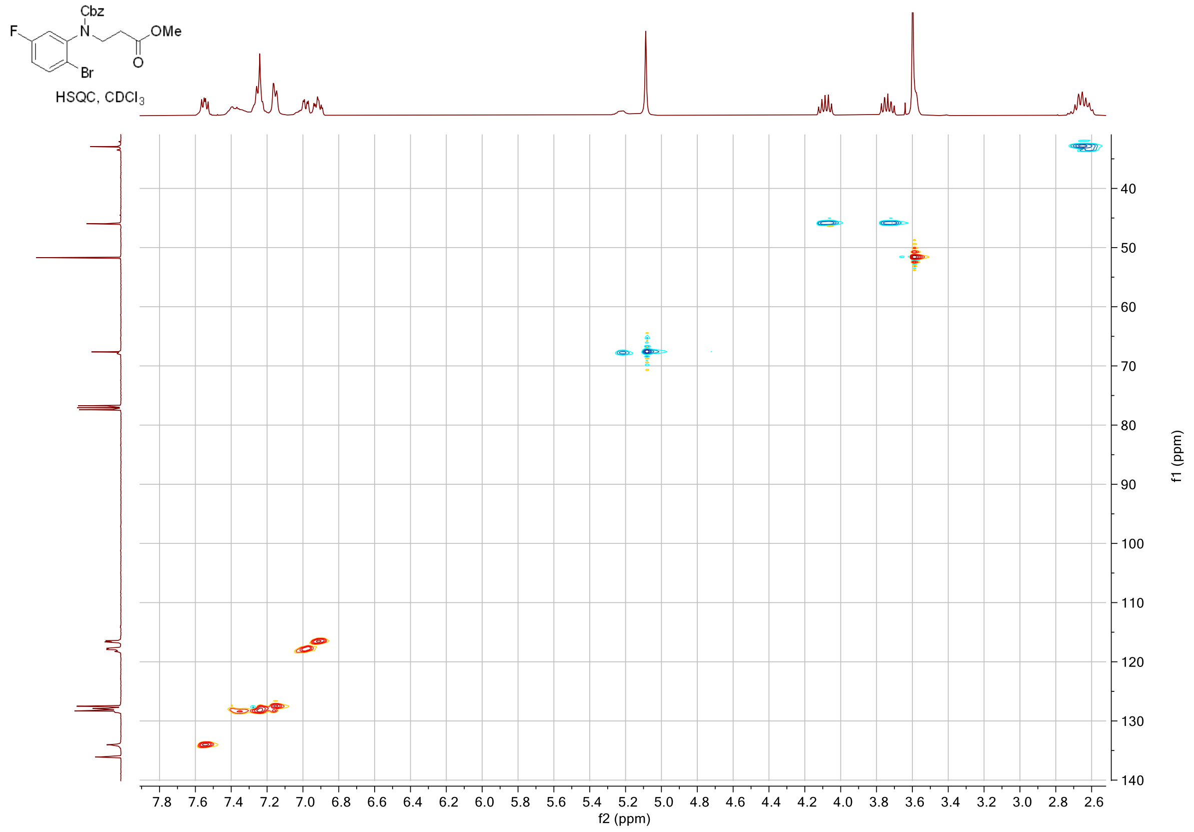
Cbz
N
F
OMe
O
Br
HSQC, CDCl3
f2 (ppm)
f1 (ppm)

Methyl 3-(((benzyloxy)carbonyl)(4-(trifluoromethoxy)phenyl)amino)propanoate (8f): ^{1}H, ^{13}C, ^{19}F, HSQC

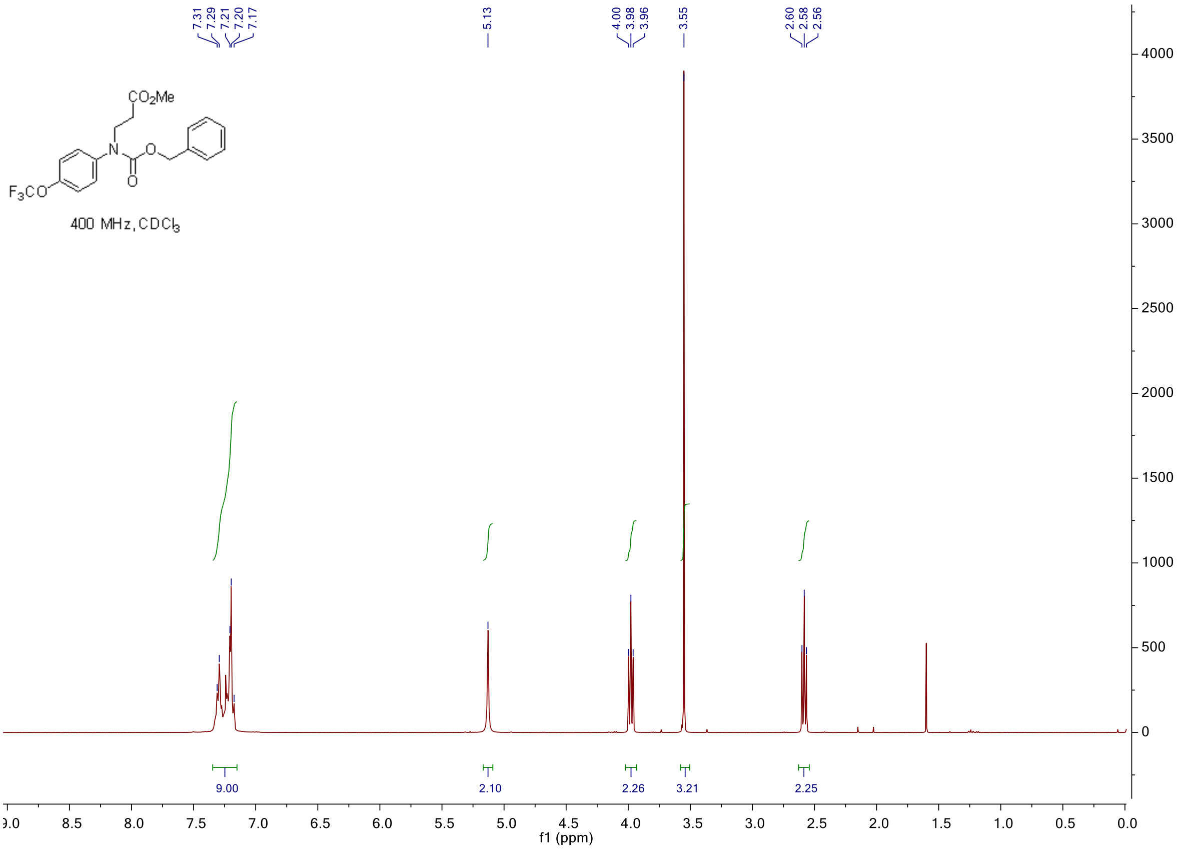

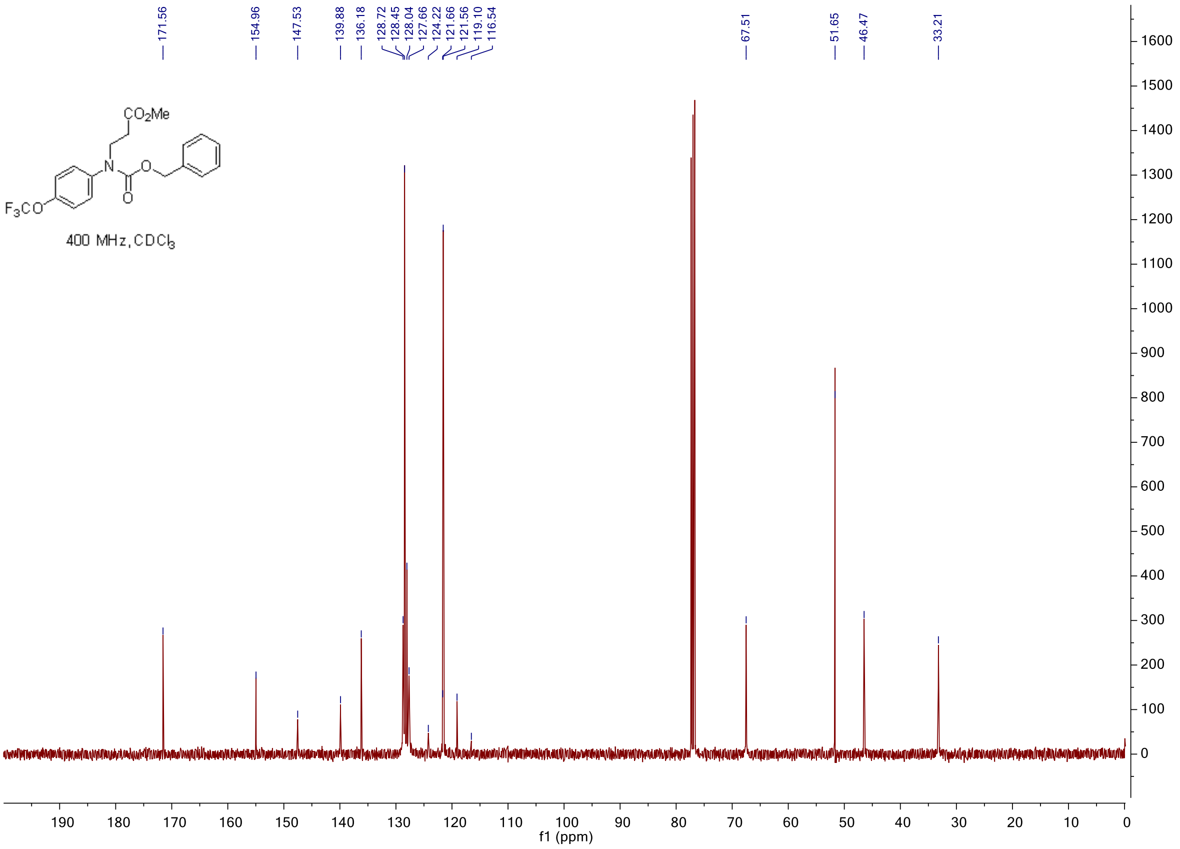

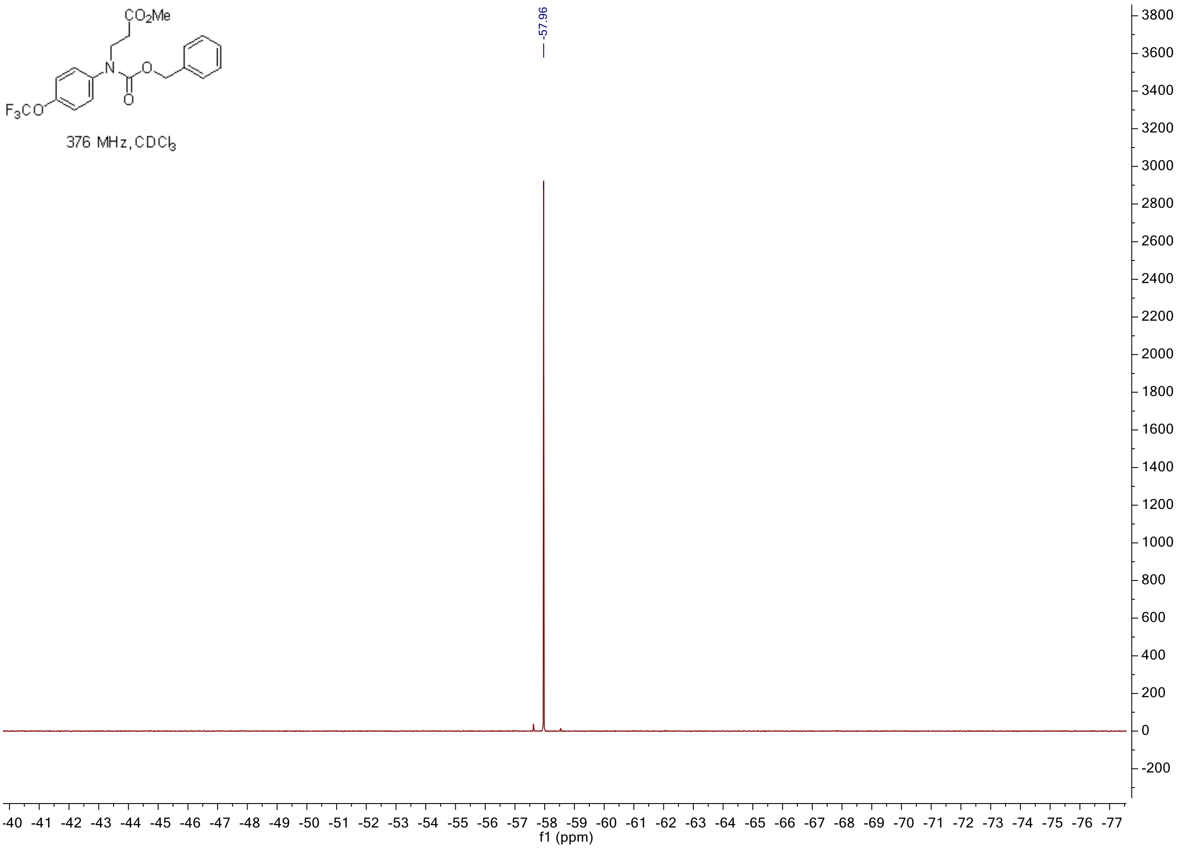
CO2Me
N
O
O
F3CO
376 MHz, CDCl3
-57.96
f1 (ppm)

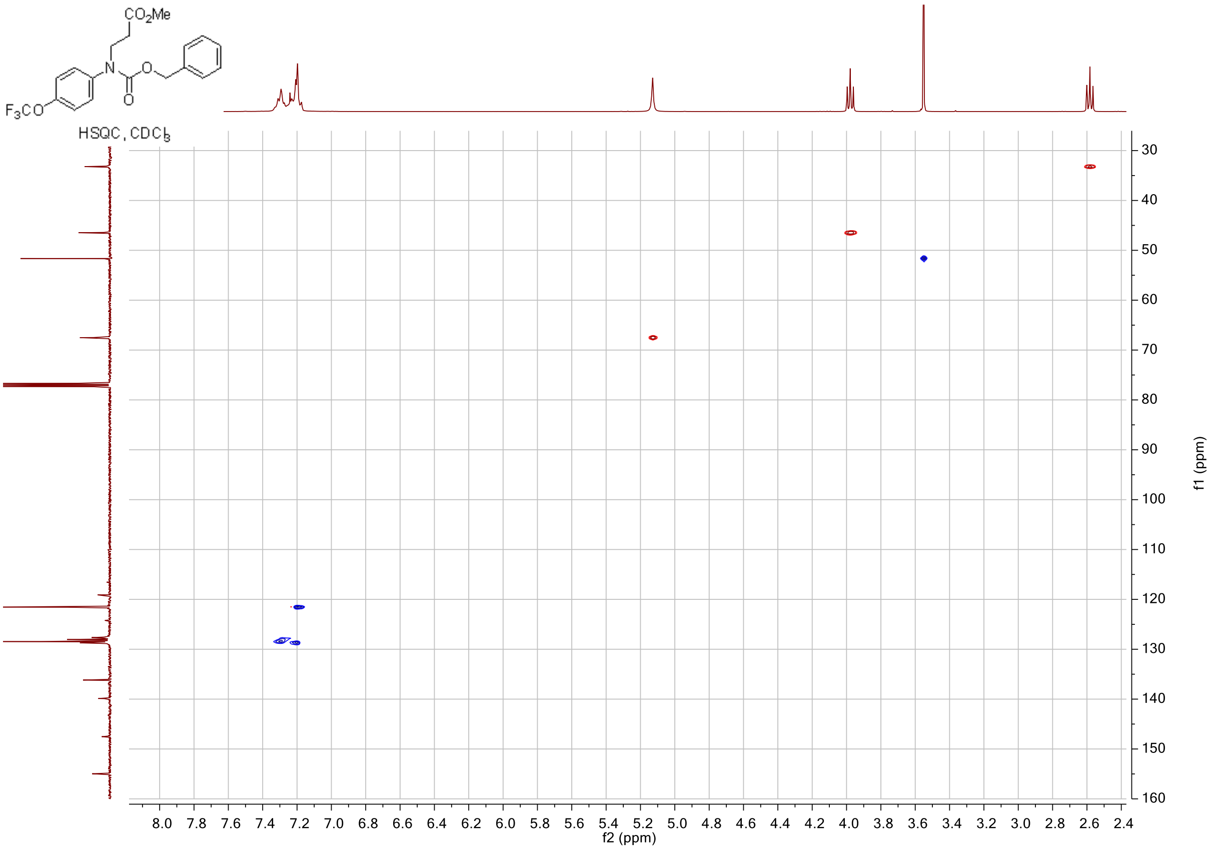
CO2Me
N
O
O
F3CO
HSQC, CDCl3
f2 (ppm)
f1 (ppm)

3-(((Benzyloxy)carbonyl)(4-(trifluoromethyl)phenyl)amino)propanoic acid (9d): ^{1}H, ^{13}C, ^{19}F, HSQC

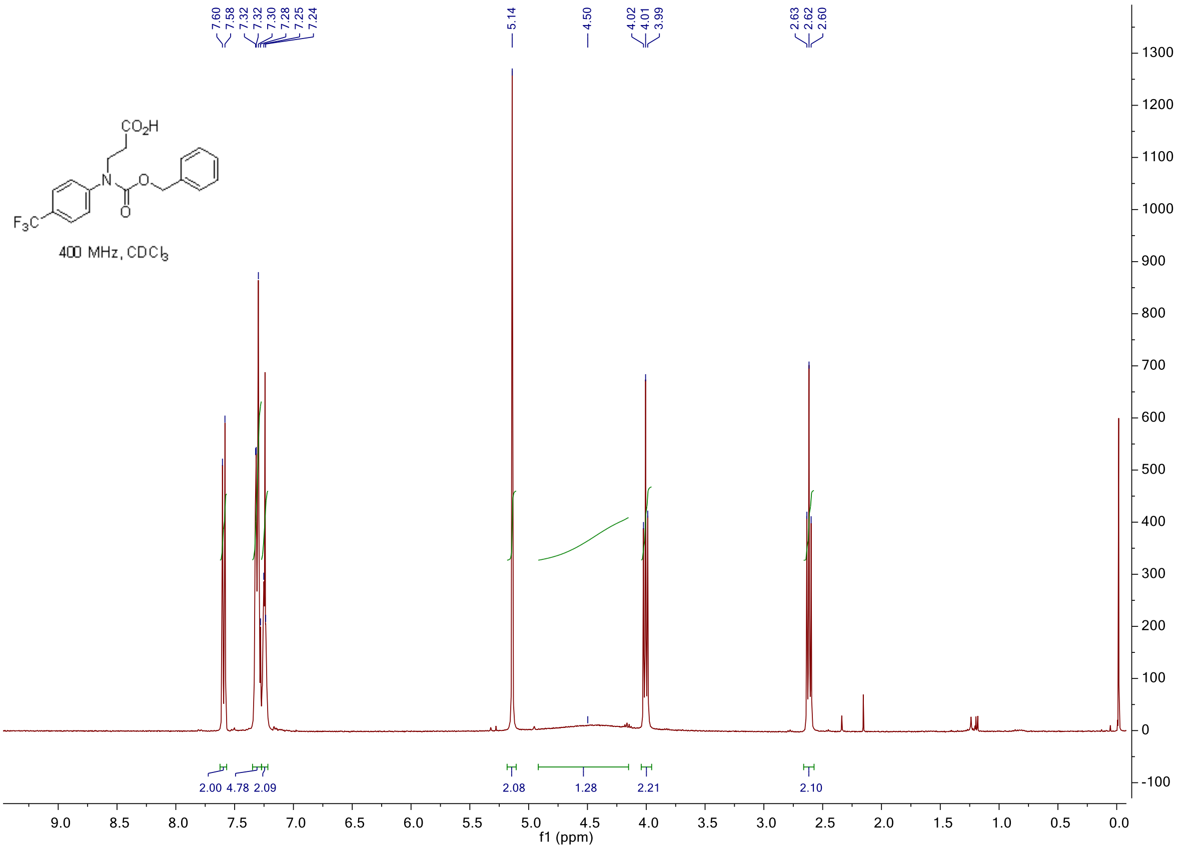

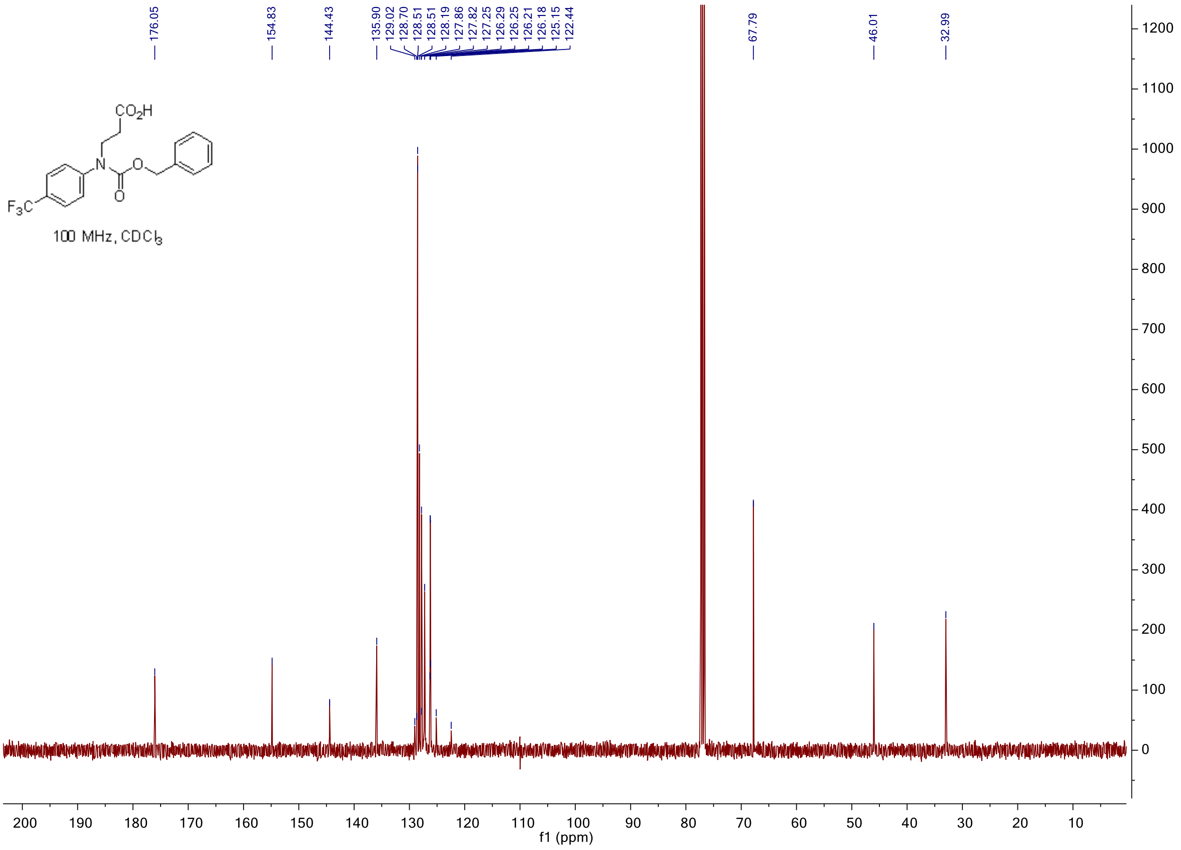

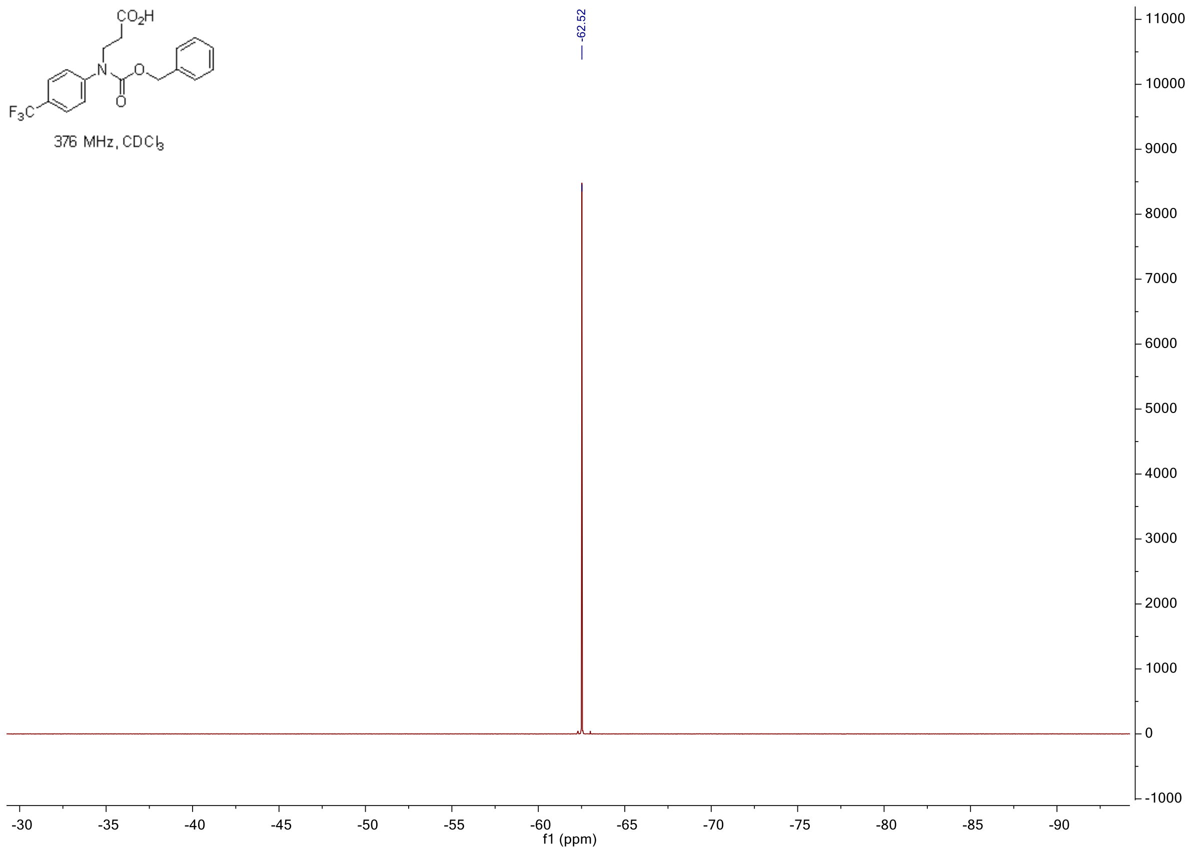

CO2H
N
O
O
F3C
376 MHz, CDCl3
-62.52
f1 (ppm)

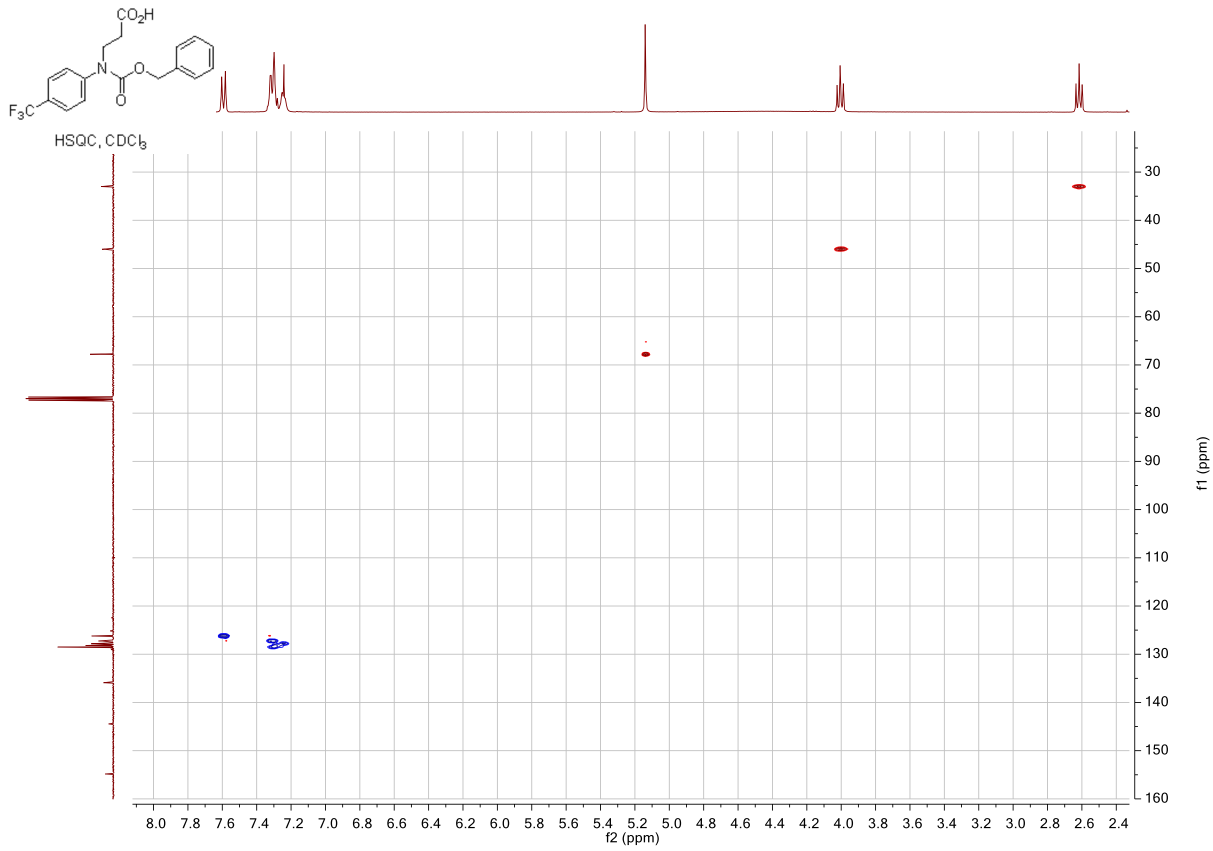

CO2H
N
O
O
F3C
HSQC, CDCl3
f2 (ppm)
f1 (ppm)
